# Engineered cytokine/antibody fusion proteins improve IL-2 delivery to pro-inflammatory cells and promote antitumor activity

**DOI:** 10.1172/jci.insight.173469

**Published:** 2024-09-24

**Authors:** Elissa K. Leonard, Jakub Tomala, Joseph R. Gould, Michael I. Leff, Jian-Xin Lin, Peng Li, Mitchell J. Porter, Eric R. Johansen, Ladaisha Thompson, Shanelle D. Cao, Shenda Hou, Tereza Henclova, Maros Huliciak, Paul R. Sargunas, Charina S. Fabilane, Ondřej Vaněk, Marek Kovar, Bohdan Schneider, Giorgio Raimondi, Warren J. Leonard, Jamie B. Spangler

**Affiliations:** 1 Department of Biomedical Engineering, Johns Hopkins University School of Medicine, Baltimore, Maryland, USA.; 2 Institute of Biotechnology of the Academy of Sciences of the Czech Republic, Vestec, Czech Republic.; 3 Department of Chemical & Biomolecular Engineering and; 4 Department of Biology, Johns Hopkins University, Baltimore, Maryland, USA.; 5 Laboratory of Molecular Immunology, National Heart, Lung, and Blood Institute, National Institutes of Health (NIH), Bethesda, Maryland, USA.; 6 Department of Chemistry, Johns Hopkins University, Baltimore, Maryland, USA.; 7 Department of Plastic & Reconstructive Surgery, Johns Hopkins University School of Medicine, Baltimore, Maryland, USA.; 8 Department of Biochemistry, Emory University School of Medicine, Atlanta, Georgia, USA.; 9 Program in Molecular Biophysics, Johns Hopkins University School of Medicine, Baltimore, Maryland, USA.; 10 Department of Biochemistry, Faculty of Science, Charles University, Prague, Czech Republic.; 11 Laboratory of Tumor Immunology, Institute of Microbiology of the Academy of Sciences of the Czech Republic, Prague, Czech Republic.; 12 Vascularized Composite Allotransplantation Laboratory, Department of Plastic and Reconstructive Surgery;; 13 Translational Tissue Engineering Center;; 14 Department of Oncology;; 15 Bloomberg-Kimmel Institute for Cancer Immunotherapy;; 16 Sidney Kimmel Comprehensive Cancer Center; and; 17 Department of Ophthalmology, Johns Hopkins University School of Medicine, Baltimore, Maryland, USA.

**Keywords:** Immunology, Therapeutics, Cancer immunotherapy, Cytokines, Drug therapy

## Abstract

Progress in cytokine engineering is driving therapeutic translation by overcoming these proteins’ limitations as drugs. The IL-2 cytokine is a promising immune stimulant for cancer treatment but is limited by its concurrent activation of both pro-inflammatory immune effector cells and antiinflammatory regulatory T cells, toxicity at high doses, and short serum half-life. One approach to improve the selectivity, safety, and longevity of IL-2 is complexing with anti–IL-2 antibodies that bias the cytokine toward immune effector cell activation. Although this strategy shows potential in preclinical models, clinical translation of a cytokine/antibody complex is complicated by challenges in formulating a multiprotein drug and concerns regarding complex stability. Here, we introduced a versatile approach to designing intramolecularly assembled single-agent fusion proteins (immunocytokines, ICs) comprising IL-2 and a biasing anti–IL-2 antibody that directs the cytokine toward immune effector cells. We optimized IC construction and engineered the cytokine/antibody affinity to improve immune bias. We demonstrated that our IC preferentially activates and expands immune effector cells, leading to superior antitumor activity compared with natural IL-2, both alone and combined with immune checkpoint inhibitors. Moreover, therapeutic efficacy was observed without inducing toxicity. This work presents a roadmap for the design and translation of cytokine/antibody fusion proteins.

## Abstract 2

We engineered a cytokine/antibody fusion protein that specifically directs signaling of the interleukin-2 towards immune effector cells, leading to safe, targeted, and effective antitumor activity.

## Introduction

IL-2 is a multifunctional cytokine produced primarily by T cells and coordinating numerous essential activities in immune cells. IL-2 is vital for inducing proliferation of both pro-inflammatory immune effector cells (Effs, i.e., CD4^+^ and CD8^+^ effector T cells and NK cells) and antiinflammatory regulatory T cells (T_regs_) (1, 2). These activities make IL-2 an alluring candidate for therapeutic immunomodulation in diseases ranging from cancer to autoimmune disorders. Unfortunately, stimulation of both pro- and antiinflammatory cells has limited the cytokine’s efficacy in the treatment of cancer, and for the 5%–10% of patients whose cancer does respond to therapy, high doses of IL-2 are required for sustained tumor regression (3). High-dose IL-2 is frequently accompanied by toxicities (most prominently vascular leak syndrome) that can sometimes be fatal (4), limiting the cytokine’s clinical application. Moreover, IL-2 also has an extremely short serum half-life (<5 minutes), further complicating therapeutic use (5).

Fusing IL-2 to a tumor-targeting antibody to form an “immunocytokine” (IC) is a drug development approach that has been adopted to mitigate IL-2 toxicity (6). These molecules promote a spatially targeted immune response while increasing serum half-life via neonatal Fc receptor–mediated (FcRn-mediated) recycling (7), thereby attenuating the systemic toxicity observed for IL-2. Other trials have improved therapeutic efficacy by combining IL-2 with another therapeutic agent, such as traditional chemotherapeutics (8) or immune checkpoint inhibitors (ICIs) (9–11).

In addition to combining IL-2 with other treatments, researchers have disrupted or biased the interaction between IL-2 and its cognate receptor to achieve a greater therapeutic effect at lower, less toxic doses. These strategies have shown potential to selectively stimulate particular immune cell subsets for targeted disease therapy. IL-2 signals through IL-2 receptor-β (IL-2Rβ) and common gamma (γ_C_) chains on both immunostimulatory Effs and immunosuppressive T_regs_ (1, 12). IL-2–mediated heterodimerization of IL-2Rβ and γ_C_ activates the JAK/STAT pathway, inducing phosphorylation of STAT5 to activate gene expression programs that dictate cellular behavior (13). IL-2 can alternatively form a heterotrimeric receptor, consisting of IL-2Rα, IL-2Rβ, and γ_C_, which has 100-fold higher affinity than the IL-2Rβ/γ_C_ heterodimeric receptor (12). Thus, cells that express IL-2Rα (i.e., T_regs_) have far greater responsiveness to IL-2 compared with those that do not (i.e., naive Effs) (1, 14).

Disrupting interaction between IL-2 and IL-2Rα eliminates the competitive advantage for IL-2Rα–expressing cells, enabling more potent stimulation of pro-inflammatory Effs. This approach has been employed to enhance IL-2’s activity as an anticancer agent. One such strategy involves the design of mutant IL-2 variants (termed muteins) that have reduced or fully abrogated interaction with IL-2Rα (15–28). Other IL-2 biasing strategies include conjugation to polyethylene glycol (PEG) (29–32) and fusion to the IL-2Rα receptor (33, 34). However, these approaches have been limited by short half-life, pleiotropic effects, and formulation challenges. Furthermore, many of these molecules comprise mutated versions of IL-2, which can destabilize the cytokine and potentially induce neutralizing antidrug antibodies that crossreact with endogenous protein. Moreover, recent studies have demonstrated that mutated versions of IL-2, which do not engage IL-2Rα, fail to synergize with ICIs, since they eliminate the positive transcriptional feedback loop on effector T cells mediated by IL-2Rα upregulation following IL-2–induced activation (28, 35). As an alternative IL-2 biasing approach, monoclonal antibodies that target the cytokine and block the IL-2Rα–binding site were identified (36). Complexing IL-2 with these antibodies induced preferential stimulation of Effs compared with IL-2 alone while extending serum half-life, leading to improvements in antitumor efficacy for both mouse IL-2 (mIL-2), using monoclonal antibody S4B6 (36–40), and for human IL-2 (hIL-2), using monoclonal antibodies MAB602 (37, 38), NARA1 (41, 42), and TCB2 (22). Unfortunately, clinical translation of a cytokine/antibody complex is hindered by logistical hurdles, such as dosing ratio optimization, along with concerns regarding complex stability, as dissociation would lead to toxicities and off-target effects from the free cytokine and eliminate serum half-life extension.

This study blended the IC and cytokine-targeted antibody approaches by designing a single-agent fusion protein comprising hIL-2 and the anti–hIL-2 antibody MAB602 (referred to henceforth as 602), which acts as an intramolecularly assembled IC. Tethering IL-2 to the antibody stabilizes the cytokine/antibody complex, reducing off-target activation, and this tethering alone dramatically improved biased stimulation of Effs. Moreover, fusion of IL-2 to the antibody improves the pharmacokinetics of the therapy by preventing cytokine clearance through covalent linkage to the antibody. Furthermore, as a single-agent therapeutic, our IC eliminates questions of IL-2/antibody complex formulation and stoichiometric optimization, mitigating regulatory obstacles to translation. We applied molecular evolution approaches to isolate a variant of 602 that enhanced biased stimulation and expansion of Effs over T_regs_, leading to robust inhibition of tumor growth across multiple mouse cancer models. Importantly, we showed that durable antitumor effects were achieved in the absence of toxicities typically associated with IL-2 treatment, such as weight loss, pulmonary edema, inflammatory cytokine secretion, and liver damage. Together, this work constitutes a major step forward in advancing IL-2 biasing antibodies as viable candidates for clinical translation.

## Results

### Modifying linker length optimizes 602 IC production.

**Figure 1:**
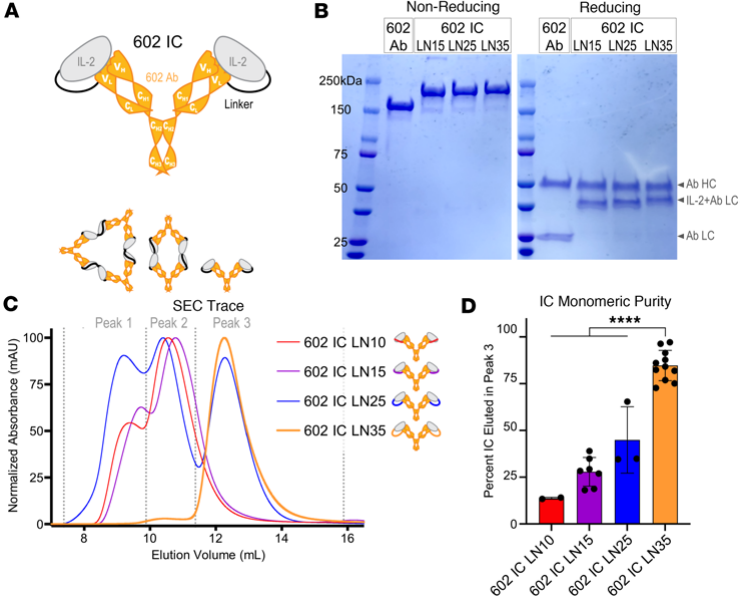
Optimization of IC design. (**A**) Schematic of the IL-2 cytokine/602 antibody (Ab) single-chain fusion protein (IC), wherein the C-terminus of the cytokine is tethered to the N-terminus of the Ab light chain (LC) via a flexible linker. Heavy chain (HC) and LC variable and constant domains are labeled. (**B**) 602 Ab and ICs migrate as expected by SDS-PAGE, under nonreducing and reducing conditions. (**C**) Representative SEC traces show the relative distribution of 4 linker length variants of 602 among 3 peaks, corresponding to either multi-IC oligomers (peaks 1 and 2) or monomeric IC (peak 3). (**D**) Average percentage of each IC that eluted in peak 3 based on area under curve. Data represent mean ± SD from 2–11 purifications. Significance is shown only for comparisons with 602 IC LN35. ****P < 0.0001 by 1-way ANOVA with Tukey’s test.

To combine the potency of cytokines with the pharmaceutically favorable properties of antibodies, IL-2 was fused to the IL-2Rα–competitive anti–IL-2 antibody 602 (Figure 1A) (37, 38) to create an intramolecularly assembled IC. The C-terminus of IL-2 was tethered to the N-terminus of the 602 antibody LC by a (G_4_S)_n_ linker of length 10, 15, 25, or 35 amino acids (denoted 602 IC LN10, 602 IC LN15, 602 IC LN25, or 602 IC LN35, respectively). All 602 ICs migrated at the expected molecular weights via SDS-PAGE (Figure 1B); however, size-exclusion chromatography (SEC) traces revealed distinctive elution profiles, with each IC partitioning between 3 peaks (Figure 1C). The first 2 peaks corresponded to proteins with much larger molecular weights than a single 602 IC, likely containing oligomers of multiple ICs that have exchanged one or both of their IL-2 moieties with another IC rather than binding intramolecularly to the 602 antibody to which they are tethered (Figure 1C). The first 2 peaks are dominant in 602 IC LN10 and 602 IC LN15, which is likely due to the linker being of inadequate length to accommodate intramolecular cytokine/antibody binding. Indeed, increasing the length of the linker to 25 amino acids introduced a third peak corresponding to the expected molecular weight for monomeric 602 IC (Figure 1C and Supplemental Figure 1A; supplemental material available online with this article; https://doi.org/10.1172/jci.insight.173469DS1), though more than half of the 602 IC LN25 eluted in the first 2 peaks (Figure 1, C and D, and Supplemental Figure 1, B and C). Fractions collected from all 3 SEC peaks of 602 IC LN25 migrated identically via nonreducing SDS-PAGE (Supplemental Figure 1D), indicating that assembly of higher-order oligomers was reversible. Extending the linker to 35 amino acids resulted in a molecule that eluted predominantly (~85%) in the third peak (Figure 1, C and D). Over multiple preps of 602 IC LN35, a greater percentage of the protein eluted in the third peak rather than the second when the protein was less concentrated (<5 μM) prior to SEC separation (Supplemental Figure 1E), but even at concentrations greater than 20 μM, over 75% of 602 IC LN35 eluted in the third peak. Together, these results demonstrate that extending linker length enhanced intramolecular assembly, enabling purification of monomeric IC.

### Extended intramolecular linker optimizes 602 IC function.

To assess the function of species contained in each SEC peak, the peaks of 602 IC LN25 were separately pooled and concentrated (Supplemental Figure 1, A and D). Interestingly, the contents of all 3 peaks exhibited identical binding to IL-2 and IL-2Rβ, as assessed by biolayer interferometry (BLI) (Supplemental Figure 1, F and G). Signaling activity of the eluted peaks was compared by measuring phosphorylation of STAT5 as a readout for IL-2 signaling on human YT-1 NK cells. In contrast with the observed binding behavior, we found that the contents of the first 2 peaks elicited weaker signaling compared with those of the third peak (Supplemental Figure 1H). For this reason, all subsequent comparisons used the third peak of the 602 IC LN25 and LN35 ICs, corresponding to the size of monomeric IC. As there was no distinct third peak for LN15, the second peak was used in subsequent comparisons.

**Figure 2:**
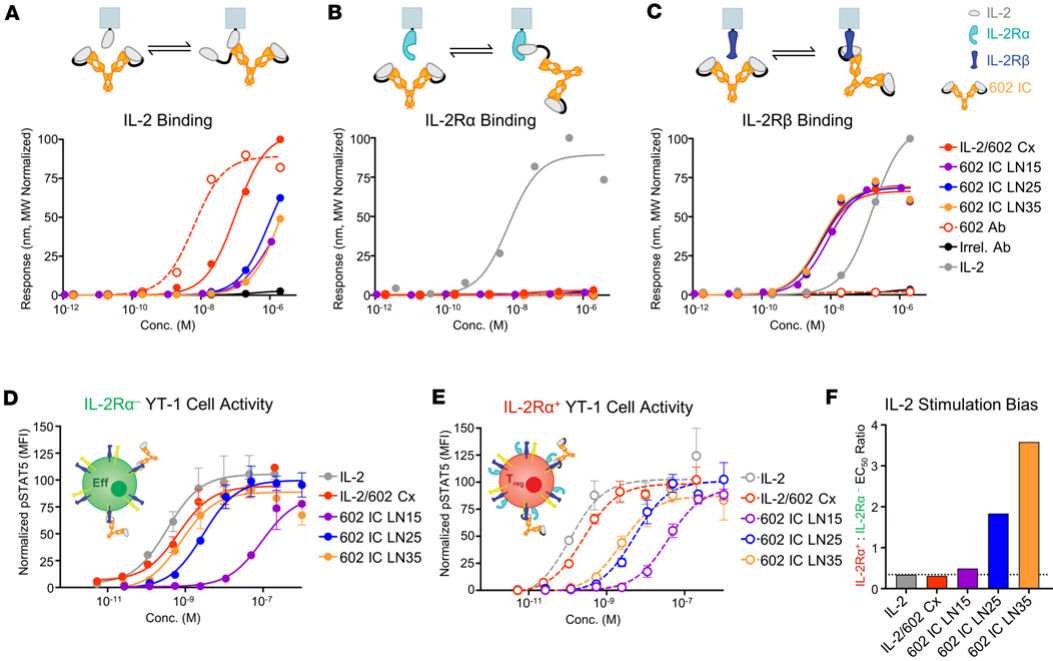
Increasing the linker length within ICs improves stability and enhances Eff bias. (**A**–**C**) Equilibrium BLI titrations of soluble IL-2/602 complex (Cx, 2:1 molar ratio), 602 Ab, and ICs against immobilized IL-2 (**A**), IL-2Rα (**B**), or IL-2Rβ (**C**). An antibody with irrelevant specificity (Irrel. Ab) served as a negative control. (**D** and **E**) STAT5 phosphorylation response of IL-2Rα^–^ (**D**) or IL-2Rα^+^ (**E**) YT-1 human NK cells treated with IL-2, IL-2/602 Cx (1:1 molar ratio), 602 Ab, or ICs. Data represent mean ± SD (*n* = 3). (**F**) Ratio of STAT5 phosphorylation EC_50_ values for treated IL-2Rα^+^ to IL-2Rα^–^ cells.

To assess functional differences between 602 ICs with various linker lengths, binding interactions with IL-2, IL-2Rα, and IL2Rβ were analyzed by BLI. All 3 602 ICs showed similar equilibrium binding properties (Figure 2, A–C; Supplemental Figure 2, A–C; and Supplemental Table 1). Tethering IL-2 to 602 in the ICs reduced exchange with immobilized IL-2 as compared with the IL-2/602 mixed cytokine/antibody complex, principally through association rate deceleration, illustrating the increased complex stability resulting from cytokine/antibody tethering. 602 IC LN35 showed a very similar profile to IL-2/602 complex by analytical ultracentrifugation, which revealed small amounts of dimeric and trimeric species for the IC and potential dimeric species for IL-2/602 complex that may have resulted from low levels of dimeric IL-2 (Supplemental Figure 3A). Thermostability measurements conducted by differential scanning fluorometry (DSF) revealed that the melting temperature (T_m_) of 602 IC LN35 was at least 0.5°C higher than that of IL-2/602 complex (Supplemental Figure 3, B and C, and Supplemental Table 2), further illustrating the increase in overall molecular stability for the IC as compared with the complex. As expected based on the competitive properties of the 602 antibody (37, 38), none of the 602 ICs engaged IL-2Rα (Figure 2B and Supplemental Figure 2B). The IL-2/IL-2Rβ interaction had an equilibrium dissociation constant of 112 nM (Figure 2C), similar to literature reports (17, 35, 39, 43–46). IL-2/602 complex and ICs showed identical equilibrium binding to IL-2Rβ (Figure 2C), indicating that the linkers did not interfere with the IL-2/IL-2Rβ interaction, and binding was potentiated compared with the free cytokine because of bivalency. Notably, 602 ICs with shorter linkers had slower IL-2Rβ association and dissociation rates compared with 602 IC LN35 and IL-2/602 complex (Supplemental Figure 2C and Supplemental Table 1), possibly due to the presence of oligomeric species.

To assess biased IL-2 signaling of 602 ICs, we compared their relative activation of unmodified, IL-2Rα^–^ YT-1 cells versus induced, IL-2Rα^+^ YT-1 cells (47), as surrogates for Effs versus T_regs_, respectively. Signaling activity of 602 IC LN15 on IL-2Rα^–^ (Eff-like) cells was markedly diminished compared with that of IL-2/602 complex (Figure 2D and Supplemental Table 3). Interestingly, although IL-2/602 complex induces strong in vivo bias toward activation of Effs over T_regs_ (37, 38), the complex behaved similarly to native IL-2 and was biased toward activation of IL-2Rα^+^ YT-1 cells in these in vitro studies, most likely due to antibody dissociation. In contrast, functional ICs reversed this stimulation bias. Activity improved when the linker length was increased to 25, and IL-2 signaling was fully restored for 602 IC LN35. On IL-2Rα^+^ (T_reg_-like) cells, signaling of 602 IC LN35 was only partially restored compared with that of IL-2/602 complex (Figure 2E), which is likely a result of increased blockade of the IL-2/IL-2Rα interaction due to enhanced stability of the cytokine/antibody interaction within ICs. Eff-biased IL-2 activity was quantified as the EC_50_ ratio of STAT5 phosphorylation on IL-2Rα^+^ T_reg_-like cells versus IL-2Rα^–^ Eff-like cells (Figure 2F). All 3 of the 602 ICs improved bias toward Effs compared with IL-2 alone, and 602 IC LN35 showed the most dramatic improvement in bias, reversing the EC_50_ ratio from favoring T_regs_ by a 3:1 margin to favoring Effs by a 3.6:1 margin. Based on binding and activity studies, we identified 35 amino acids as the optimal linker length, and 602 IC LN35 (denoted 602 IC henceforth) was used in all subsequent studies.

### Engineered 602 IC enhances disruption of IL-2/IL-2Rα interaction.

**Figure 3:**
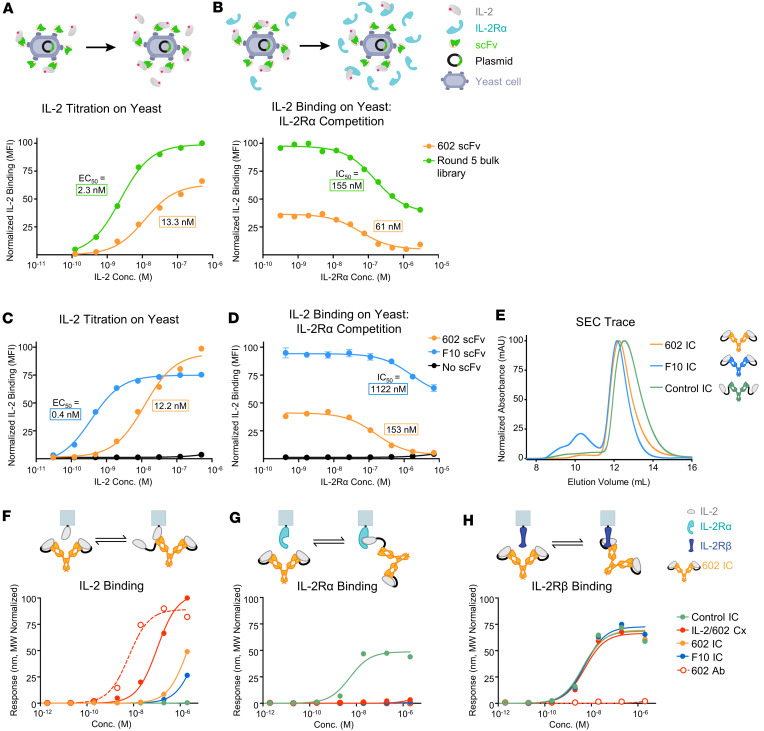
Engineered 602 variants exhibit stronger competition with IL-2Rα compared with the parent antibody. (**A** and **B**) IL-2 binding to yeast-displayed 602 scFv compared with evolved library of 602 scFv variants following 5 rounds of selection. IL-2 titrations (**A**) and IL-2 binding (10 nM) in the presence of various concentrations of IL-2Rα competitor (**B**) are shown. (**C** and **D**) IL-2 binding to yeast-displayed 602 scFv compared with the 602 scFv variant F10. IL-2 titrations (**C**) and IL-2 binding (5 nM) in the presence of various concentrations of IL-2Rα competitor (**D**) are shown. Yeast cells transformed with a plasmid lacking an scFv were used as a negative control. Data represent mean ± SD (*n* = 3). (**E**) SEC traces for ICs. (**F**–**H**) Equilibrium BLI titrations of soluble IL-2/602 Cx (2:1 molar ratio), 602 Ab, and ICs against immobilized IL-2 (**F**), immobilized IL-2Rα (**G**), and immobilized IL-2Rβ (**H**).

To further bias 602 IC activity toward Effs over T_regs_, we generated an error-prone mutagenic DNA library that randomized the first and third complementarity-determining regions (CDRs) of the variable HC and LC of the 602 antibody, expressed in single-chain variable fragment (scFv) format. The library was transformed into competent yeast and evolved against hIL-2 using the yeast surface display directed evolution platform (48) through iterative rounds of magnetic-activated cell sorting and fluorescence-activated cell sorting. Later rounds of selection were performed in the presence of excess IL-2Rα to identify clones that outcompeted soluble receptor for IL-2 engagement. After 5 sorting rounds, the evolved library showed improved binding to IL-2 (Figure 3A) and enhanced competition with the IL-2Rα receptor subunit (Figure 3B). Clones from the evolved library that showed superior competition with IL-2Rα for IL-2 binding compared with the parent 602 scFv were selected for characterization (Supplemental Figure 3, D and E). Among the sequenced clones, no mutations were observed in the HC CDR1 (CDR1H), only 1 variant contained a mutation in the CDR1L, and mutations in the CDR3H were restricted to a single residue. Most mutations were localized to CDR3L, and consensus was observed in mutation of F225 (F91 in CDR3L, Kabat numbering) from phenylalanine to less bulky amino acids.

Selected 602 scFv variants were produced recombinantly in IC LN35 format (Supplemental Figure 3F). Compared with the parent 602 IC, IC variants exhibited greater sensitivity to concentration. In particular, the percentage of protein eluted in the third peak for representative variant F10 (Figure 3, C and D) declined monotonically with increasing concentration at SEC column injection (Supplemental Figure 3G), whereas the percentage of protein eluted in the third peak for 602 IC plateaued at concentrations greater than 5 μM (Supplemental Figure 1E). As an experimental control, we prepared an IC construct in LN35 format, denoted control IC, containing IL-2 fused to an irrelevant antibody: the antifluorescein antibody 4-4-20 (49), which shares the mouse IgG2a κ isotype with 602. A smaller fraction of control IC eluted in peaks 1 and 2 compared with 602 IC and variant F10 IC, presumably because of the absence of oligomeric complexes consistent with the lack of cytokine/antibody interaction (Figure 3E and Supplemental Figure 3, H–J). These observations aligned with analytical ultracentrifugation analysis, which showed the presence of small amounts of dimeric and trimeric species for 602 IC and F10 IC but only monomeric species for control IC (Supplemental Figure 3L). All 602 IC variants and control IC migrated as expected via SDS-PAGE (Supplemental Figure 3K).

To characterize the biophysical behavior of engineered 602 IC variants, BLI was used to assess binding to immobilized IL-2, IL-2Rα, and IL2Rβ. The 602 IC variants A8 IC, C5 IC, and F10 IC showed weaker interaction with immobilized IL-2 compared with the parent IC (Figure 3F, Supplemental Figure 4A, Supplemental Figure 5A, and Supplemental Table 4), indicative of more stable intramolecular interactions for these variants because of stronger cytokine/antibody affinity. Consistent with yeast surface competitive binding experiments (Supplemental Figure 3E), 602 IC variants, like the parent IC, did not interact with immobilized IL-2Rα (Figure 3G, Supplemental Figure 4B, and Supplemental Figure 5B). All 602 IC variants bound IL-2Rβ with similar affinity compared to the parent 602 IC and control IC (Figure 3H, Supplemental Figure 4C, and Supplemental Figure 5C).

Variant F10 was further analyzed and showed higher affinity for IL-2 as an scFv compared with 602 scFv (Supplemental Figure 6, A and C, and Supplemental Table 5), largely due to a 3.6-fold reduction in dissociation rate. The more pronounced differences for the 602 versus F10 scFvs compared with the respective ICs is likely due to both avidity contributions and the apparent increase in affinity resulting from cytokine/antibody tethering. Consistent with this, DSF thermostability measurements revealed that the T_m_ for F10 IC was 1.4°C higher than that for 602 IC, and the T_m_ 602 IC was 0.6°C higher than that for IL-2/602 complex (Supplemental Figure 3, M and N, and Supplemental Table 2). Notably, IL-2/602 complex (at a stoichiometrically equivalent 2:1 cytokine/antibody ratio) bound to immobilized IL-2 with only slightly reduced affinity compared with the unbound 602 antibody (Figure 3F, Supplemental Figure 4A, and Supplemental Figure 5A), whereas IL-2/F10 complex and F10 IC showed minimal binding to immobilized IL-2 (Supplemental Figure 6, B and D). Moreover, F10 IC exhibited weaker binding to immobilized IL-2 compared with 602 IC, further highlighting the increased stability of IL-2 binding for F10 versus 602. As expected, control IC did not interact with immobilized IL-2 (Figure 3F, Supplemental Figure 2A, Supplemental Figure 4A, and Supplemental Figure 5A). Kinetic data revealed that both 602 IC and F10 IC blocked IL-2/IL-2Rα interaction more effectively than IL-2/602 complex (Supplemental Figure 5B and Supplemental Table 1). Control IC bound IL-2Rα with increased affinity compared with IL-2 because of bivalency (Figure 2B, Figure 3G, and Supplemental Table 1). Notably, 602 IC, F10 IC, and IL-2/602 complex had slower IL-2Rβ association rates compared with control IC, likely due to steric effects resulting from intramolecular assembly (Figure 2B, Figure 3G, Supplemental Figure 2C, and Supplemental Table 1). Nonetheless, the IL-2/IL-2Rβ interaction remained intact for all ICs, allowing for selective disruption of the IL-2/IL-2Rα interaction.

In anticipation of in vivo experiments in mice, we assessed binding of F10 IC to mIL-2 receptor subunits (Supplemental Figure 6, E–H, and Supplemental Table 1). As with the respective human receptor, F10 IC did not interact with immobilized mIL-2Rα (Figure 3G; Supplemental Figure 2B; Supplemental Figure 6, E and G; and Supplemental Table 1). Further, F10 IC bound to mIL-2Rβ, albeit weaker than its interaction with hIL-2Rβ because of the weaker interaction between hIL-2 with mIL-2Rβ versus hIL-2Rβ (39) (Figure 3H; Supplemental Figure 2C; Supplemental Figure 6, F and H; and Supplemental Table 1). Control IC showed more profound reduction in binding to mIL-2Rβ versus hIL-2Rβ compared with F10 IC, suggesting that the F10 engagement of IL-2 enhances the IL-2/IL-2Rβ interaction (Figure 3H; Supplemental Figure 2C; Supplemental Figure 6, F and H; and Supplemental Table 1). Collectively, binding and thermostability studies verified our evolution of 602 IC variants with enhanced stability of intramolecular cytokine/antibody assembly.

### Engineered 602 IC shows enhanced IL-2 bias toward Effs.

**Figure 4:**
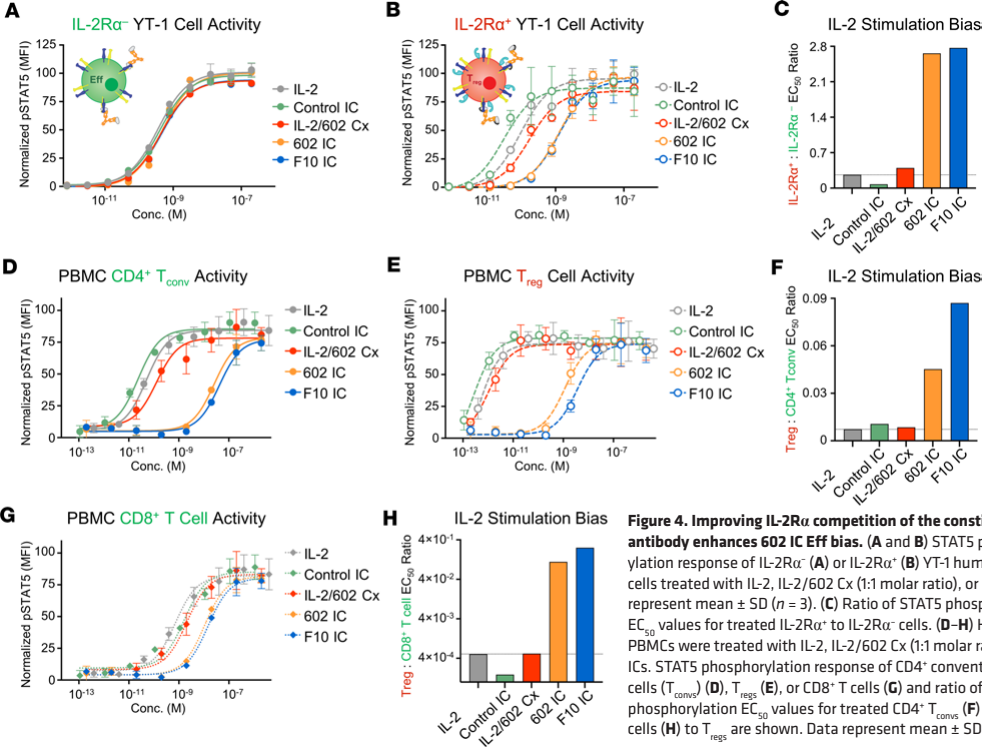
Improving IL-2Rα competition of the constituent antibody enhances 602 IC Eff bias. (**A** and **B**) STAT5 phosphorylation response of IL-2Rα^–^ (**A**) or IL-2Rα^+^ (**B**) YT-1 human NK cells treated with IL-2, IL-2/602 Cx (1:1 molar ratio), or ICs. Data represent mean ± SD (*n* = 3). (**C**) Ratio of STAT5 phosphorylation EC_50_ values for treated IL-2Rα^+^ to IL-2Rα^–^ cells. (**D**–**H**) Human PBMCs were treated with IL-2, IL-2/602 Cx (1:1 molar ratio), and ICs. STAT5 phosphorylation response of CD4^+^ conventional T cells (T_convs_) (**D**), T_regs_ (**E**), or CD8^+^ T cells (**G**) and ratio of STAT5 phosphorylation EC_50_ values for treated CD4^+^ T_convs_ (**F**) or CD8^+^ T cells (**H**) to T_regs_ are shown. Data represent mean ± SD (*n* = 3).

We hypothesized that preferential engagement of IL-2Rβ over IL-2Rα by our biased ICs would decrease the natural bias of IL-2 toward IL-2Rα^+^ (T_reg_-like) cells, thereby favoring activation of IL-2Rα^–^ (Eff-like) cells. Stimulation of a mixed population of IL-2Rα^–^ and IL-2Rα^+^ YT-1 cells revealed that 3 of 5 engineered 602 IC variants showed stronger IL-2Rα^–^ cell bias compared with the parent 602 IC (Supplemental Figure 4, D–F). Among the variants, F10 IC (which contained the mutations T101S, F225S, and G227D [T99S in CDR3H and F91S and G93D in CDR3L, Kabat numbering]) was most skewed toward IL-2Rα^–^ cell stimulation, flipping the natural bias of IL-2 to favor signaling on IL-2Rα^–^ Eff-like cells over IL-2Rα^+^ T_reg_-like cells (Figure 4, A–C, and Supplemental Table 6); thus, this molecule was selected for further characterization. Notably, whereas 602 IC and F10 IC exhibited nearly identical potency on IL-2Rα^–^ YT-1 cells, F10 IC was 7.65-fold weaker than 602 IC on IL-2Rα^+^ YT-1 cells. Like IL-2, control IC and IL-2/602 complex were biased toward IL-2Rα^+^ over IL-2Rα^–^ cells. Similar biases were manifested on freshly isolated human PBMCs, and a marked advantage was observed for F10 IC compared with 602 IC (Figure 4, D–H, and Supplemental Table 6). Specifically, signaling of 602 IC and F10 IC was substantially more impaired on T_regs_ compared with CD4^+^ T_convs_ and CD8^+^ T cells, whereas control IC and IL-2/602 complex showed similar bias to IL-2 alone or further biased the cytokine toward T_reg_ activation. Moreover, F10 IC showed 1,064-fold weaker potency than 602 IC on T_regs_ (Supplemental Table 6). Consequently, activation ratios for both CD4^+^ T_convs_ and CD8^+^ T cells relative to T_regs_ were higher for F10 IC versus the parent 602 IC (Figure 4, F and H), highlighting the benefit of our IL-2 biasing efforts, particularly in the context of a mixed immune cell environment. Together, signaling data demonstrated that increasing its IL-2Rα competition and IL-2 affinity enhanced the bias of 602 IC toward Effs over T_regs_.

### Structural basis for 602-mediated disruption of IL-2/IL-2Rα interaction.

**Figure 5:**
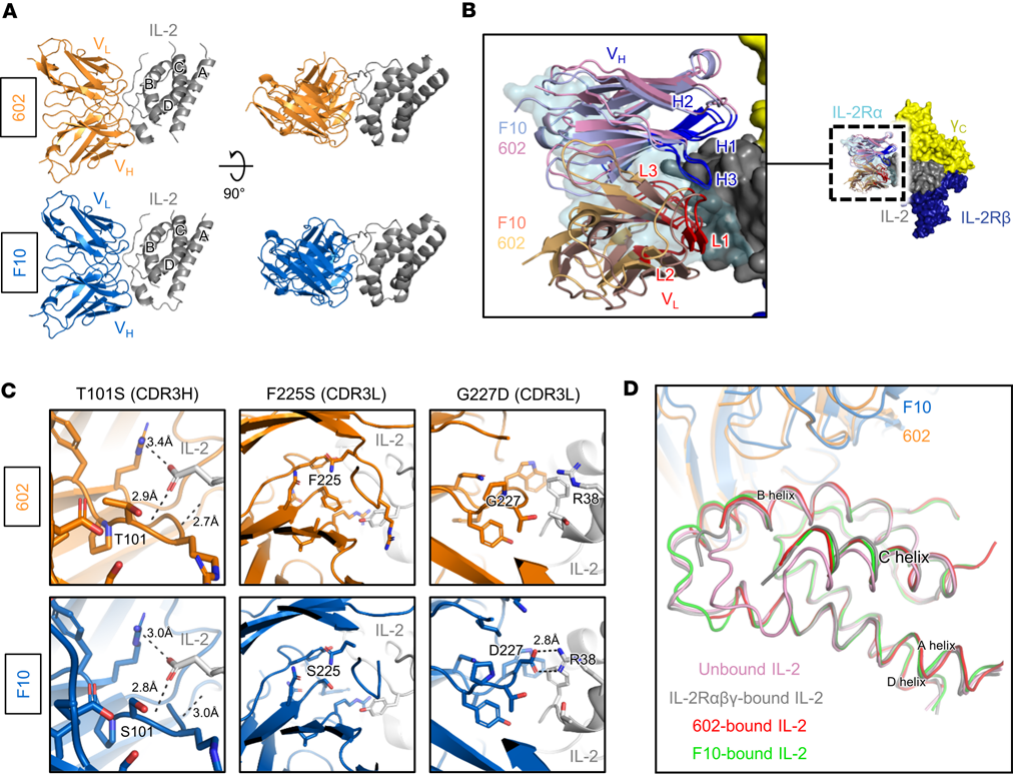
Structural basis for biased activity of F10 IC. (**A**) Crystallographic structure of the IL-2/F10 scFv (Protein Data Bank [PDB] 8SOW) and IL-2/602 scFv (PDB 8SOZ) complexes. (**B**) Overlay of the IL-2/F10 scFv complex and IL-2 cytokine/receptor complex (PDB 2B5I) structures. HC and LC CDRs are delineated. (**C**) Detailed view of differential IL-2 interactions for F10 versus the parent 602 scFv. Mutations T101S, F225S, and G227D are shown, depicting all side chains (sticks) and interchain polar contacts (black dashed lines) within 5 Å of the mutated residues. (**D**) Overlay of IL-2 in the unbound (pink, PDB 3INK), IL-2Rαβγ–bound (gray, PDB 2B5I), 602 scFv–bound (red), and F10 scFv–bound (green) states.

To further characterize the mechanistic behavior of the 602 antibody and engineered variants thereof, we determined the molecular structure of the IL-2/602 scFv complex. Crystals diffracted to 1.65 Å, and the structure was phased via molecular replacement (Figure 5A and Supplemental Table 7, Protein Data Bank [PDB] 8SOZ). 602 engagement of IL-2 occludes approximately 740 Å^2^ of area on the cytokine surface. On the cytokine side, the binding interface is primarily composed of residues in the AB loop (residues 38–45), the B helix (residues 62–69), and the CD loop and N-terminal end of the D helix (residues 107–116). On the 602 side, CDR3H and CDR3L both contribute substantially to IL-2 interaction (Figure 5B); notable features include hydrogen bonds by neighboring residues R100 and T101 and a stretch of interacting residues in CDR3L (S225, W226, D227) (Figure 5C). CDR1L also contributes to the IL-2/602 interface, including salt bridges formed by residues D162 and R166. To elucidate the rationale for enhanced IL-2 binding of F10 versus 602, we determined the 1.7 Å resolution crystal structure of the IL-2/F10 scFv complex (Figure 5A and Supplemental Table 7, PDB 8SOW). Interestingly, while 602 and F10 occlude an almost identical interface on the cytokine, they exhibit a subtle offset in binding angle (Figure 5, B and C). In addition, the IL-2–binding paratope on F10 is nearly identical to that of the parent 602 antibody. Whereas the T101S mutation in CDR3H and the F225S mutation in CDR3L introduced for F10 did not appear to alter cytokine/antibody interactions, the G227D mutation in CDR3L created a new salt bridge with residue R38 of IL-2, consistent with the observed improvement in IL-2 binding (Figure 5C).

When comparing the structure of IL-2/antibody complexes to the structure of IL-2 bound to its high-affinity receptor complex (PDB 2B5I), we noted that 602 and F10 both had considerable overlap with the IL-2Rα subunit but did not overlap with the IL-2Rβ or γ_C_ subunits (Figure 5B and Supplemental Figure 7, A and B). This focused obstruction of IL-2/IL-2Rα engagement was reminiscent of that observed for other Eff-biasing antibodies, specifically the anti–mIL-2 antibody S4B6 (36–40, 50, 51) and the anti–hIL-2 antibody NARA1 (41, 42). The epitopes on IL-2 engaged by 602 and F10 are similar, and although F10 occupies less interface on the γ_C_-adjacent portion of IL-2 compared with 602, overlap with the IL-2Rα subunit epitope is identical for the 2 antibodies (Supplemental Figure 7B). The predicted S4B6 binding interface on hIL-2 is distinct from the 602/F10 binding interface on hIL-2, with S4B6 binding closer to the IL-2Rβ subunit and 602/F10 binding closer to the γ_C_ subunit. Nonetheless, overlaps between the S4B6 and IL-2Rα interfaces and the 602/F10 and IL-2Rα interfaces are strikingly similar. In contrast, the NARA1 interface is centered between IL-2Rβ and γ_C_, and its overlap with the IL-2Rα subunit interface is more focused on the lower portion of IL-2, when viewed from a top-down perspective.

Furthermore, 602- and F10-bound IL-2 were essentially superimposable with receptor-bound IL-2, recapitulating the 15° shift that the C helix of IL-2 undergoes upon receptor binding (Figure 5D) (12, 17, 39). As was previously observed for the S4B6 antibody, the 602 and F10 antibodies allosterically prime IL-2 for engagement of IL-2Rβ and γ_C_ by enforcing this C helix shift, which is similarly induced by IL-2Rα binding. In addition, whereas structural alignments of mIL-2–bound S4B6 suggest that it may sterically clash with hIL-2Rβ (39), 602 and the F10 variant do not show any steric clashes with IL-2Rβ due to the shift in binding topology away from the IL-2Rβ subunit relative to S4B6 (Supplemental Figure 7C).

Structural insights also illustrated the benefit for using linker lengths greater than 15 amino acids to achieve optimal assembly of monomeric 602 IC. The linear distance between the C-terminus of IL-2 and the N-terminus of the 602 variable LC in the resolved structure is 43.6 Å (Supplemental Figure 7D). Assuming that the flexible linker between the cytokine and antibody is fully extended (>3 Å per residue), a minimum length of 15 amino acids would be required to span this distance. However, the linker cannot stretch linearly, since it must weave around structural elements. Consequently, a linker length greater than 15 amino acids is necessary for proper folding and function, consistent with observations from SEC (Figure 1C) and signaling assays (Figure 2, D and E). Crystallographic data also validate our assumption that IL-2 binds the antibody in *cis* (i.e., each IL-2 moiety binds to its tethered variable LC within the IC structure). Aligning the variable domains of 602 in the IL-2/602 scFv structure with an isotype-matched mouse IgG2a κ antibody structure (PDB 1IGT) (52) revealed that the linear distance between the C-terminus of IL-2 and the N-terminus of the nontethered 602 variable LC within the IC structure is 174.1 Å (Supplemental Figure 7E). Again assuming that the linker connecting the cytokine and antibody is fully extended, a minimum length of 58 amino acids would be required to bridge this distance. Thus, our 35–amino acid linker only allows for *cis* interactions. It is possible that substantially longer linkers could lead to *trans* interactions, which could impair IL-2 function through steric hindrance. Overall, structural studies depict the antibody’s focused inhibition of the IL-2Rα interaction and linker requirements, while revealing the molecular basis for improved stability of F10 IC versus 602 IC.

### ICs elicit similar gene expression profiles compared to IL-2 cytokine.

**Figure 6:**
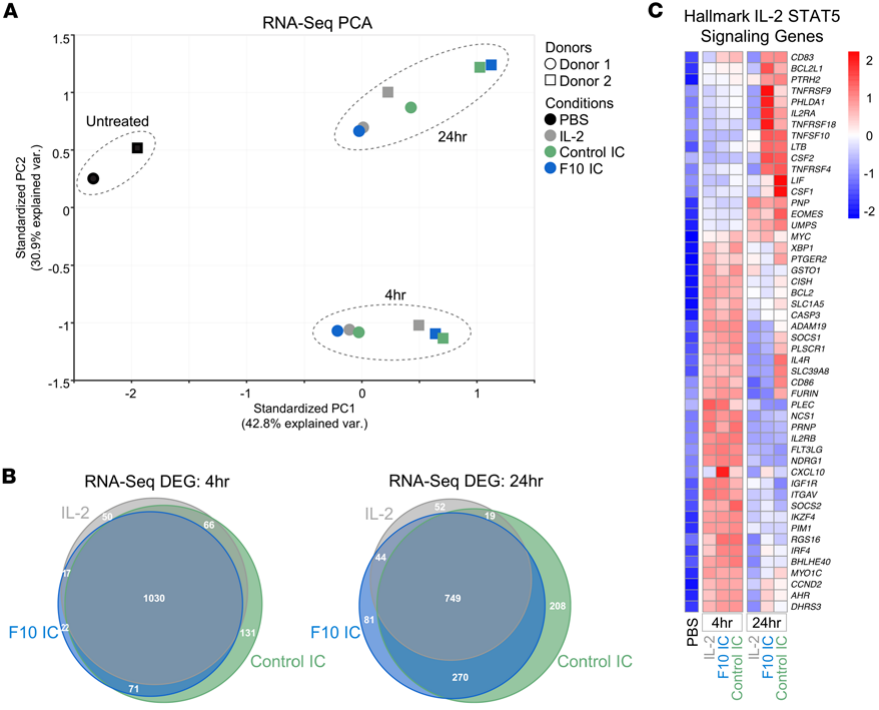
Unconjugated IL-2 and ICs induce similar gene expression profiles in human CD8^+^ T cells. (**A**) Principal components analysis visualization of RNA-Seq analysis performed on freshly isolated human CD8^+^ T cells from 2 independent donors stimulated with either PBS or saturating IL-2 (1 μM), F10 IC (0.5 μM), or control IC (0.5 μM) for either 4 or 24 hours. (**B**) Venn diagram depicting differentially expressed genes (DEGs) at 4 (left) and 24 (right) hours for each treatment compared with PBS-treated cells. (**C**) Heatmap representation of RNA-Seq analysis for hallmark STAT5 signaling genes. The color scale represents *z* score values.

To broadly examine whether fusing IL-2 to an antibody alters functional signaling compared with native IL-2, RNA-Seq analysis was performed on CD8^+^ T cells freshly isolated from human PBMCs that were treated with either PBS or a saturating dose of IL-2 (1 μM), F10 IC (0.5 μM), or control IC (0.5 μM) (Supplemental Figure 8A). Overall, the gene expression profiles for the 2 ICs were very similar to that of IL-2 (Figure 6, A and B). Some differences emerged after 24 hours of treatment, including differential expression of hallmark genes associated with STAT5 signaling, which showed higher induction in cells stimulated with F10 IC or control IC compared with IL-2 (Figure 6C). Differences in STAT5 hallmark genes, as well as some genes involved in T cell activation and apoptosis (Supplemental Figure 8, B and C), were likely due to differences in the potency of stimulation over time. The ICs may be less easily internalized relative to IL-2, or the bivalency of the ICs may induce stronger or more durable responses. Despite these slight deviations, the gene expression response to IC stimulation was remarkably similar to that seen with IL-2, indicating that antibody fusion does not substantially alter the quality of IL-2 signaling.

### Engineered 602 IC expands Eff subsets in vivo.

**Figure 7:**
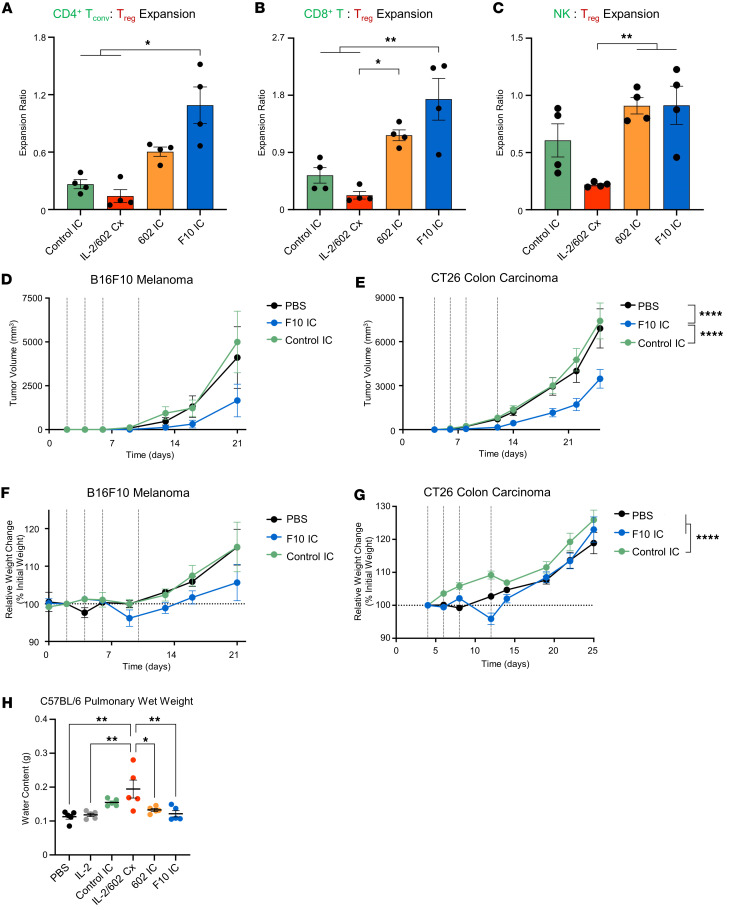
F10 IC promotes biased expansion of Effs and improves the therapeutic efficacy of IL-2. (**A**–**C**) C57BL/6 mice (*n* = 4) were injected intraperitoneally daily for 4 days with the molar equivalent of 0.075 mg/kg IL-2/dose of control IC, IL-2/602 Cx (2:1 molar ratio), 602 IC, or F10 IC. Spleens were harvested on day 5. Total counts of CD4^+^ T_convs_, CD8^+^ T cells, NK cells, and T_regs_ were determined by flow cytometry. Ratios of CD4^+^ T_convs_ to T_regs_ (**A**), CD8^+^ T cells to T_regs_ (**B**), and NK cells to T_regs_ (**C**) were calculated. (**D**–**G**) C57BL/6 mice (*n* = 7–9) were injected subcutaneously with B16F10 tumor cells (**D** and **F**), and BALB/c mice (*n* = 8) were injected subcutaneously with CT26 tumor cells (**E** and **G**). Mice were treated on days indicated with dashed lines with either PBS or the molar equivalent of 0.125 mg/kg IL-2 of control IC or F10 IC. Tumor volume (**D** and **E**) and percentage body weight changes relative to weight at the time of tumor implantation (**F** and **G**) are shown. (**H**) C57BL/6 mice (*n* = 5) were injected daily for 4 days with PBS, 0.075 mg/kg IL-2, or the molar equivalent of 0.075 mg/kg IL-2 of control IC, IL-2/602 Cx, 602 IC, or F10 IC. Day 5 lung water content was measured. Data are shown as mean ± SEM. *P < 0.05, **P < 0.01, ****P < 0.0001 by 2-way ANOVA with Tukey’s test. For tumor growth and mouse weight curves, significance is indicated for overall curves.

To determine whether F10 IC’s enhanced tropism toward Eff activation in vitro corresponded to increased expansion of Effs in vivo, we intraperitoneally administered control IC, IL-2/602 complex, 602 IC, or F10 IC and evaluated the abundance of immune cell subsets in harvested spleens. IL-2/602 complex elicited the greatest overall expansion of the immune cell subsets compared with PBS treatment (Supplemental Figure 9A). 602 IC and F10 IC elicited less expansion than IL-2/602 complex but induced greater expansion of CD8^+^ T cells, NK cells, memory phenotype CD8^+^ T cells (CD8^+^ T_MPs_), NKT cells, γδ T cells, and T_regs_ compared with PBS treatment. Only IL-2/602 complex and F10 IC expanded CD4^+^ T_convs_ above treatment with PBS, and control IC suppressed expansion. CD4^+^ T effector memory cells (CD4^+^ T_EMs_) were only expanded by IL-2/602 complex. Relative expansion ratios for Eff subsets versus T_regs_ were used to assess pro-inflammatory immune biasing (Figure 7, A–C, and Supplemental Figure 9, C and D). F10 IC preferentially expanded CD4^+^ T_convs_, CD8^+^ T cells, CD4^+^ T_EMs_, and CD8^+^ T_MPs_ relative to T_regs_, and effector bias was superior to that induced by control IC and IL-2/602 complex. F10 IC also preferentially expanded NK cells relative to T_regs_, showing greater effector bias than IL-2/602 complex and trending toward greater effector bias than control IC. 602 IC–induced effector bias trended lower than F10 IC–induced bias on all Eff subsets. Compared with 602 IC, F10 IC showed relative expansion improvements of 80%, 48%, 36%, and 26% on CD4^+^ T_convs_, CD8^+^ T, CD4^+^ T_EMs_, and CD8^+^ T_MPs_, relative to T_regs_, respectively.

Surprisingly, IL-2/602 complex showed less effector bias than control IC, due to significantly greater expansion of T_regs_ by IL-2/602 complex (Figure 7, A–C, and Supplemental Figure 9, A, C, and D). This could be due to recruitment of effector function through the antibody Fc domain. As IL-2 remains on the surface of T_regs_ when interacting with only the IL-2Rα subunit, and is only internalized upon binding to IL-2Rβ and γ_C_ (53), T_regs_ are likely to have more control IC on their surface compared with other immune cell subsets. Increased surface presentation of control IC could deplete T_regs_, thus attenuating their expansion relative to IL-2/602 complex treatment, in which case the antibody may freely dissociate. Consistent with this hypothesis, when Fc effector function was knocked out (referred to as ΔFc, LALA-PG Fc mutation; ref. 54) in control IC, T_reg_ expansion was significantly greater (Supplemental Figure 9, A and B). The only other significant difference observed between constructs containing native Fc versus ΔFc was in control IC–mediated expansion of CD8^+^ T_MPs_, which was also higher when effector function was knocked out. CD8^+^ T_MPs_ have substantially greater expression of IL-2Rβ that is not associated with higher levels of γ_C_ (22); thus, a lag in internalization may lead to Fc-mediated depletion. As no significant differences in F10 IC were observed upon effector function knockout, ICs with the native Fc were used for therapeutic studies. Collectively, these results demonstrate that F10 IC preferentially expands Effs over T_regs_ in vivo, creating an immunostimulatory environment that could be leveraged for cancer immunotherapy.

### Engineered 602 IC inhibits tumor growth without inducing toxicity and expands Eff subsets in the tumor microenvironment.

To assess the therapeutic potential of our IL-2 biasing strategy, we tested the performance of F10 IC in multiple syngeneic mouse tumor models. We first compared the antitumor activity of our F10 IC with that of IL-2 and IL-2/602 complex in a B16F10 syngeneic mouse melanoma model. In this model, IL-2/602 complex significantly inhibited tumor growth relative to PBS and IL-2, and F10 IC elicited even stronger suppression (Supplemental Figure 9E). We next compared the performance of F10 IC with control IC in the B16F10 model, to evaluate whether antitumor activity was driven by enhanced bias or by advantages imparted by the IC format. F10 trended toward tumor growth inhibition relative to control IC (*P* = 0.089) (Figure 7D). We further compared the ICs in a more immunogenic mouse tumor model, the CT26 model of colorectal carcinoma. F10 IC significantly suppressed tumor growth compared with both control IC and PBS (Figure 7E). In both tumor models, the body weights for mice treated with F10 IC and control IC were not significantly lower than those for mice treated with PBS (Figure 7, F and G). We noticed a slight reduction in body weight (<5%) for both models immediately following treatment with F10 IC (Figure 7, F and G); however, mice recovered after treatment termination. We also evaluated acute adverse effects of IC treatment in the form of pulmonary edema and liver injury. Mice were treated for 4 consecutive days with PBS, control IC, IL-2/602 complex, 602 IC, or F10 IC for 4 days and sacrificed on the fifth day to analyze pulmonary wet weight and serum concentrations of liver enzymes aspartate aminotransferase (AST) and alanine transaminase (ALT). IL-2/602 complex increased fluid content in the lungs of C57BL/6 but not BALB/c mice, and none of the ICs led to significant changes in pulmonary wet weight (Figure 7H and Supplemental Figure 9F). No significant differences in liver enzyme concentrations were observed between treatments (Supplemental Figure 9, G and H), indicating that F10 IC treatment did not lead to lung or liver toxicities.

To dissect the mechanistic effects of F10 IC in tumor models, we analyzed expansion of immune cell subsets in spleen (representing systemic effects) and in the tumor microenvironment (TME) of mice from the B16F10 and CT26 therapeutic models at the experimental endpoints, days 21 and 25, respectively. For the B16F10 model, as observed in non-tumor-bearing mice (Supplemental Figure 9, A and B), several Eff subsets were expanded by F10 IC relative to PBS and control IC, and effects were observed in the spleen (Supplemental Figure 10A) as well as the TME (Supplemental Figure 10B). We observed significantly increased levels of CD8^+^ T cells, NK cells, CD8^+^ T_MPs_, and NKT cells in the spleen of mice treated with F10 IC compared with control IC, and these same cell subsets trended higher for F10 IC relative to control IC in the TME. Conversely, CD4^+^ T_conv_ and CD4^+^ T_EM_ levels decreased following treatment with F10 IC compared with control IC but remained within 1.5-fold of the PBS cohort. Interestingly, whereas T_regs_ were expanded by control IC and, to a lesser extent, F10 IC in non-tumor-bearing mice relative to PBS treatment (Supplemental Figure 9, A and B), T_reg_ levels were not significantly changed in B16F10 tumor–bearing mice treated with ICs versus PBS in either the spleen or TME (Supplemental Figure 10, A and B). Relative expansion ratios for Effs versus T_regs_ revealed that F10 IC significantly enhanced the expansion ratios of CD8^+^ T cells, NK cells, CD8^+^ T_MPs_, and NKT cells compared with control IC in the spleen (Supplemental Figure 10C), and similar trends were observed in the TME (Supplemental Figure 10D). In contrast, splenic expansion ratios for both CD4^+^ T_convs_ and CD4^+^ T_EMs_ relative to T_regs_ were significantly reduced following F10 IC treatment compared with control IC treatment, although no significant differences were observed in the TME. In the spleen or TME, γδ T cells were not significantly expanded. Given that IL-2 activation can induce T cell exhaustion, we interrogated expression of the immune checkpoint protein programmed cell death protein 1 (PD-1) on CD8^+^ T cells and CD8^+^ T_MPs_ at the experimental endpoint. PD-1^+^ CD8^+^ T cell and CD8^+^ T_MP_ numbers significantly increased in the spleen of mice treated with F10 IC versus control IC (Supplemental Figure 10E), whereas PD-1^+^ CD8^+^ T cells and CD8^+^ T_MP_ numbers were not significantly changed in the TME following IC versus PBS treatment (Supplemental Figure 10F).

For the CT26 model, the results were generally more attenuated compared with the B16F10 model. Only CD8^+^ T_MPs_ were significantly expanded in the spleen following F10 IC but not control IC treatment (Supplemental Figure 11A), and none of the analyzed immune subsets were significantly expanded in the TME. NKT cells trended upward following F10 IC but not control IC treatment (Supplemental Figure 11B). No significant changes were observed in Eff versus T_reg_ expansion ratios between the ICs, except for CD4^+^ T_convs_ and NKT cells, which showed greater relative expansion in the spleen following control IC versus F10 IC treatment (Supplemental Figure 11C). In the TME, relative expansion ratios for CD8^+^ T cells, CD8^+^ T_MPs_, and NKT cells versus T_regs_ trended higher for F10 IC compared with control IC (Supplemental Figure 11D). As in the B16F10 model, PD-1^+^ CD8^+^ T cell and CD8^+^ T_MP_ numbers trended higher in the spleen of mice treated with F10 IC versus control IC (Supplemental Figure 11E), whereas PD-1^+^ CD8^+^ T cell and CD8^+^ T_MP_ numbers were not significantly changed in the TME following F10 IC versus PBS treatment (Supplemental Figure 11F).

We studied the acute effects of treatment with F10 IC compared with control IC and IL-2/602 complex in B16F10 tumor–bearing mice, performing a short-term study to characterize the immune response 24 hours after treatment. Overall, the immune profile in the spleen paralleled that observed at the endpoint (day 21) of the B16F10 therapeutic model, with significantly increased levels of CD8^+^ T cells, NK cells, CD8^+^ T_MPs_, and NKT cells for F10 IC versus control IC and IL-2/602 complex treatment (Supplemental Figure 12A). Splenic γδ T cell levels were also significantly increased following F10 IC versus IL-2/602 complex treatment. Splenic CD4^+^ T_convs_ were decreased following F10 IC versus control IC treatment but remained within 2-fold of the PBS cohort. Splenic CD4^+^ T_EMs_ showed no significant changes for treated versus PBS-treated mice. Splenic T_regs_ were increased following F10 IC treatment compared with control IC and IL-2/602 complex treatment, but changes were much less pronounced than those observed for CD8^+^ T cells, NK cells, CD8^+^ T_MPs_, and NKT cells. In the TME, results of the B16F10 short-term study paralleled those of the B16F10 therapeutic study, with significantly increased NK cell numbers and higher trending numbers of CD8^+^ T_MPs_ and NKT cells following F10 IC versus control IC treatment (Supplemental Figure 12B). However, CD8^+^ T cell numbers were not significantly changed in TME. CD4^+^ T_convs_, CD4^+^ T_EMs_, and γδ T cells were elevated in the TME following treatment with IL-2/602 complex, but not ICs, relative to PBS treatment. Importantly, in contrast with splenic T_regs_, TME T_regs_ were not expanded by F10 IC compared with control IC, whereas IL-2/602 complex expanded T_regs_ relative to control IC. Similar to the B16F10 therapeutic study, splenic Eff versus T_reg_ expansion ratios were significantly elevated for NK cells, CD8^+^ T_MPs_, and NKT cells and trended higher for CD8^+^ T cells following F10 IC compared with control IC and IL-2/602 complex treatment (Supplemental Figure 12C). Splenic expansion ratios for CD4^+^ T_convs_, CD4^+^ T_EMs_, and γδ T cells were significantly reduced following F10 IC versus control IC and IL-2/602 complex treatment. In contrast with the B16F10 therapeutic study, the TME expansion ratio for CD8^+^ T cells was significantly decreased, and TME expansion ratios for NK cells, CD8^+^ T_MPs_, and NKT cells were unchanged following F10 IC versus control IC treatment in the short-term study (Supplemental Figure 12D). IL-2/602 complex significantly decreased TME expansion ratios for CD8^+^ T cells and CD8^+^ T_MPs_ and led to lower trending TME expansion ratios for NK and NKT cells compared with control IC. Although no significant changes were observed in TME expansion ratios for CD4^+^ T_EMs_ and γδ T cells in the B16F10 therapeutic study, these ratios were both significantly reduced following F10 IC versus control IC and IL-2/602 complex treatment in the short-term study. Consistent with the B16F10 therapeutic study, CD8^+^ T_MP_ numbers significantly increased and PD-1^+^CD8^+^ T cell numbers trended higher in the spleen of mice treated with F10 IC versus control IC (Supplemental Figure 12E). IL-2/602 complex did not expand PD-1^+^ CD8^+^ T cells or CD8^+^ T_MPs_ in the spleen. PD-1^+^ CD8^+^ T cells and CD8^+^ T_MP_ numbers were not significantly changed in the TME following treatment with IL-2/602 complex or IC versus PBS (Supplemental Figure 12F). Taken together, mechanistic studies established that F10 IC leads to both acute and chronic systemic and TME upregulation of Effs.

F10 IC was not toxic in non-tumor-bearing BALB/c mice in terms of pulmonary wet weight or AST/ALT. To probe toxicity in tumor-bearing mice, we analyzed cytokine secretion and liver toxicity in our B16F10 short-term study. No significant changes in IL-1α, IL-6, IL-10, IL-12 p70, IL-17A, IL-23, IL-27, IFN-β, TNF-α, GM-CSF, or monocyte chemoattractant protein–1 levels were observed between PBS, control IC, IL-2/602 complex, and F10 IC treatment (Supplemental Figure 13A). IL-2/602 complex, but not ICs, induced elevated IL-1β levels compared with PBS treatment, and control IC, but not IL-2/602 complex or F10 IC, increased IFN-γ levels relative to PBS treatment. Liver toxicity assessment showed no significant changes in AST, ALT, or albumin between PBS, control IC, IL-2/602 complex, and F10 IC treatment (Supplemental Figure 13B). Bilirubin levels were decreased compared with PBS for IL-2/602 complex but not IC treatment. Overall, these data suggest that F10 IC does not induce cytokine storm or liver damage.

**Figure 8:**
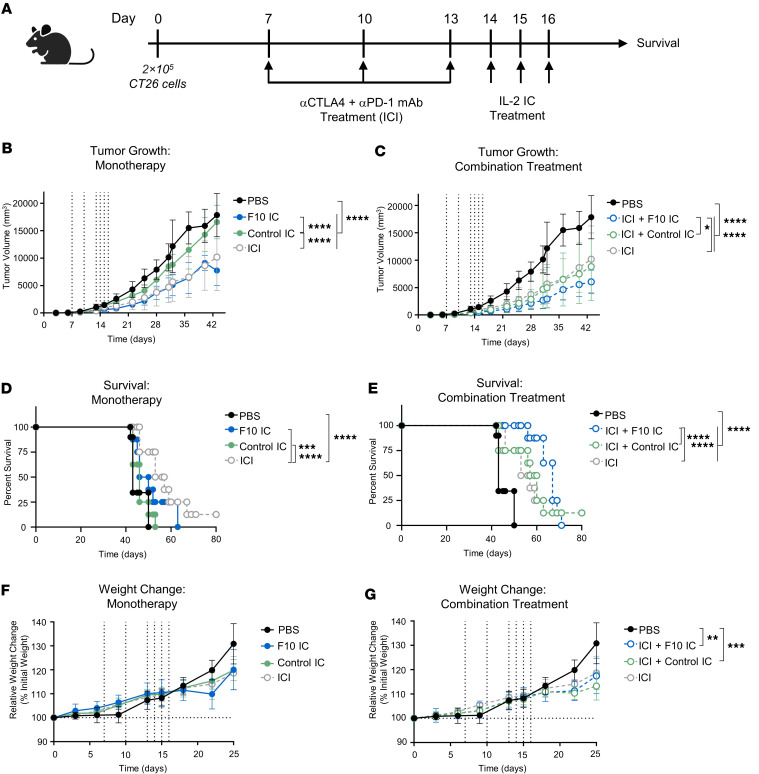
F10 IC, alone or in combination with ICIs, improves the therapeutic efficacy of IL-2 in late-stage tumor progression. (**A**) Schematic of CT26 colorectal carcinoma model design. (**B**–**G**) BALB/c mice (*n* = 8–10) were injected subcutaneously with CT26 tumor cells and treated with either PBS or a combination of anti–PD-1 and anti–CTLA-4 antibodies (0.5 mg/kg each, ICI), followed by intraperitoneal treatment with either PBS or the molar equivalent of 0.125 mg/kg IL-2 of control IC or F10 IC. Tumor volume (**B** and **C**), survival (**D** and **E**), and percentage changes in body weight relative to weight at the time of tumor implantation (**F** and **G**) are shown. Data are presented as mean ± SEM. *P < 0.05, **P < 0.01, ***P < 0.001, ****P < 0.0001 by 2-way ANOVA test with Tukey’s test. For tumor growth, mouse survival, and mouse weight curves, significance is indicated for overall curves. Data are from a single experiment, parsed for visual clarity; thus, the curves for PBS and ICIs in tumor volume (**B** and **C**), survival (**D** and **E**), and weight change (**F** and **G**) graphs depict the same experimental group.

Building on our promising cancer models, we wondered whether F10 IC would be effective in controlling established tumors. Motivated by the observed systemic upregulation of PD-1 on CD8^+^ T cells and CD8^+^ T_MPs_ (Supplemental Figure 10E, Supplemental Figure 11E, and Supplemental Figure 12E) and recent studies demonstrating the therapeutic benefit of combining IL-2 with ICIs (22, 55–59), we treated CT26 tumor–bearing mice with PBS, control IC, or F10 IC alone or with administration of anti–PD-1 and anti–cytotoxic T lymphocyte–associated protein 4 (CTLA-4) antibodies (Figure 8A). For monotherapies, F10 IC treatment significantly suppressed tumor growth, comparable to ICIs, whereas control IC suppressed tumor growth to a lesser extent (Figure 8B). ICIs significantly prolonged survival versus PBS treatment, unlike ICs (Figure 8D). No significant weight changes were observed for any monotherapy treatments (Figure 8F). For combination treatments, ICI+F10 IC, but not ICI+control IC, significantly reduced tumor burden compared with ICI monotherapy (Figure 8C). Furthermore, ICI+F10 IC significantly extended survival compared with ICI monotherapy, unlike ICI+F10 IC treatment (Figure 8E). ICI+control IC and ICI+F10 IC led to lower weight measurements at late time points because of significantly smaller tumor burden, but no weight loss was observed during the treatment period (Figure 8G), indicating that F10 IC is well tolerated alone and in combination with ICIs.

Collectively, short-term and long-term studies in multiple early and established tumor models demonstrated that F10 IC effectively drives antitumor activity without inducing severe toxicities associated with IL-2 immunotherapy. F10 IC was more effective in suppressing tumor growth than control IC and IL-2/antibody complex, alone or in combination with ICIs, and synergy was observed between F10 IC and ICIs for inhibition of tumor growth and extension of survival.

## Discussion

This study engineered F10 IC, a cytokine/antibody fusion protein in which the component antibody intramolecularly binds IL-2 and blocks its interaction with IL-2Rα to promote expansion of pro-inflammatory immune cells. IL-2 therapy has long been hampered by the cytokine’s indiscriminate expansion of both pro- and antiinflammatory cells as well as its short half-life. Our optimized F10 IC molecule exhibits robust bias toward expansion of Effs over T_regs_, boosting antitumor activity alone or in combination with ICIs without inducing toxicity, while dramatically improving cytokine half-life.

Numerous other IL-2–based molecules that bias activity toward Effs by disrupting IL-2/IL-2Rα interactions have been described (15–28, 42). Many of these molecules include mutations of the native hIL-2 sequence to eliminate IL-2Rα interactions (19, 21, 23–25, 27), and most have additional mutations to enhance interactions with IL-2Rβ and γ_C_ (19, 21, 23, 25, 27). While some molecules genetically fuse mutated IL-2 to other proteins (19, 23, 25, 27) or conjugate it to PEG (31, 32), others are limited by short half-life. Moreover, mutations to IL-2 may reduce its stability and/or increase the chances of developing neutralizing antidrug antibodies (33). Further, directly engineering IL-2 to interact more strongly with IL-2Rβ and γ_C_ can lead to systemic increases in cytokine activity, resulting in off-target T cell activation. Despite these concerns, several engineered IL-2 molecules are under active clinical investigation (19, 21, 25, 28, 42), suggesting that each iterative improvement can bring them closer to therapeutic application. Alternative strategies involving IL-2/anti–IL-2 antibody complexes that block IL-2Rα interaction have been developed, but most do not fuse IL-2 to the antibody (22, 36–38) and therefore allow cytokine dissociation, leading to pleiotropic activities and rapid clearance. Our efforts demonstrated that the enhanced stability and bias resulting from cytokine/antibody fusion significantly improved tumor suppression compared with IL-2/antibody complex treatment. Recently, a molecule known as NARA1leukin grafted IL-2 into the binding domain of the NARA1 antibody (41, 42). This unimolecular formulation is being evaluated in clinical trials (NCT04855929, NCT05578872), illustrating the promise for translating cytokine/antibody fusion proteins. Our modular approach carries several advantages over existing cytokine-engineering strategies, including (i) our engineered IC strongly biases the cytokine toward Effs, leading to >5-fold enhancement of the CD8^+^ effector T cell and NK cell to T_reg_ ratios in mice compared with native IL-2, in both the absence and presence of tumors; (ii) fusion of IL-2 to an antibody improves the stability and persistence of the cytokine through FcRn-mediated recycling (60); (iii) genetic fusion of the IL-2 cytokine avoids chemical modification, facilitating formulation; (iv) use of native IL-2 rather than a mutein circumvents immunogenicity concerns; and (v) retention of the IL-2Rα interface of the IL-2 cytokine allows for synergy with ICIs. Further, compared with previous IL-2 immunocytokine strategies, the modular terminal cytokine fusion strategy we employ allows for ready extension of our approach to other anti–IL-2 antibodies as well as other cytokine systems of interest.

Our findings demonstrated that nonoptimal fusion of IL-2 to the antibody dramatically diminishes the potency and biasing of the IC. Reduced signaling activity observed in 602 IC LN15 and LN25 seemed to contradict the apparent functionality of all ICs based on binding studies. It is possible that shorter linker ICs attenuate signaling by disrupting the interaction between IL-2 and γ_C_ through interference with the γ_C_ binding site on the cytokine, which is immediately adjacent to the linker at the C-terminus of IL-2. Thus, these engineering observations show that maximizing IC activity requires optimizing cytokine/antibody fusion format, and fusions that enforce unfavorable conformations can differentially ablate cytokine functions.

Linker optimization was also critical for maximizing the yield of monomeric IC. During elution from protein G, the IC is highly concentrated, and low pH destabilizes noncovalent binding interactions. These conditions are prime for the exchange of IL-2 between proximal ICs, which leads to oligomerization. Indeed, 602 ICs with short linkers showed increased oligomerization, suggesting these molecules disfavored intramolecular interaction between the cytokine and antibody within the IC. Extending the linker reduced intramolecular instability, favoring monomeric assembly of ICs. These conclusions were corroborated by structural data, which demonstrated that a linker greater than 15 amino acids is required for *cis* interactions between an IL-2 moiety and its tethered 602 antibody variable domains within the IC. Moreover, structural findings suggest that linker lengths greater than 58 amino acids could lead to *trans* interactions between an IL-2 moiety and nontethered 602 antibody variable domains within the IC, which could impair cytokine function. These results are consistent with another recently reported IL-2–based IC, wherein a 35–amino acid linker was found to minimize oligomerization and optimize cytokine activity compared with 15– and 25–amino acid linkers (60). Other sources of IC aggregation may exist, and it will be critical to understand the contaminant species present in IC formulations for therapeutic translation of F10 IC and other similar molecules. Moreover, detailed optimization of F10 IC production with respect to buffer conditions, elution pH, and temperature conditions must be performed to enable reproducible and scalable manufacturing.

Our 602 engineering campaign employed a competitive selection approach to further enhance antibody bias toward Eff expansion. Structural data revealed that 602 sterically blocked IL-2/IL-2Rα binding without interfering with IL-2/IL-2Rβ or IL-2/γ_C_ interactions. Moreover, 602 reproduced a receptor-induced conformational change in the C helix of IL-2, which allosterically potentiates IL-2Rβ binding (12, 17, 39). F10 recapitulated these structural properties of 602 but also introduced an additional salt bridge at the cytokine/antibody interface that led to higher affinity interaction. A slight positional shift was also observed between the 602 and F10 backbones. Collectively, the structural modifications induced by the F10 mutations promoted greater functional bias toward Effs. Importantly, in contrast with previous efforts in generating IL-2 muteins (21, 23, 27), the structural advantages for F10 IC were realized without modifying the IL-2 sequence, thus improving bias toward Effs without increasing potential immunogenicity or compromising the native activity of IL-2.

Comparison with other reported anti–IL-2 antibodies that bias the cytokine toward expansion of Effs revealed a unique binding topology for 602 and F10, despite their functional overlap with these other antibodies. Interestingly, despite the divergent binding orientations of the 602/F10 and S4B6 antibodies, the overlap between the 602/F10 and IL-2Rα interfaces on hIL-2 was remarkably similar to the overlap between the predicted S4B6 and resolved IL-2Rα interfaces on hIL-2. However, NARA1 bound IL-2 with a very different topology relative to 602/F10 and showed divergent overlap with the IL-2Rα interface on hIL-2. These structural observations suggest that distinct antibody-based IL-2Rα occlusion strategies can lead to convergent functional outcomes.

No differences were observed between the intracellular programs induced in CD8^+^ T cells treated with IL-2 versus ICs based on RNA-Seq analysis. However, in vivo studies revealed differences in T_reg_ expansion between control IC and IL-2/602 complex. While it is possible that intracellular signaling pathways in T_regs_ are more sensitive to differences between IL-2 and ICs than those in CD8^+^ T cells (61, 62), our comparisons between native Fc and Fc with effector function knocked out suggest that differences in T_reg_ expansion following control IC treatment can be at least partially explained by Fc-mediated depletion of cells that bind, but do not immediately internalize, the IC. High expression of the noninternalizing IL-2Rα subunit (53) on T_regs_ and high expression of IL-2Rβ on CD8^+^ T_MPs_ (22) could account for the impact of Fc knockout on control IC in these cell lines (Supplemental Figure 9, A and B). However, since Fc knockout did not fully restore control IC–mediated expansion in T_regs_, additional factors such as receptor binding kinetics, internalization, and/or endosomal processing may also contribute (35, 62).

Elucidating the pharmacokinetic behavior and cellular processing dynamics of ICs will be essential for clinical translation, specifically for optimizing dosing amounts and schedules. As effector T cells transiently express IL-2Rα once activated (1, 2), dosing of our IC must be carefully considered to best capitalize on this immune feedback. Moreover, characterizing receptor expression dynamics will inform dosing regimens, particularly for combination treatments. Our study explored multiple treatment regimens for monotherapy and combination approaches. We found that later stage treatment with F10 IC alone in established tumor models significantly suppressed tumor growth without inducing systemic toxicity, on par with ICI monotherapy. Moreover, our combinatorial approach of pretreatment with ICIs followed by F10 IC administration further increased the efficacy of both therapies in terms of tumor growth inhibition and survival, again without inducing systemic toxicity. The safety and efficacy of F10 IC across multiple mouse models of cancer, in monotherapy and combination treatments, motivate further preclinical development of this molecule.

To realize clinical translation of F10 IC, several factors must be considered. We developed F10 IC by fusing hIL-2 to a mouse antibody; thus, both the variable and constant domains of our antibody will need to be humanized to mitigate immunogenicity concerns. Nonetheless, use of a mouse antibody in our syngeneic tumor models likely provided useful predictions for neutralization, toxicity, and other immune-mediated effects of a humanized antibody administered to human patients. We also note that F10 IC in vivo evaluation was limited to syngeneic mouse tumors. As with other hIL-2–based therapies, efficacy relies on the presence of tumor-specific Effs in the TME on the ratio of Effs to T_regs_. We observed trends of CD8^+^ T cell and NK cell expansion relative to T_regs_ within the TME in syngeneic models; thus, analysis in additional settings, such as spontaneous disease models and humanized mouse models, will help further characterize therapeutic performance. Of note, due to the unmodified state of IL-2 within F10 IC, we envision that T and NK cells (which were expanded in the spleens and TME of tumor-bearing mice) play a critical role in mediating F10 IC antitumor responses, as has been observed for previous studies with native IL-2 and IL-2–based cytokines (23, 27, 63, 64). Subsequent investigations in nonhuman primates will also be needed to validate the safety, pharmacokinetic behavior, biodistribution, and efficacy of our molecule prior to clinical advancement.

Reports involving other IL-2 muteins, cytokine/antibody complexes, and fusion proteins that block the IL-2/IL-2Rα interaction and enhance IL-2 interaction with IL-2Rβ and γ_C_ demonstrate that combining F10 IC with additional cancer therapeutics beyond ICIs, such as cancer vaccines (42), or adoptive cell transfer (41), further augments tumor suppression. Given the numerous active clinical programs involving engineered IL-2 proteins (28), the stability, selectivity, safety, and antitumor efficacy of F10 IC make it appealing for translation. Moreover, the modularity of its single-molecule construction readily invites adaptation to additional formats that will enable greater homing to and retention in the tumor.

## Methods

For full methods, see Supplemental Methods.

### Sex as a biological variable.

Female 8- to 12-week-old C57BL/6 and BALB/c mice were obtained from the colony kept at the Czech Centre for Phenogenomics or purchased from The Jackson Laboratory. Our study exclusively examined female mice. It is unknown whether the findings are relevant for male mice.

### Statistics.

Data were graphed and analyzed using GraphPad Prism v10.2 or earlier and expressed as mean ± SEM for in vivo summary data or as mean ± SD for non–in vivo summary data. Summary data were analyzed with 2-way ANOVA (tumor growth, survival, and weight change) or 1-way ANOVA (3 cohorts’ comparison), both with Tukey’s test, or with 2-tailed *t* test (2 cohorts’ comparison). A *P* value below 0.05 was used as the threshold for statistical significance. For tumor growth, mouse survival, and mouse weight curves, significance levels are indicated for the overall curves. Complete statistical analyses are provided in Supplemental Table 8.

### Study approval.

Mice were housed under specific pathogen–free conditions and handled according to the institutional committee guidelines. Animal experiments were approved by the Animal Care and Use Committee of the Institute of Molecular Genetics (Prague, Czech Republic) and were in agreement with local legal requirements and ethical guidelines or conducted in accordance with the Johns Hopkins University Animal Care and Use Committee under protocol number MO20M285.

### Data availability.

Coordinates and structure factors for the IL-2/602 scFv and IL- 2/F10 scFv complexes are deposited in the Worldwide PDB public repository (PDB ID IL-2/602 scFv: 8SOZ; PDB ID - IL-2/F10 scFv: 8SOW). RNA-Seq data have been deposited into the NCBI Gene Expression Omnibus with accession numbers GSM8428011–GSM8428024. All data associated with this study are present in the paper, the supplemental materials, and the Supporting Data Values table.

## Author contributions

For full author contributions, see supplement.

## Supplementary Material

Supplemental data

Unedited blot and gel images

Supplemental table 8

Supporting data values

## Version 1 08/08/2024

In-Press Preview

## Version 2 09/24/2024

Electronic publication
