# Supplementary material for: Engineered cytokine/antibody fusion proteins improve IL-2 delivery to pro-inflammatory cells and promote antitumor activity
Source: JCI Insight. 2024 Sep 24;9(18):e173469. doi: 10.1172/jci.insight.173469 (PMC11457862; doi:10.1172/jci.insight.173469)
Supplement: Supplemental data [file jciinsight-9-173469-s260.pdf]

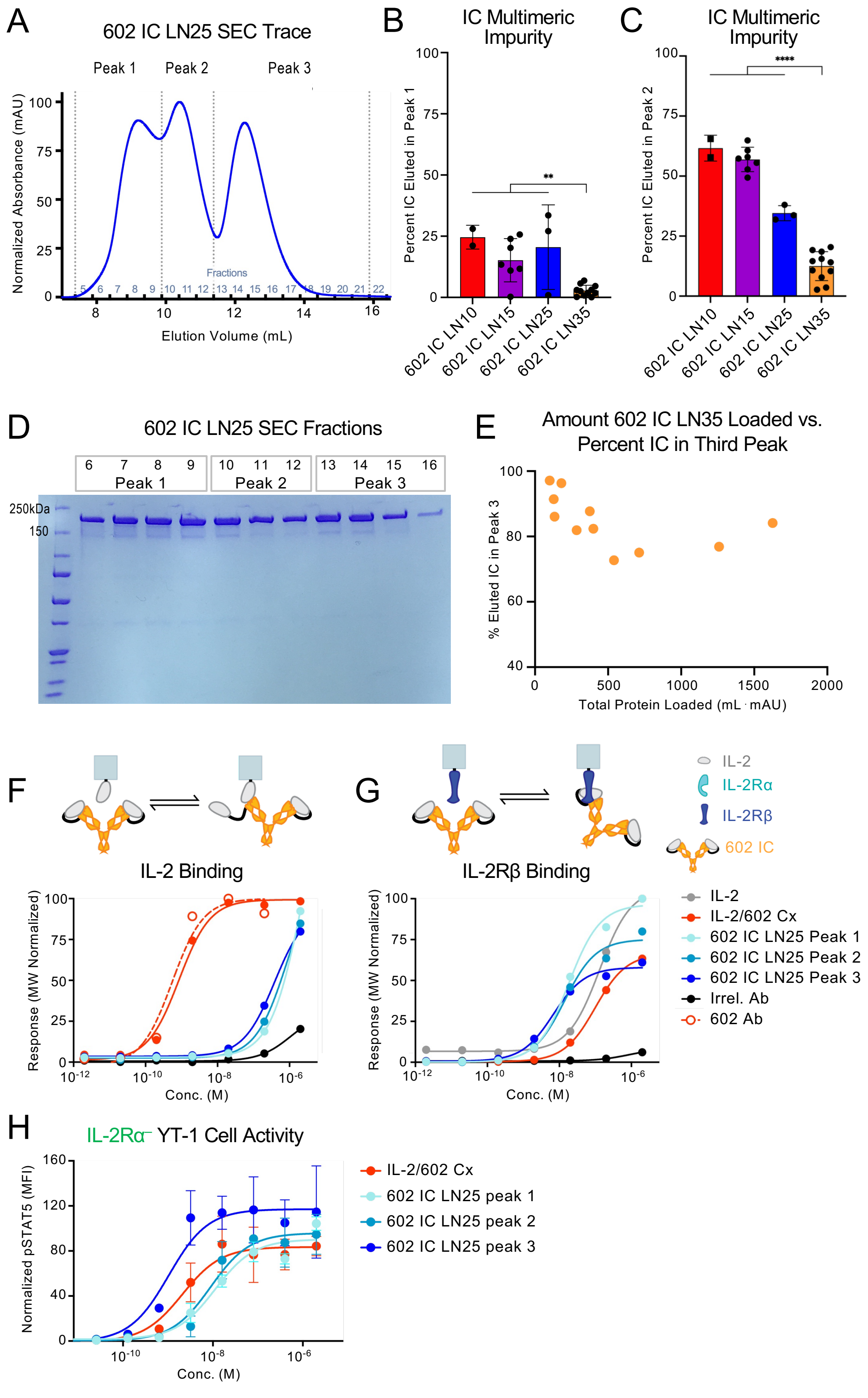

Supplemental Figure 1.

Supplemental Figure 1. Optimization of immunocytokine linker length. **(A)** Representative size-exclusion chromatography (SEC) trace shows the distribution of 602 IC LN25 in each of the 3 peaks, representing differential oligomeric states of IC. **(B, C)** The average percentage of each IC that eluted in the first **(B)** and second **(C)** peaks based on the area under the SEC curve is shown. Data represent mean ± SD from at least 2, and up to 11, purifications. Statistical significance was determined by one-way ANOVA with Tukey's multiple comparison test and is noted only for comparisons to 602 IC LN35. **P<0.01, ****P<0.0001. **(D)** SEC elution fractions 6 to 16 of 602 IC LN25 (as annotated in **(A)**) show identical migration by SDS-PAGE under non-reducing conditions. **(E)** The percentage of IC that eluted in the third peak compared to the total amount of 602 IC LN35 that was loaded per SEC run. Total amount of protein was measured as the total area under all SEC three peaks (500 mL·mAU is approximately equal to a loading concentration of 7.5 μM. **(F)** Equilibrium bio-layer interferometry (BLI) titrations of soluble IL-2/602 complex (Cx) (1:1 molar ratio), 602 antibody (Ab), and 602 IC LN25 peaks 1, 2, and 3 against immobilized IL-2. An antibody with irrelevant specificity is included as a negative control. **(G)** Equilibrium BLI titrations of soluble IL-2, IL-2/602 Cx (1:1 cytokine:antibody molar ratio), 602 antibody (Ab), and 602 IC LN25 peaks 1, 2, and 3 against immobilized IL-2Rβ. An antibody with irrelevant specificity (Irrel. Ab) is included as a negative control. **(H)** STAT5 phosphorylation response of IL-2Rα$^-$ YT-1 human NK cells treated with IL-2/602 Cx (1:1 molar ratio) and 602 IC LN25 peaks 1, 2, and 3. Data represent mean ± SD (n=2).

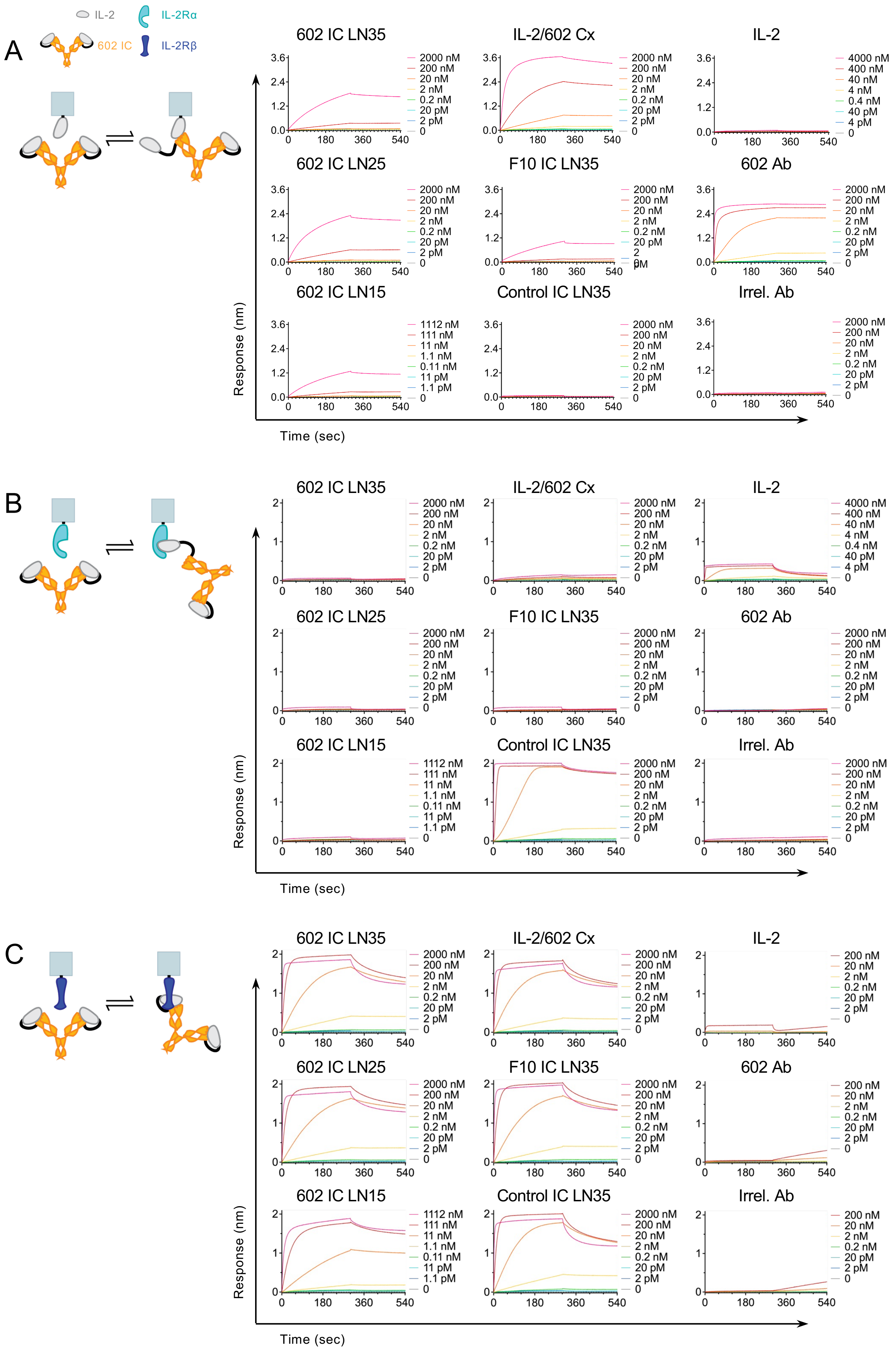

Supplemental Figure 2.

Supplemental Figure 2. Interaction of IC formulations with IL-2 cytokine and receptor subunits. Bio-layer interferometry (BLI) kinetic traces depicting soluble IL-2, IL-2/602 complex (Cx) (2:1 cytokine:antibody molar ratio), 602 antibody (Ab), 602 IC LN15, LN25, and LN35, F10 IC, Control IC, or an antibody of irrelevant specificity (Irrel. Ab) binding to immobilized IL-2 **(A)**, IL-2Rα **(B)**, and IL-2Rβ **(C)** are shown. Equilibrium titrations corresponding to these traces are presented in **Figures 2** and **3**.

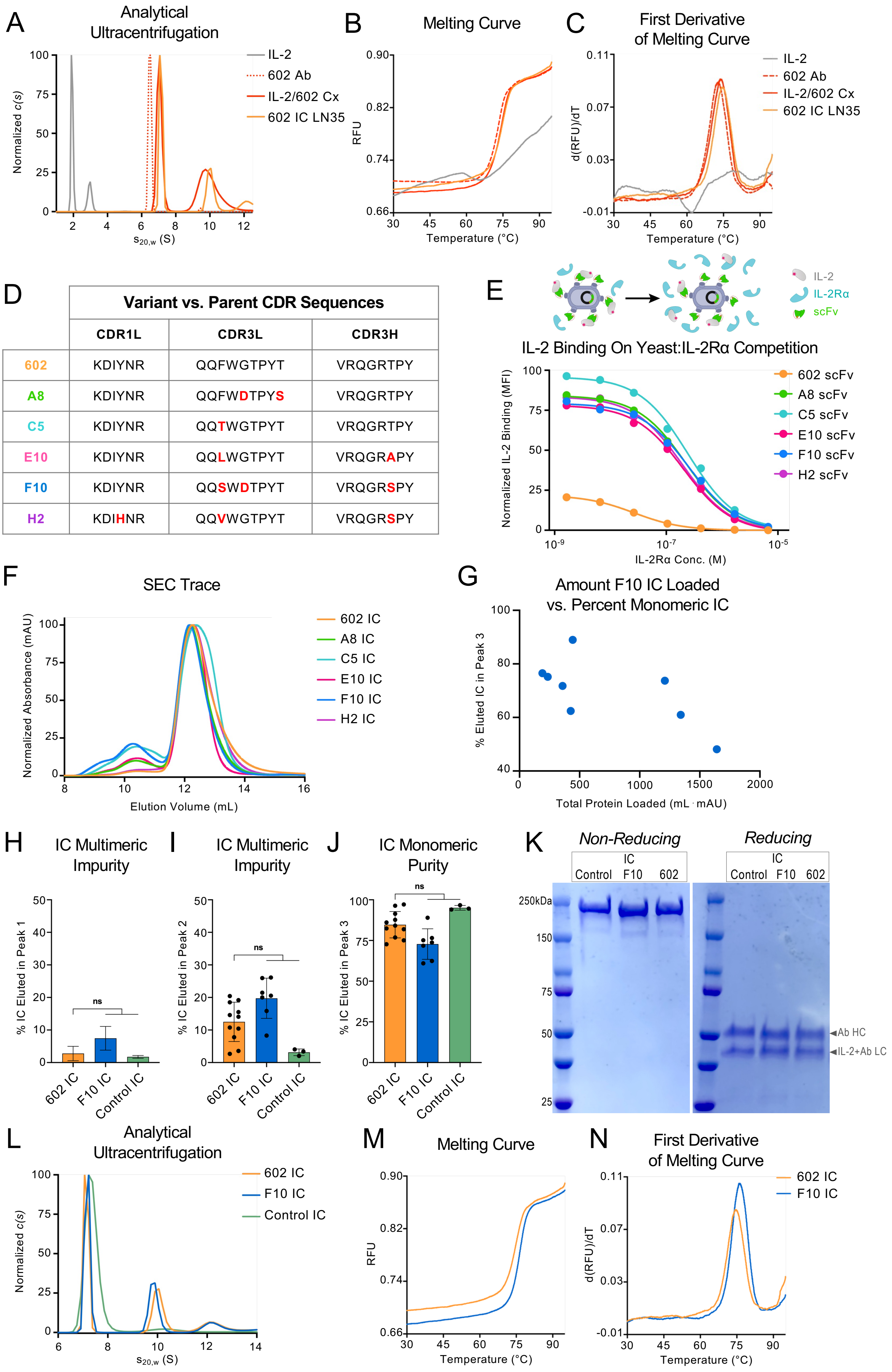

	Variant vs. Parent CDR Sequences		
	CDR1L	CDR3L	CDR3H
602	KDIYNR	QQFWGTPYT	VRQGRTPY
A8	KDIYNR	QQFWDTPYS	VRQGRTPY
C5	KDIYNR	QQTWGTPYT	VRQGRTPY
E10	KDIYNR	QQLWGTPYT	VRQGRAPY
F10	KDIYNR	QQSWDTPYT	VRQGRSPY
H2	KDIHNR	QQVWGTPYT	VRQGRSPY

Supplemental Figure 3.

Supplemental Figure 3. Characterization of representative 602 variants from evolved mutagenic library. **(A)** Analytical ultracentrifugation analysis of unconjugated IL-2, 602 antibody (Ab), IL-2/602 complex (Cx) (2:1 cytokine:antibody molar ratio), and 602 IC LN35. 602 IC LN35 and IL-2/602 Cx show main species sedimenting at 6.5–7.5 S, corresponding to the expected size. Minor peaks are also observed for both, representing dimeric and trimeric species. **(B, C)** Differential scanning fluorometry (DSF) profiles **(B)** and their first derivatives **(C)** for the thermal unfolding of IL-2, 602 Ab, and IL-2/602 Cx (2:1 cytokine:antibody molar ratio), and 602 IC LN35 in HBS. **(D)** Mutations to the CDRs for each of the five mutagenic 602 scFv library-derived variants (A8, C5, E10, F10, and H2) are indicated in red. **(E)** IL-2 binding (5 nM) to yeast-displayed 602 scFv compared to the five selected 602 scFv variants in the presence of titrated concentrations of IL-2Rα. Selected yeast-displayed 602 scFv variants show stronger IL-2 binding at higher IL-2Rα concentrations compared to parent 602 antibody, demonstrating superior receptor competition. **(F)** Representative size-exclusion chromatography (SEC) traces show that the majority of variant ICs elutes in the third peak, representing the monomeric construct. **(G)** Percentage of IC that eluted in the third peak versus the total amount of F10 IC loaded per SEC run. Total amount of protein was measured as the total area under all three SEC peaks (500 mL·mAU is approximately equivalent to a loading concentration of 7.5 μM). **(H-J)** The average percentage of each IC (602, F10 and Control) that eluted in the first **(H)**, second **(I)**, and third **(J)** peaks based on the computed area under the SEC curve. Data represent mean ± SD from at least 2, and up to 11, purifications. Statistical significance was determined by one-way ANOVA with Tukey's multiple comparison test. ns, not significant. **(K)** 602 IC, F10 IC, and Control IC migrate at the expected sizes by SDS-PAGE, under non-reducing (~180 kDa) and reducing (49 kDa for HC, ~41 for IC LC) conditions. **(L)** Analytical ultracentrifugation analysis of 602 IC, F10 IC, and Control IC. ICs show main species sedimenting at 6.5–7.5 S, corresponding to the expected size for each. Minor peaks are also observed, representing dimeric and trimeric species. **(M, N)** DSF profile **(M)** and its first derivative **(N)** for the thermal unfolding of 602 IC, and F10 IC in HBS.

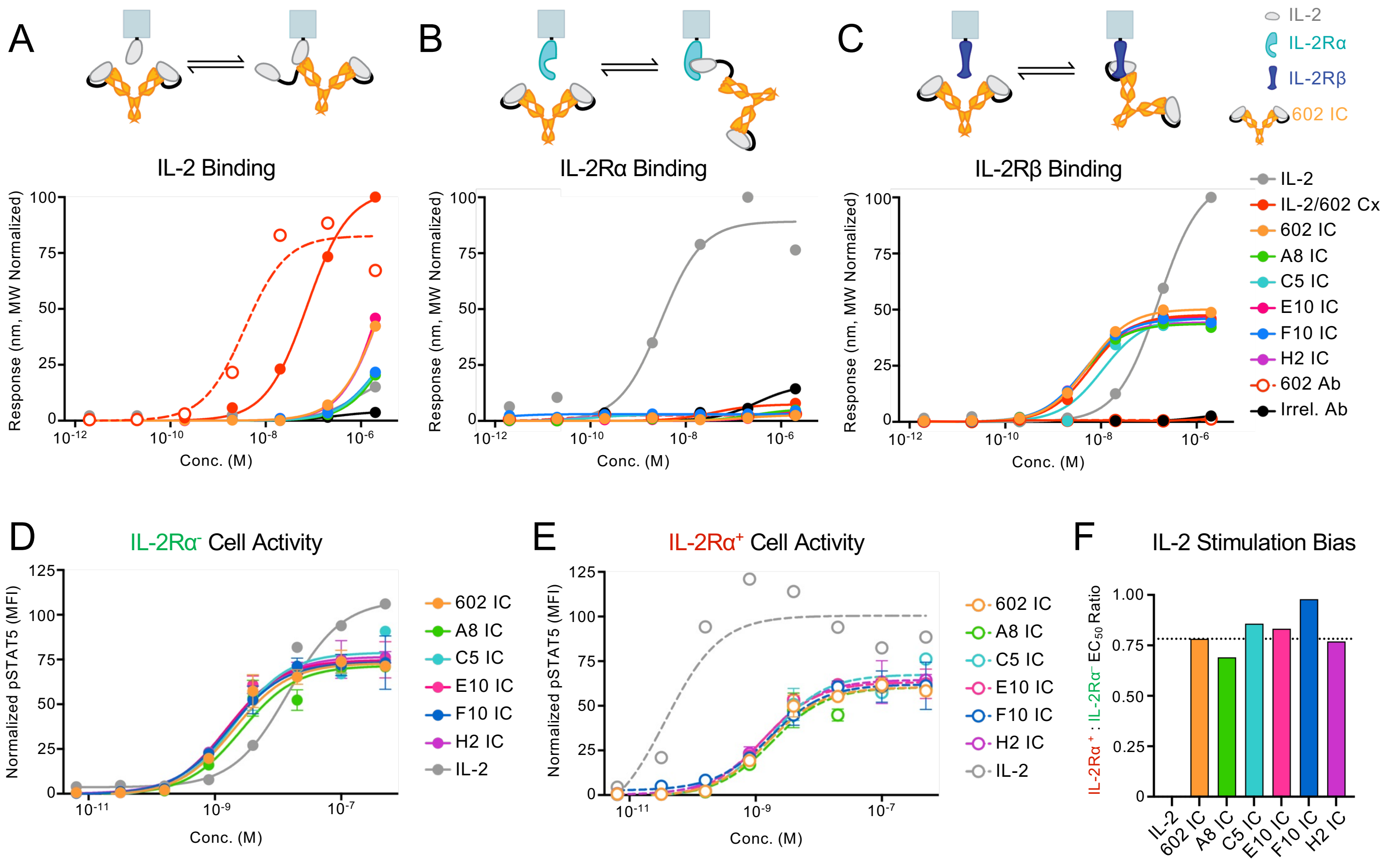

Supplemental Figure 4.

Supplemental Figure 4. Functional activities of representative engineered 602 IC variants. **(A-C)** Equilibrium bio-layer interferometry (BLI) titrations of soluble IL-2, IL-2/602 complex (Cx) (2:1 cytokine:antibody molar ratio), 602 antibody (Ab), 602 IC, 5 engineered 602 IC variants (A8, C5, E10, F10, and H2), and an antibody of irrelevant specificity (Irrel. Ab) binding to immobilized IL-2 **(A)**, immobilized IL-2Rα **(B)**, and immobilized IL-2Rβ **(C)**. **(D)** STAT5 phosphorylation response of IL-2Rα$^-$ YT-1 human NK cells treated with IL-2, 602 IC, and engineered 602 IC variants in a mixed IL-2Rα$^-$/IL-2Rα$^+$ cell assay. Data represent mean ± SD (n=2). **(E)** STAT5 phosphorylation response of IL-2Rα$^+$ YT-1 human NK cells treated with IL-2, 602 IC, F10 IC, and engineered 602 IC variants in a mixed IL-2Rα$^-$/IL-2Rα$^+$ cell assay. Data represent mean ± SD for at least 420 collected flow cytometry events (avg. 930 events) (n=2). **(F)** Ratio of the IL-2Rα$^+$/IL-2Rα$^-$ STAT5 phosphorylation EC_{50} values for assays shown in **(D)** and **(E)**.

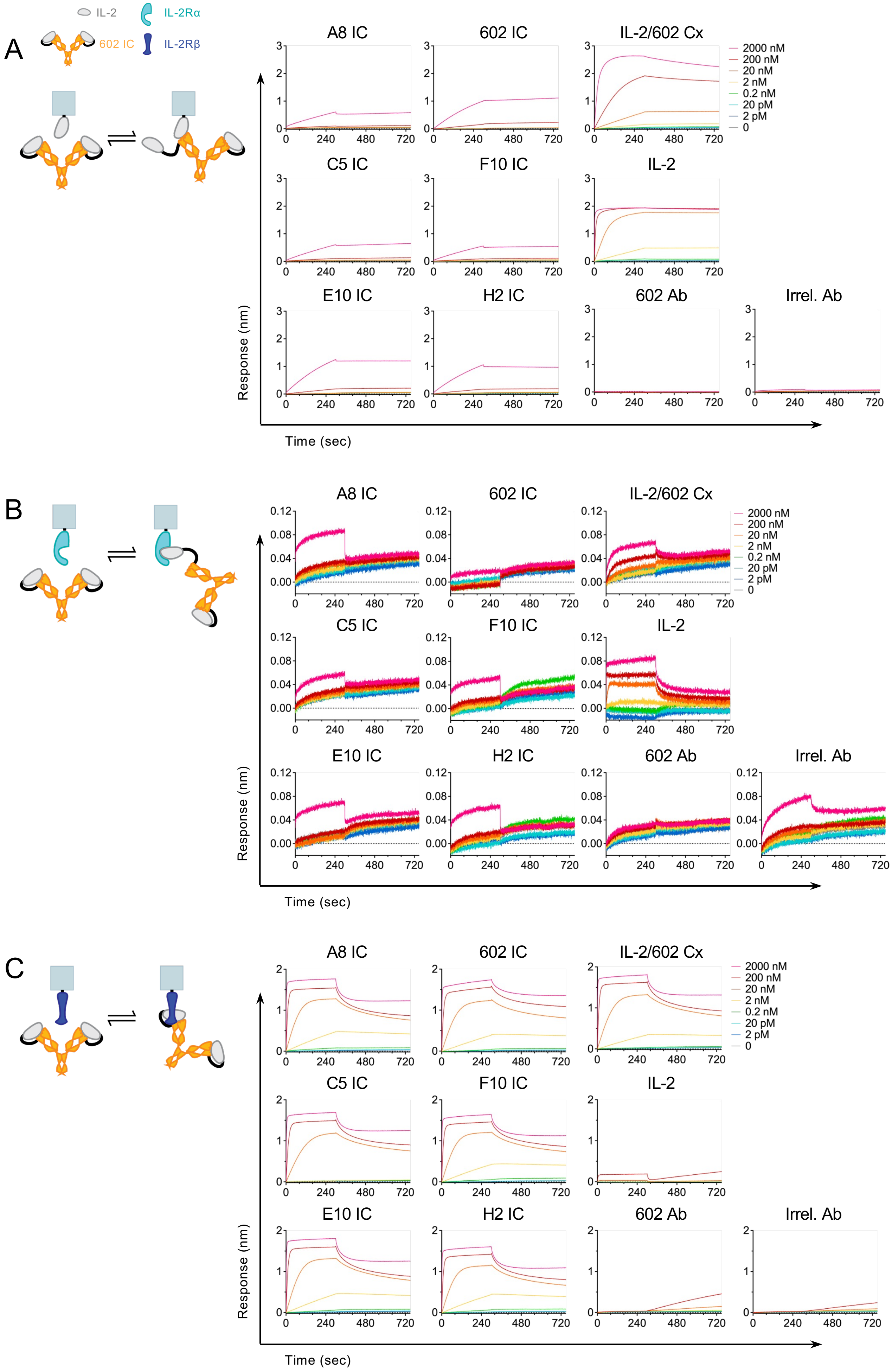

Supplemental Figure 5.

Supplemental Figure 5. Interaction of engineered 602 IC variants with IL-2 cytokine and receptor subunits. Bio-layer interferometry (BLI) kinetic traces depicting soluble IL-2, IL-2/602 complex (Cx) (1:1 cytokine:antibody molar ratio), 602 antibody (Ab), 602 IC, and five engineered 602 IC variants (A8, C5, E10, F10, and H2) binding to immobilized IL-2 **(A)**, IL-2Rα **(B)**, and IL-2Rβ **(C)**.

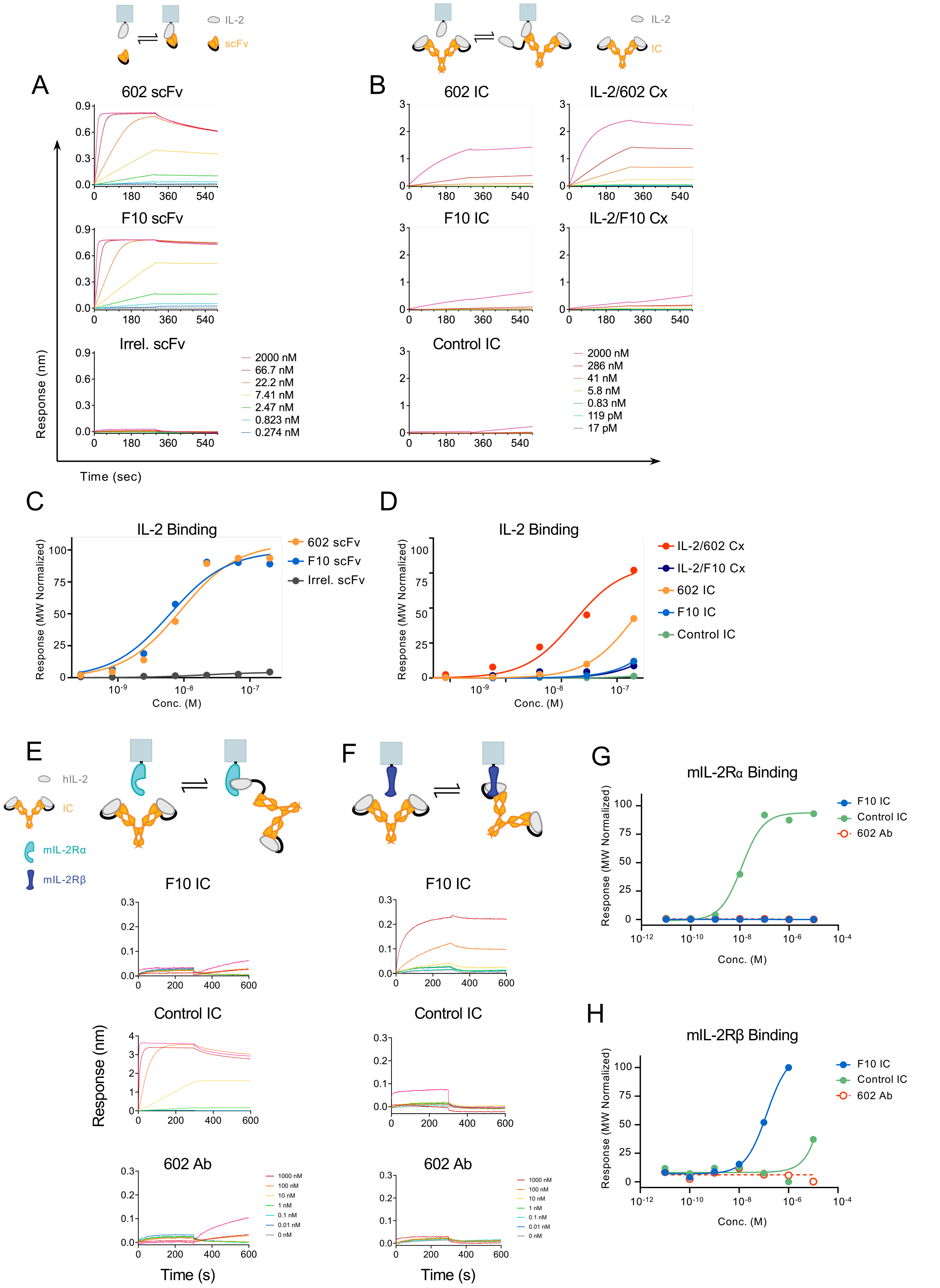

Supplemental Figure 6.

Supplemental Figure 6. Interaction of 602 and F10 with IL-2 cytokine and interaction of F10 IC with mouse IL-2 receptor subunits. (**A-D**) Bio-layer interferometry (BLI) kinetic traces (**A, B**) and equilibrium titrations (**C, D**) of scFvs, IL-2/antibody complexes (Cx), and ICs binding to immobilized human IL-2. The irrelevant scFv was an anti-mouse PD-1 scFv (clone [RMP1.14]). IL-2/antibody complexes were prepared by mixing IL-2 with the indicated antibody at a 2:1 molar ratio. (**E-H**) BLI kinetic traces (**E, F**) and equilibrium titrations (**G, H**) of ICs binding to immobilized mouse IL-2α (mIL-2Rα) (**E, G**) and mIL-2Rβ (**F, H**).

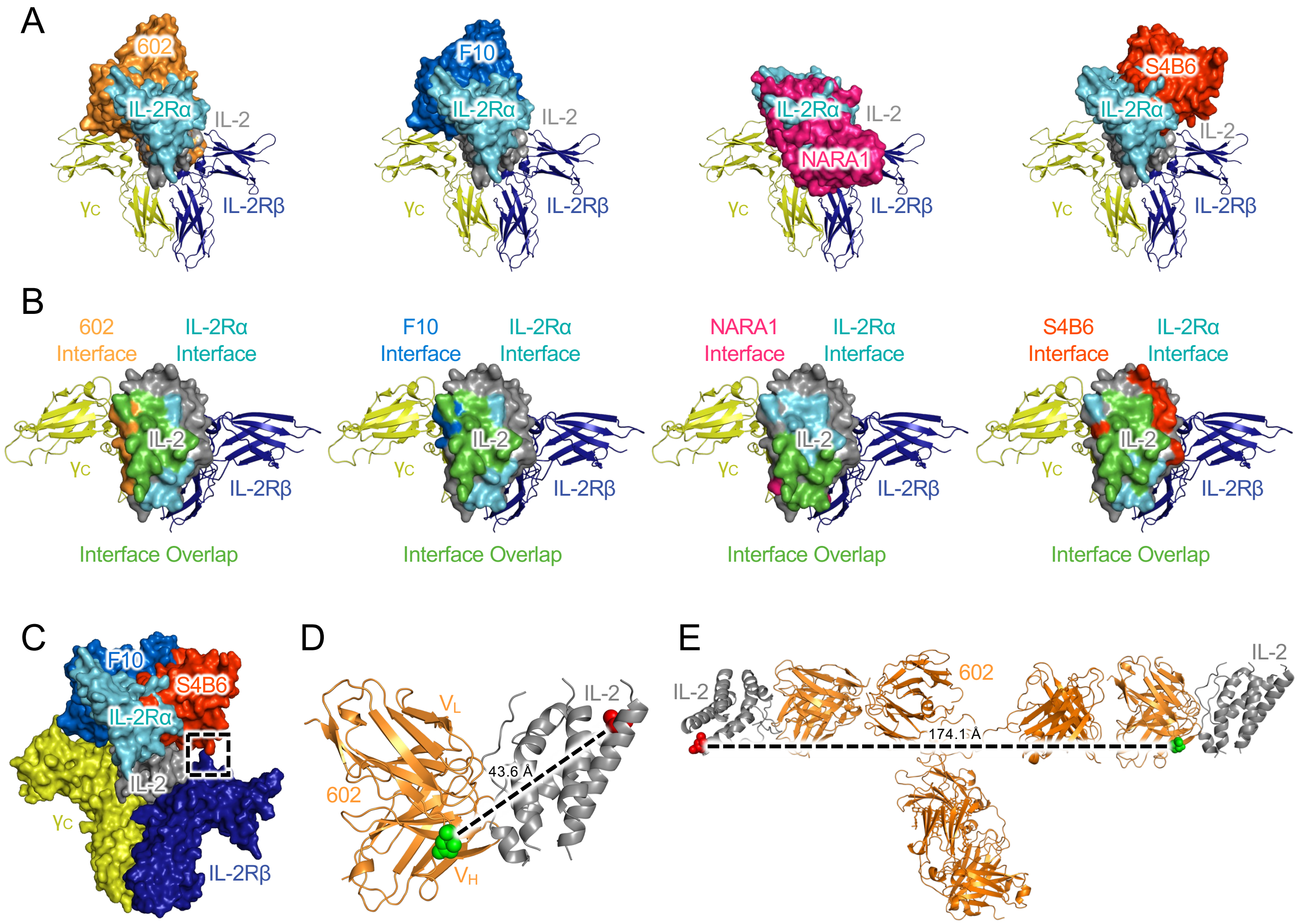

Supplemental Figure 7.

Supplemental Figure 7. Comparison of 602, F10, NARA1, and S4B6 antibody binding topologies and modeling linker considerations. (**A**) Overlay of the IL-2/antibody complex structures for 602, F10, NARA1, and S4B6 (only variable domains of the antibodies are shown) and the quaternary IL-2 cytokine/receptor complex structure (PDB 2B5I). (**B**) ‘‘Top-down’’ views of the PISA-predicted binding interface between IL-2Rα and IL-2 (cyan), and between IL-2 and the 602 (yellow-orange), F10 (blue), NARA1 (pink), and S4B6 (orange) antibodies. The binding interface for S4B6 was predicted based on alignment of the mouse IL-2/S4B6 Fab structure (PDB 4YUE) to human IL-2 bound to its high affinity trimeric receptor complex (PDB 2B5I). Overlap between the IL-2Rα and antibody binding interfaces are shown in green. (**C**) Alignment of the mouse IL-2/S4B6 complex (only antibody variable domains are shown) overlayed with the resolved crystallographic structure of hIL-2 bound to its high affinity trimeric receptor complex (PDB 2B5I) and the resolved hIL-2/F10 crystal structure. A steric clash was observed between the S4B6 antibody and IL-2Rβ (dashed black box), but not between the F10 scFv and IL-2Rβ due to the latter molecule’s shift away from the IL-2Rβ subunit. (**D**) Measurement of the linear distance between the C-terminus of IL-2 (red spheres) and the N-terminus of the 602 antibody variable light chain (green spheres) from the resolved IL-2/602 scFv complex crystal structure. Distance was measured between alpha carbons of the respective amino acids. (**E**) Model of the full length 602 IC, created by aligning the resolved IL-2/602 scFv complex crystal structure with a known isotype-matched mouse IgG2a kappa antibody structure (PDB 1IGT). The distance between the C-terminus of one IL-2 moiety (red spheres) and the N-terminus of the non-tethered 602 light chain (green spheres) is indicated. Distance was measured between alpha carbons of the respective amino acids.

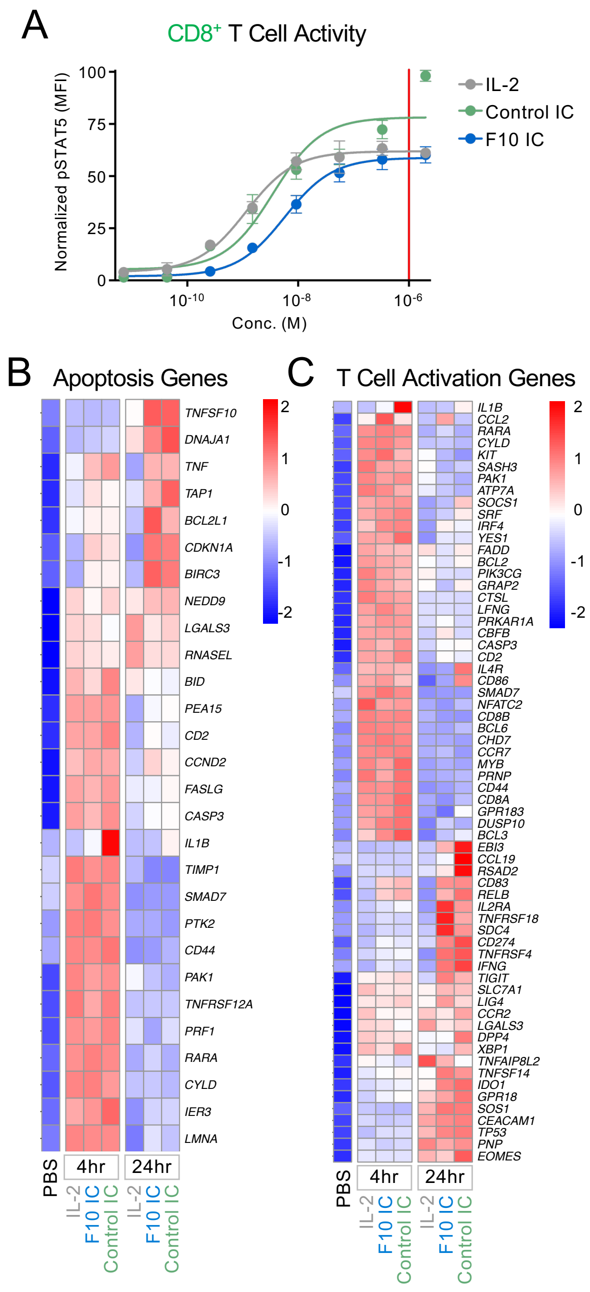

Supplemental Figure 8.

Supplemental Figure 8. Unconjugated IL-2 and ICs induce similar gene expression profiles in human CD8$^+$ T cells. **(A)** STAT5 phosphorylation response of human PBMC-derived CD8$^+$ T cells treated with IL-2, F10 IC, and Control IC. Data represent mean ± SD (n=3). Data represent mean ± SD for cells from two healthy donors (same donors that were used for RNA-Seq studies). The red line indicates the dose used for stimulation in RNA-Seq experiments (1 µM IL-2 or 0.5 µM IC). **(B, C)** RNA-Seq heatmap analysis for human CD8$^+$ T cells treated with PBS or the saturating dose of IL-2 (1 µM), F10 IC (0.5 µM), and Control IC (0.5 µM) for either 4 and 24 hr. Genes related to apoptosis **(B)** and T cell activation **(C)** are presented. The color scales in the heatmaps represent Z-score values ranging from blue (low expression) to red (high expression).

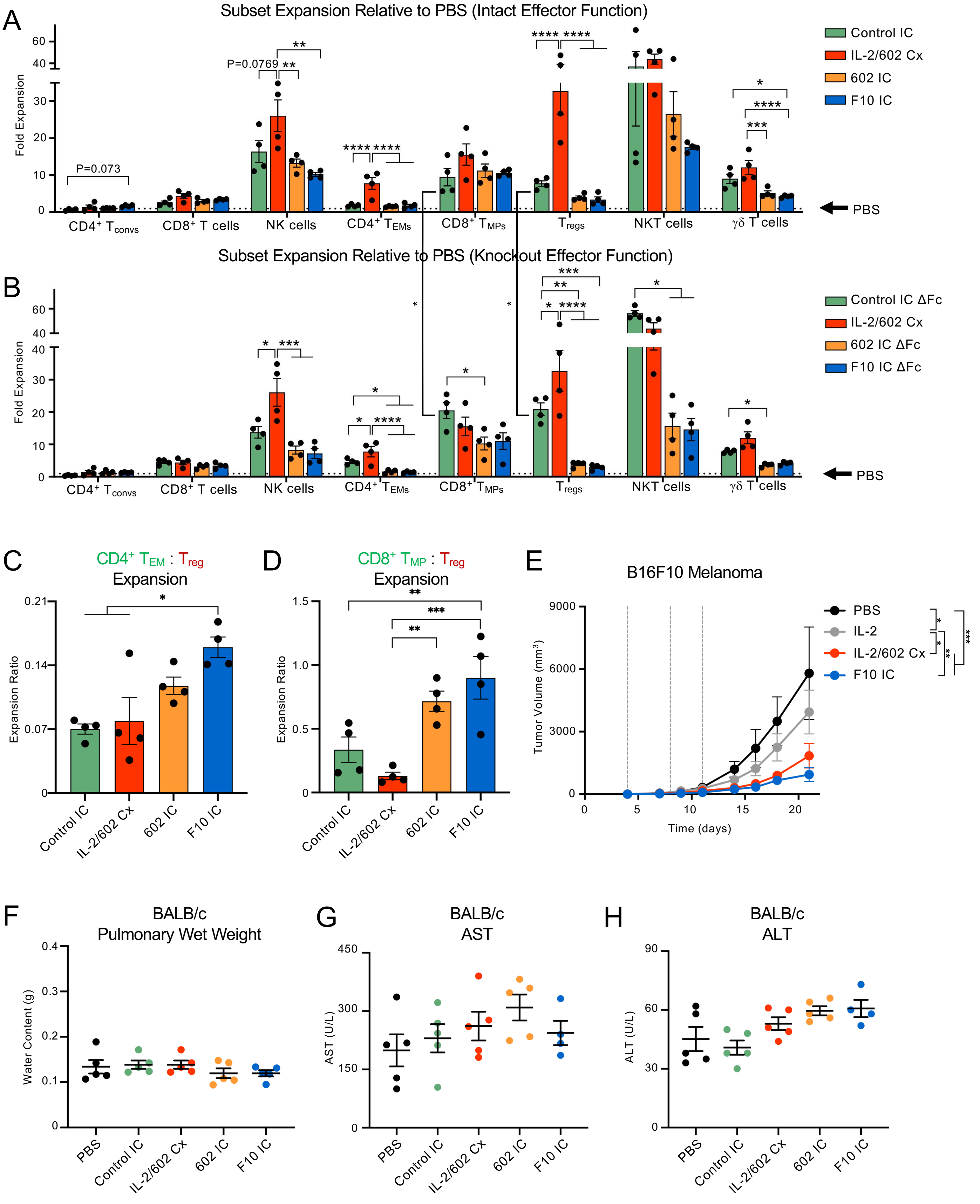

Supplemental Figure 9.

Supplemental Figure 9. Engineered F10 IC promotes selective expansion of immune effector cells and improves the therapeutic efficacy of IL-2 without inducing toxicity. **(A-C)** C57BL/6 mice (n=4) were injected intraperitoneally daily for 4 days with the molar equivalent of 0.125 mg/kg IL-2/dose of Control IC, IL-2/602 complex (Cx) (2:1 cytokine:antibody molar ratio), 602 IC, or F10 IC. Mice were sacrificed on day 5, and spleens were harvested. The total cell counts of conventional T cells ($CD4^+$ T_{convs}, $CD3^+CD4^+FoxP3^-$), $CD8^+$ T cells ($CD3^+CD4^-CD8^+$), natural killer (NK) cells ($CD3^-CD49b^+CD161^+$), $CD4^+$ effector memory T cells ($CD4^+$ T_{EMs}, $CD3^+CD4^+CD8^-CD44^+CD62L^-$), $CD8^+$ memory phenotype T cells ($CD8^+$ T_{MPs}, $CD3^+CD4^-CD8^+CD44^+CD122^+$), and T regulatory cells (T_{regs}, $CD3^+CD4^+CD25^+Foxp3^+)^+$), natural killer T (NKT) cells ($CD3^+\gamma\delta TCR^-CD49b^+CD161^+$), and gamma delta ($\gamma\delta$) T cells ($CD3^+\gamma\delta TCR^+$) in each spleen were determined by flow cytometry. The cell subset counts for each treatment divided by the cell subset count for PBS is shown (wild type antibody Fc regions in **A**, antibody Fc regions with effector function knockout [ΔFc] in **B**), as are the ratios of $CD4^+$ T_{EMs} to T_{regs} **(C)** and $CD8^+$ T_{MPs} to T_{regs} **(D)**. The dotted line in **(A)** indicates equivalent expansion to PBS treatment. **(E)** C57BL/6 mice (n=8) were injected subcutaneously s.c. with B16F10 tumor cells and treated intraperitoneally on days 2, 4, and 7 with PBS, 0.125 mg/kg IL-2, or the molar equivalent of 0.125 mg/kg IL-2 of IL-2/602 complex (Cx) (2:1 cytokine:antibody molar ratio) or F10 IC. Tumor volume is shown. **(F-H)** BALB/c mice (n=4-5/group) were injected daily for 4 days with PBS, 0.075 mg/kg IL-2/dose, or the molar equivalent of 0.075 mg/kg IL-2/dose of Control IC, IL-2/602 complex (Cx) (2:1 cytokine:antibody molar ratio), 602 IC, or F10 IC. Mice were sacrificed on day 5, and pulmonary wet weight **(H),** as well as serum concentrations of AST **(F)** and ALT **(G)** were measured. Data are shown as means ± SEM. Statistical significance was determined by one-way ANOVA with Tukey's multiple comparison test for immune cell subset expansion data and by two-way ANOVA test for tumor growth data: *$P<0.05$, **$P<0.01$, ***$P<0.001$, ****$P<0.0001$. No significant differences were observed in AST, ALT, or pulmonary wet weight.

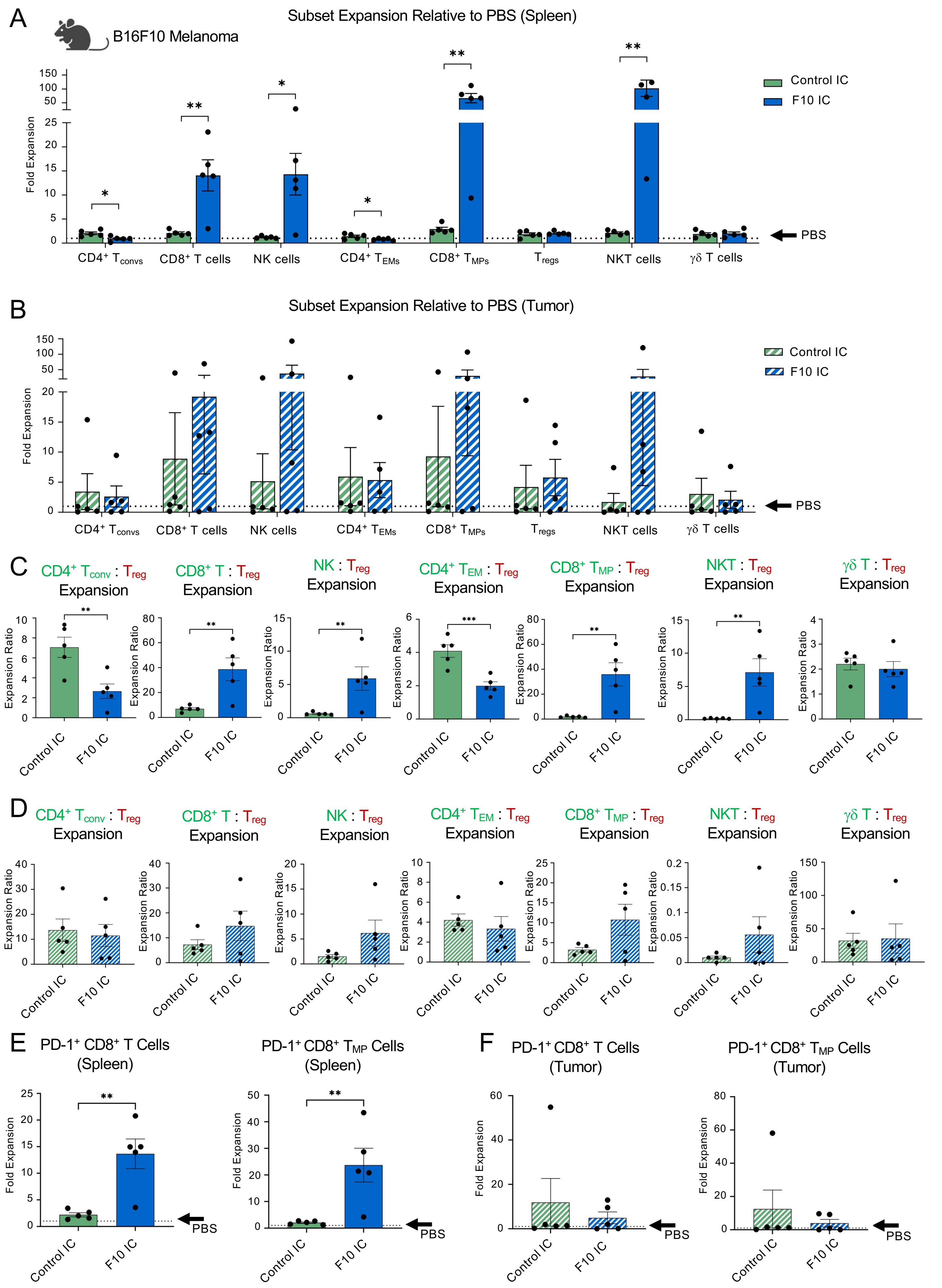

Supplemental Figure 10.

Supplemental Figure 10. Engineered F10 IC promotes late-stage systemic and tumor-localized selective expansion of immune effector cells in B16F10 mouse melanoma model. **(A-F)** C57BL/6 mice were injected subcutaneously with 1×10^5 B16F10 tumor cells and treated intraperitoneally on days 2, 4, 6, and 10 with either PBS or the molar equivalent of 0.125 mg/kg IL-2 of Control IC or F10 IC. Mice were sacrificed on day 21, and spleens and tumors were harvested. Samples from 5 mice were analyzed per group (n=5). The total cell counts of conventional T cells ($CD4^+$ T_{convs}, $CD3^+CD4^+FoxP3^-$), $CD8^+$ T cells ($CD3^+CD4^-CD8^+$), natural killer (NK) cells ($CD3^-CD49b^+CD161^+$), $CD4^+$ effector memory T cells ($CD4^+$ T_{EMs}, $CD3^+CD4^+CD8^-CD44^+CD62L^-$), $CD8^+$ memory phenotype T cells ($CD8^+$ T_{MPs}, $CD3^+CD4^-CD8^+CD44^+CD122^+$), T regulatory cells ($T_{regs}$, $CD3^+CD4^+CD25^+Foxp3^+$), natural killer T (NKT) cells ($CD3^+\gamma\delta TCR^-CD49b^+CD161^+$), and gamma delta ($\gamma\delta$) T cells ($CD3^+\gamma\delta TCR^+$) in each spleen and dissected tumor were determined by flow cytometry. The cell subset counts for each treatment divided by the cell subset counts for PBS are shown for dissected spleen **(A)** and tumors **(B)**. The ratios of each cell subset to T_{regs} in spleen **(C)** and tumors **(D)** for each treatment group are shown. The cell subset counts for PD-1^+CD8^+ T and PD-1^+CD8^+ T_{MP} cells for each treatment group divided by the cell subset counts for PBS are shown for dissected spleen **(E)** and tumors **(F)**. The dotted lines in **(A, B, E,** and **F)** indicate equivalent expansion to PBS treatment. Data are shown as means ± SEM. Statistical significance was determined by one-way ANOVA with Tukey's multiple comparison test: $*P<0.05$, $**P<0.01$, $***P<0.001$.

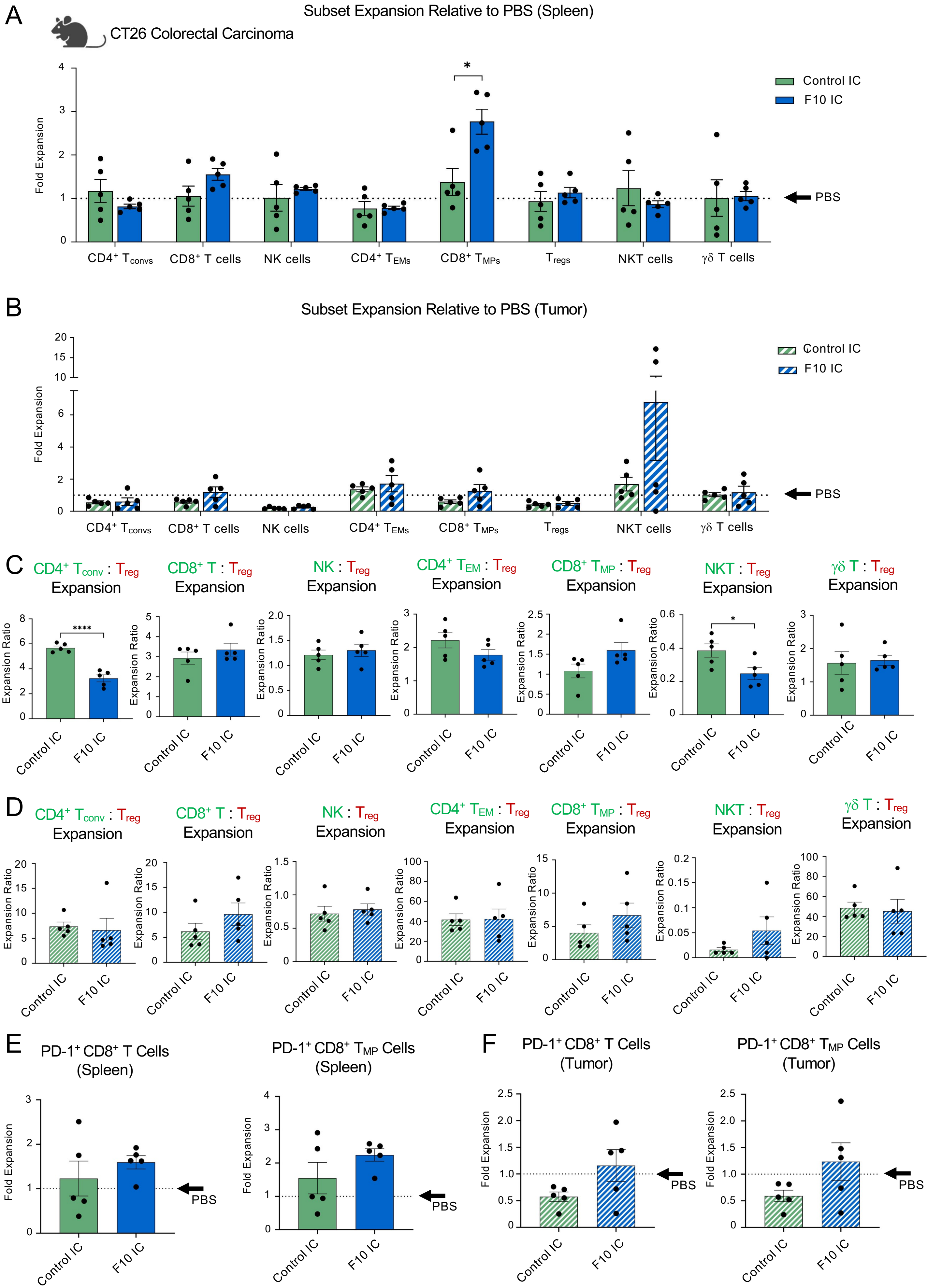

Supplemental Figure 11.

Supplemental Figure 11. Engineered F10 IC promotes late-stage systemic and tumor-localized selective expansion of immune effector cells in CT26 mouse colorectal carcinoma model. **(A-F)** BALB/c mice were injected subcutaneously with 2×10^5 CT26 tumor cells and treated intraperitoneally on days 4, 6, 8, and 12 with either PBS or the molar equivalent of 0.125 mg/kg IL-2 of Control IC or F10 IC. Mice were sacrificed on day 25, and spleens and tumors were harvested. Samples from 5 mice were analyzed per group (n=5). The total cell counts of conventional T cells ($CD4^+$ T_{convs}, $CD3^+CD4^+FoxP3^-$), $CD8^+$ T cells ($CD3^+CD4^-CD8^+$), natural killer (NK) cells ($CD3^-CD49b^+$), $CD4^+$ effector memory T cells ($CD4^+$ T_{EMs}, $CD3^+CD4^+CD8^-CD44^+CD62L^-$), $CD8^+$ memory phenotype T cells ($CD8^+$ T_{MPs}, $CD3^+CD4^-CD8^+CD44^+CD122^+$), T regulatory cells ($T_{regs}$, $CD3^+CD4^+CD25^+Foxp3^+$), natural killer T (NKT) cells ($CD3^+\gamma\delta TCR^-CD49b^+$), and gamma delta (γδ) T cells ($CD3^+\gamma\delta TCR^+$) in each spleen and dissected tumor were determined by flow cytometry. The cell subset counts for each treatment divided by the cell subset counts for PBS are shown for dissected spleen **(A)** and tumors **(B)**. The ratios of each cell subset to T_{regs} in spleen **(C)** and tumors **(D)** for each treatment group are shown. The cell subset counts for PD-1^+CD8^+ T and PD-1^+CD8^+ T_{MP} cells for each treatment group divided by the cell subset counts for PBS are shown for dissected spleen **(E)** and tumors **(F)**. The dotted lines in **(A, B, E, and F)** indicate equivalent expansion to PBS treatment. Data are shown as means ± SEM. Statistical significance was determined by one-way ANOVA with Tukey's multiple comparison test: *P<0.05, **P<0.01, ***P<0.001, ****P<0.0001.

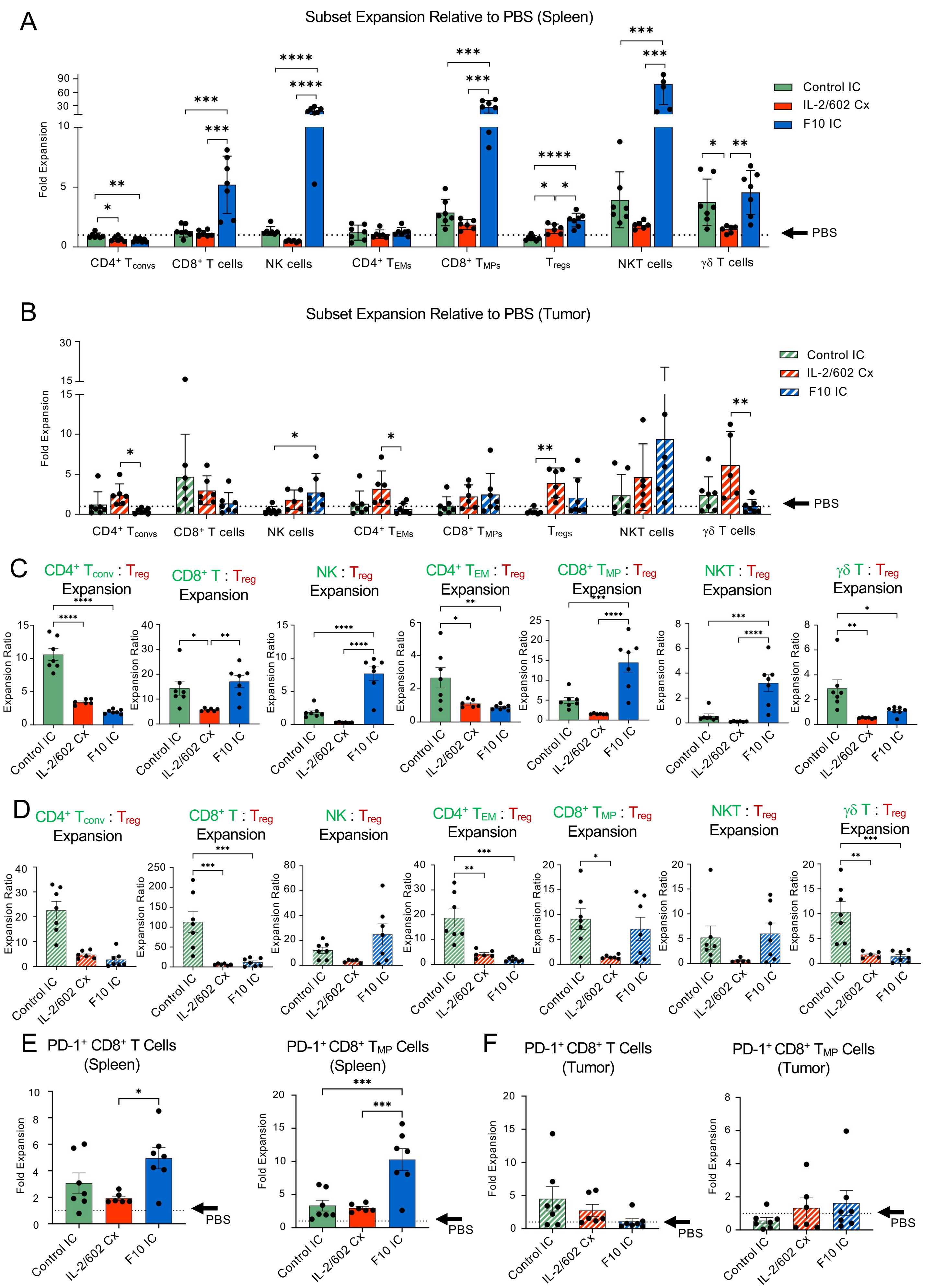

Supplemental Figure 12.

Supplemental Figure 12. Engineered F10 IC promotes early-stage systemic and tumor-localized selective expansion of immune effector cells in B16F10 mouse melanoma model. **(A-F)** C57BL/6 mice (n=6-7) were injected subcutaneously with 1×10^5 B16F10 tumor cells and treated intraperitoneally on days 2, 4, 6, and 10 with either PBS or the molar equivalent of 0.125 mg/kg IL-2 of Control IC, IL-2/602 complex (Cx) (2:1 cytokine:antibody molar ratio), or F10 IC. Mice were sacrificed on day 11, and spleens and tumors were harvested. Samples from 5 mice were analyzed per group (n=5). The total cell counts of conventional T cells ($CD4^+$ T_{convs}, $CD3^+CD4^+FoxP3^-$), $CD8^+$ T cells ($CD3^+CD4^-CD8^+$), natural killer (NK) cells ($CD3^-CD49b^+CD161^+$), $CD4^+$ effector memory T cells ($CD4^+$ T_{EMs}, $CD3^+CD4^+CD8^-CD44^+CD62L^-$), $CD8^+$ memory phenotype T cells ($CD8^+$ T_{MPs}, $CD3^+CD4^-CD8^+CD44^+CD122^+$), T regulatory cells ($T_{regs}$, $CD3^+CD4^+CD25^+Foxp3^+$), natural killer T (NKT) cells ($CD3^+\gamma\delta TCR^-CD49b^+CD161^+$), and gamma delta (γδ) T cells ($CD3^+\gamma\delta TCR^+$) in each spleen and dissected tumor were determined by flow cytometry. The cell subset counts for each treatment divided by the cell subset counts for PBS are shown for dissected spleen **(A)** and tumors **(B)**. The ratios of each cell subset to T_{regs} in spleen **(C)** and tumors **(D)** for each treatment group are shown. The cell subset counts for $PD\text{-}1^+CD8^+$ T and $PD\text{-}1^+CD8^+$ T_{MP} cells for each treatment group divided by the cell subset counts for PBS are shown for dissected spleen **(E)** and tumors **(F)**. The dotted lines in **(A, B, E, and F)** indicate equivalent expansion to PBS treatment. Data are shown as means ± SEM. Statistical significance was determined by one-way ANOVA with Tukey's multiple comparison test: *P<0.05, **P<0.01, ***P<0.001, ****P<0.0001.

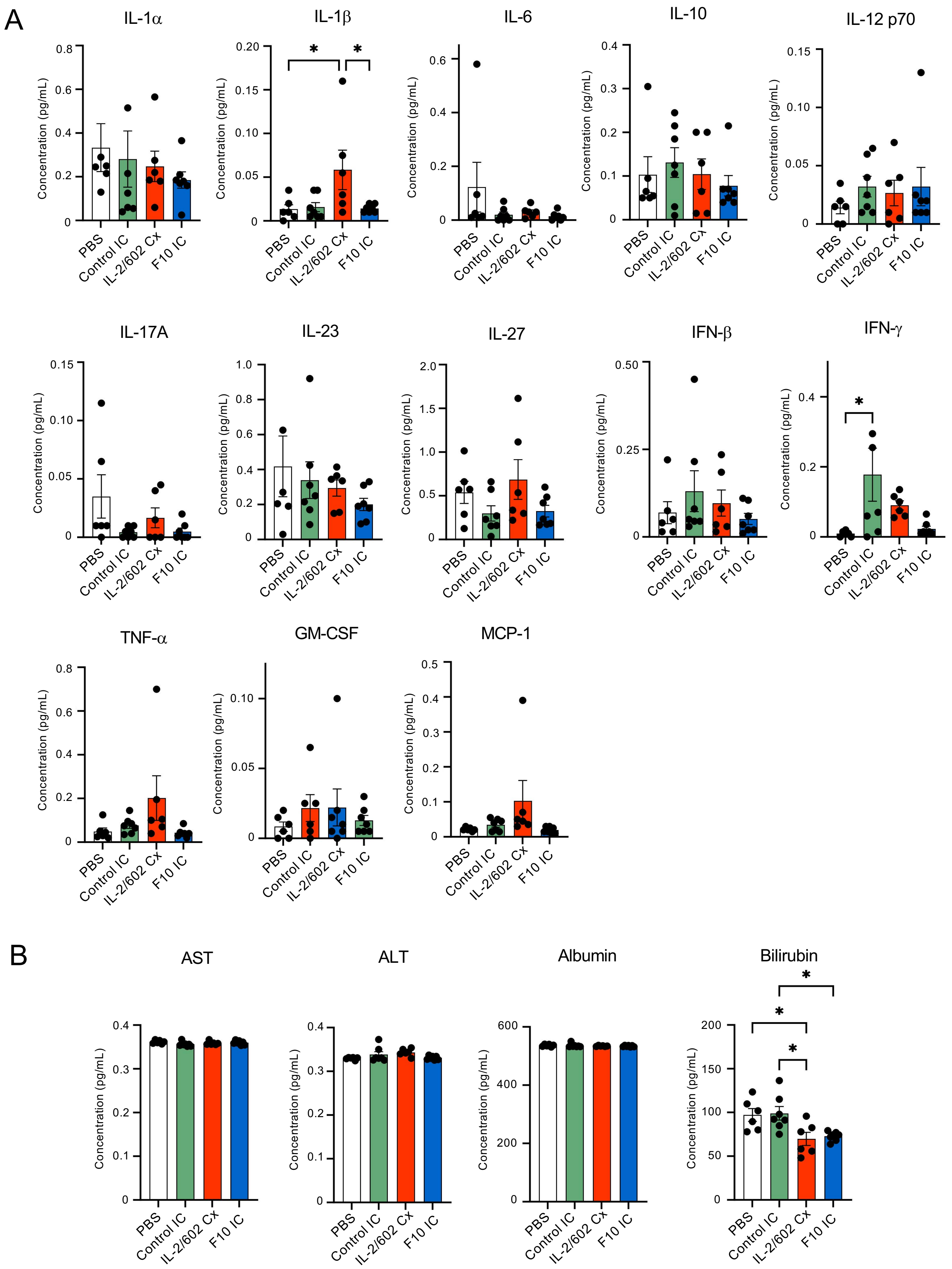

Supplemental Figure 13.

Supplemental Figure 13. Engineered F10 IC does not promote systemic inflammation or liver toxicity in tumor-bearing mice. **(A-B)** C57BL/6 mice (n=6-7) were injected subcutaneously with 1×10^5 B16F10 tumor cells and treated intraperitoneally on days 2, 4, 6, and 10 with either PBS or the molar equivalent of 0.125 mg/kg IL-2 of Control IC, IL-2/602 complex (Cx) (2:1 cytokine:antibody molar ratio) or F10 IC. Mice were sacrificed on day 11, and blood was collected to acquire sera. Serum concentrations of inflammatory cytokines **(A)** and molecules associated with liver toxicity (AST, ALT, albumin, bilirubin) **(B)** were measured. Data are shown as means ± SEM. Statistical significance was determined by one-way ANOVA with Tukey's multiple comparison test: $^*P<0.05$. No significant differences were observed in AST, ALT, or albumin.

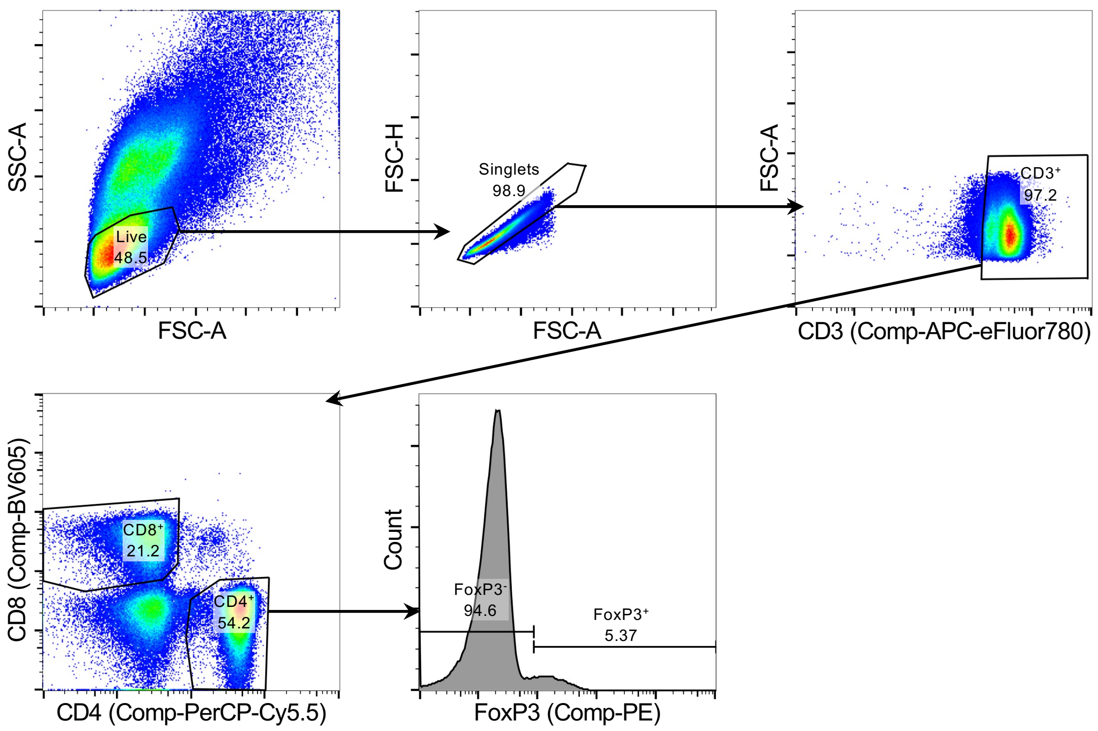

Supplemental Figure 14.

Supplemental Figure 14. Flow cytometry gating strategy for human PBMC signaling assays. $CD4^+$ T_{convs} were gated as Live> Singlets> $CD3^+$> $CD4^+$> $FoxP3^-$, T_{regs} were gated as Live> Singlets> $CD3^+$> $CD4^+$> $FoxP3^+$, and $CD8^+$ T cells were gated as Live> Singlets> $CD3^+$> $CD4^-$ $CD8^+$.

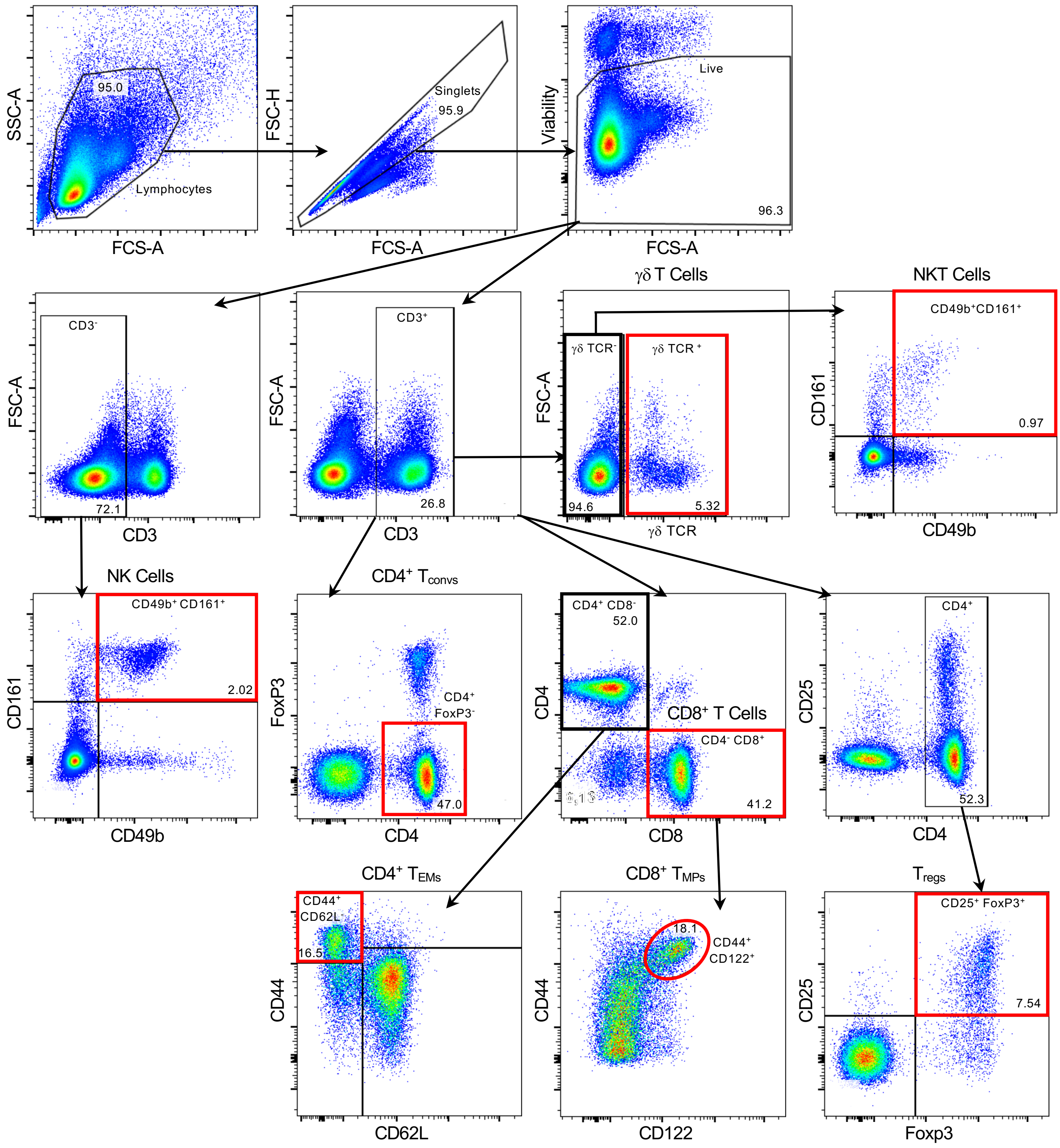

Supplemental Figure 15.

Supplemental Figure 15. Flow cytometry gating strategy for mouse immune cell subset expansion assays. All subsets were derived from the Lymphocytes> Singlets> Live gate. Within this gate, NK cells were gated as $CD3^{-}$> NK1.1^{+} CD49b^{+}, CD4^{+} T_{convs} were gated as $CD3^{+}$> CD4^{+}FoxP3^{-}, CD4^{+} T_{EMs} were gated as $CD3^{+}$> CD4^{+}CD8^{-}> CD44^{+}CD62L^{-}, CD8^{+} T cells were gated as $CD3^{+}$> CD4^{-}CD8^{+}, CD8^{+} T_{MPs} were gated as $CD3^{+}$> CD4^{-}CD8^{+}> CD44^{+}CD122^{+}, NKT cells were gated as $CD3^{+}$>γδTCR^{-}>CD49b^{+}CD161^{+}, γδ T cells were gated as $CD3^{+}$>γδTCR^{+} and T_{regs} were gated as $CD3^{+}$> CD4^{+}> CD25^{+}FoxP3^{+}.

Supplemental Table 1. IC variant and IL-2, IL-2Rα and IL-2Rβ binding properties.

Immobilized	Soluble	Kinetic Fit Values			Equilibrium
		K_D (nM)	k_{on} ($\times 10^4$ 1/Ms)	k_{off} ($\times 10^{-4}$ 1/s)	K_D (nM)
hIL-2	IL-2	ND	ND	ND	>2000
	Control IC LN35	ND	ND	ND	>2000
	IL-2/602 Cx	10.6 ± 0.1	3.41 ± 0.01	3.62 ± 0.03	103 ± 35
	602 IC LN15	88.3 ± 2.5	0.500 ± 0.005	4.41 ± 0.12	~905
	602 IC LN25	76.9 ± 2.4	0.448 ± 0.003	3.44 ± 0.10	941 ± 134
	602 IC LN35	143.2 ± 2.9	0.265 ± 0.002	3.79 ± 0.07	>2000
	F10 IC LN35	166.9 ± 8.6	0.240 ± 0.004	4.00 ± 0.19	>2000
	602 Ab	0.13 ± 0.02	38.2 ± 0.3	0.51 ± 0.09	6.3 ± 5.2
	Irrel. Ab	ND	ND	ND	>2000
hIL-2Rα	IL-2	3.20 ± 0.08	201.2 ± 3.9	34.4 ± 0.3	7.3 ± 14.5
	Control IC LN35	ND	ND	ND	5.2 ± 8.2
	IL-2/602 Cx	0.55 ± 0.09	42.1 ± 1.0	2.3 ± 0.4	>2000
	602 IC LN15	ND	ND	ND	>2000
	602 IC LN25	ND	ND	ND	>2000
	602 IC LN35	ND	ND	ND	>2000
	F10 IC LN35	ND	ND	ND	>2000
	602 Ab	ND	ND	ND	>2000
	Irrel. Ab	ND	ND	ND	>2000
hIL-2Rβ	IL-2	ND	ND	ND	112 ± 303
	Control IC LN35	0.18 ± 0.01	1554 ± 35	3.76 ± 0.15	4.3 ± 6.3
	IL-2/602 Cx	3.89 ± 0.04	41.0 ± 0.3	15.5 ± 0.1	5.2 ± 5.6
	602 IC LN15	2.20 ± 0.02	24.2 ± 0.1	5.11 ± 0.05	8.4 ± 1.9
	602 IC LN25	3.66 ± 0.03	27.6 ± 0.1	9.98 ± 0.07	5.3 ± 6.4
	602 IC LN35	4.22 ± 0.04	34.2 ± 0.2	14.2 ± 0.1	5.0 ± 5.9
	F10 IC LN35	4.16 ± 0.04	31.4 ± 0.2	12.9 ± 0.1	5.3 ± 5.2
	602 Ab	ND	ND	ND	>2000
	Irrel. Ab	ND	ND	ND	>2000
mIL-2Rα	F10 IC LN35	ND	ND	ND	ND
	Control IC LN35	4.74 ± 0.06	15.8 ± 0.11	6.94 ± 0.07	12.0 ± 2.9
	602 Ab	ND	ND	ND	>10000
mIL-2Rβ	F10 IC LN35	6.46 ± 0.36	4.089 ± 0.05	3.78 ± 0.11	132.3 ± 20.94
	Control IC LN35	>10000	0.48 ± 0.6	5125 ± 392	>10000
	602 Ab	ND	ND	ND	>10000

Supplemental Table 2. T_m values of IL-2, IL-2/602 Cx, 602 Ab, 602 IC and F10 IC.

Sample	T_m (°C)
	HBS
IL-2	61.3*
602 Ab	73.1
IL-2/602 Cx	74.2
602 IC	74.9
F10 IC	76.5

* *Value is for* T_m1

Supplemental Table 3. EC_{50} values for 602 IC linker length variants signaling on IL-2Rα$^+$ or IL-2Rα$^-$ YT-1 cells.

Treatment	EC_{50} (nM)	
	IL-2Rα$^+$	IL-2Rα$^-$
IL-2	0.11 ± 0.35	0.34 ± 0.10
IL-2/602 Cx	0.25 ± 0.15	0.81 ± 1.18
602 IC LN15	38 ± 16	78 ± 67
602 IC LN25	5.1 ± 4.5	2.8 ± 1.4
602 IC LN35	2.3 ± 1.0	0.65 ± 1.44

Supplemental Table 4. EP602 variants and IL-2 and IL-2Rβ binding properties.

Immobilized	Soluble	Kinetic Fit Values			Equilibrium
		K_D (nM)	k_{on} ($\times 10^4$ 1/Ms)	k_{off} ($\times 10^{-4}$ 1/s)	K_D (nM)
IL-2	IL-2	ND	ND	ND	>2000
	IL-2/602 Cx	13.27 ± 0.09	2.52 ± 0.01	3.06 ± 0.02	74 ± 20
	602 IC	ND	ND	ND	>2000
	A8 IC	ND	ND	ND	>2000
	C5 IC	ND	ND	ND	>2000
	E10 IC	20.3 ± 1.8	0.192 ± 0.002	0.39 ± 0.03	>2000
	F10 IC	ND	ND	ND	>2000
	H2 IC	50.5 ± 2.5	0.221 ± 0.002	1.11 ± 0.05	>2000
	602 Ab	0.044 ± 0.05	64.2 ± 0.3	0.28 ± 0.03	3.7 ± 7.4
	Irrel. Ab	ND	ND	ND	>2000
IL-2Rβ	IL-2	ND	ND	ND	161 ± 28
	IL-2/602 Cx	0.30 ± 0.02	38.7 ± 1.8	1.17 ± 0.04	6.0 ± 2.6
	602 IC	0.26 ± 0.04	28.8 ± 2.8	0.75 ± 0.07	5.4 ± 1.2
	A8 IC	0.52 ± 0.01	52.6 ± 1.0	2.74 ± 0.02	4.2 ± 1.1
	C5 IC	2.38 ± 0.04	48.7 ± 0.7	11.57 ± 0.12	11 ± 12
	E10 IC	0.84 ± 0.04	26.2 ± 1.3	2.20 ± 0.03	4.9 ± 0.7
	F10 IC	1.45 ± 0.23	9.7 ± 1.5	1.41 ± 0.04	4.4 ± 1.2
	H2 IC	5.1 ± 1.2	6.0 ± 1.4	3.03 ± 0.04	4.3 ± 0.7
	602 Ab	ND	ND	ND	>2000
	Irrel. Ab	ND	ND	ND	>2000

Supplemental Table 5. IL-2 binding properties of F10 and 602.

Immobilized	Soluble	Kinetic Fit Values			Equilibrium
		K_D (nM)	k_{on} (×10^5 1/Ms)	k_{off} (×10^{-4} 1/s)	K_D (nM)
IL-2	602 scFv	1.94 ± 0.02	3.61 ± 0.04	6.70 ± 0.04	9.00 ± 8.59
	F10 scFv	0.34 ± 0.01	5.63 ± 0.03	1.88 ± 0.04	6.12 ± 5.66
	Irrel. scFv	ND	ND	ND	>2000
	IL-2/602 Cx	ND	ND	ND	168 ± 237
	IL-2/F10 Cx	ND	ND	ND	>2000
	602 IC	ND	ND	ND	>2000
	F10 IC	ND	ND	ND	>2000
	Control IC	ND	ND	ND	>2000

Supplemental Table 6. EC50 values for IC variant signaling on IL-2Rα$^+$ or IL-2Rα$^-$ YT-1 cells, and various PBMC subsets.

Treatment	EC_{50} (nM)				
	IL-2Rα$^+$ YT-1	IL-2Rα$^-$ YT-1	CD3$^+$CD4$^+$ CD8$^-$ FoxP3$^+$ PBMC (T_{regs})	CD3$^+$CD4$^+$ CD8$^-$ FoxP3$^-$ PBMC (T_{convs})	CD3$^+$CD4$^-$ CD8$^+$ PBMC (CD8$^+$ T)
IL-2	0.09 ± 0.07	0.36 ± 0.06	3.7E-4 ± 1.54E-3	0.051 ± 0.044	0.71 ± 1.00
IL-2/602 Cx	0.03 ± 0.02	0.41 ± 0.09	2.5E-4 ± 2.9E-4	0.024 ± 0.036	1.63 ± 4.59
602 IC	0.17 ± 0.13	0.44 ± 0.11	1.1E-3 ± 2.0E-3	0.13 ± 0. 46	2.1 ± 1.8
F10 IC	1.30 ± 0.69	0.49 ± 0.24	1.17 ± 1.11	26 ± 12	10.6 ± 4.1
Control IC	1.28 ± 0.50	0.46 ± 0.14	3.6 ± 3.7	42 ± 27	14.5 ± 6.5

Supplemental Table 7. Crystallographic statistics for IL-2+602 scFv and IL-2/F10 scFv structures.

	IL-2/602 scFv	IL-2/F10 scFv
Data collection		
Space group	C 1 2 1	C 1 2 1
a, *b*, *c* (Å)	137.19, 36.42, 64.19	136.05, 40.22, 68.12
α, β, γ (°)	90.00, 108.12, 90.00	90.00, 111.21, 90.00
Resolution (Å)	61.00 – 1.64	38.45-1.71
No. reflections (total)	72451 (6877)	254475 (24961)
No. reflections (unique)	37048 (3555)	37142 (3657)
Rmerge	0.03044 (0.6296)	0.07237 (0.968)
$I/\sigma I$	5.97 (1.01)	12.68 (1.69)
Completeness (%)	99.1 (96.9)	98.74 (98.33)
Redundancy	3.7 (3.9)	6.9 (6.8)
Refinement		
Resolution (Å)	32.60-1.64 (1.69-1.64)	38.45-1.71
Reflections used in refinement	37047 (3555)	37085 (3653)
Reflections used for R-free	1824 (173)	1996 (196)
R_{work} / R_{free}	0.219 / 0.250 (0.370 / 0.401)	0.216 / 0.235 (0.314 / 0.329)
No. atoms		
Protein	2615	2620
Water	155	215
B-factors		
Protein	37.03	37.57
Water	40.66	40.06
R.m.s. deviations		
Bond lengths (Å)	0.01	0.01
Bond angles (°)	1.19	1.31

*Values in parenthesis are for the highest resolution shell

SUPPLEMENTAL METHODS

Cell lines

Human embryonic kidney (HEK) 293F (Thermo Life Sciences) cells were cultivated in Freestyle 293 Expression Medium (Thermo Life Technologies), supplemented with 10 U/mL penicillin-streptomycin (Gibco). Unmodified YT-1 (1) and IL-2Rα^+ YT-1 (2) human natural killer (NK) cells were obtained with permission from Dr. Junji Yodoi and cultured in RPMI complete medium (RPMI 1640 medium supplemented with 10% fetal bovine serum, 2 mM L-glutamine, minimum non-essential amino acids, sodium pyruvate, 25 mM HEPES, and penicillin-streptomycin [all reagents from Gibco]). CT26 (ATCC) cells were maintained in RPMI-1640 (Sigma) with 10% FBS and 100 U/mL penicillin-streptomycin (Gibco). B16F10 (ATCC) cells were maintained in and DMEM high glucose medium (Sigma) supplemented with 10% FBS and 100 U/mL penicillin-streptomycin (Gibco). All cell lines were maintained at 37ºC in a humidified atmosphere with 5% CO_2. HEK 293F cells were maintained on a shaker set to 125 rpm.

Protein expression and purification

The variable heavy (V_H) and variable light (V_L) chain sequences of the 602 antibody were determined by PCR amplification from 602 antibody-expressing hybridoma cells. cDNA encoding the V_H and V_L of MAB602 was obtained by performing reverse-transcription-polymerase chain reaction (RT-PCR) on mRNA isolated from MAB602 hybridoma cells. V_H cDNA was digested with the HindIII and KpnI restriction enzymes and ligated into the pSecTaqA vector; V_L cDNA was digested with the BamHI and XhoI restriction enzymes and ligated into the pcDNA 3.1^{Hygro} vector. Sequencing of the V_H and V_L was performed using the Sanger method. Recombinant antibodies were formulated as mouse immunoglobulin (IgG) 2a kappa isotype to match the parent clone. The heavy chain (HC) and light chain (LC) of the 602 antibody were separately cloned into the gWiz vector (Genlantis) (3). For wild type 602 immunocytokines (ICs) and variants thereof, the human IL-2 cytokine (amino acids 1–133) was fused to the full-length 602 antibody at the N-terminus of the LC by a flexible $(G_4S)_N$ linker (N=2, 3, 5, or 7; Figure 1). The control IC was comprised of hIL-2 fused to the N-terminus of the LC of an irrelevant anti-fluorescein antibody, 4-4-20 (4), with identical isotype to that of 602. For effector function knockout (ΔFc) ICs, mutations L234A, L235A, and P329G (5) were introduced to the heavy chain of the antibody.

Antibodies and ICs were expressed recombinantly in human embryonic kidney (HEK) 293F cells via transient co-transfection of plasmids encoding the HC and either LC or an IL-2/LC fusion protein. HC and LC plasmids were titrated in small-scale co-transfection tests to determine optimal DNA content ratios for large-scale expression. HEK 293F cells were grown to 1.2×10^6 cells/mL and diluted to 1.0×10^6 cells/mL on the day of transfection. Plasmid DNA and polyethyleneimine (PEI, Polysciences) were independently diluted to 0.05 and 0.1 mg/mL, respectively, in OptiPro medium (Thermo Life Technologies) and incubated at room temperature for 15 min. Equal volumes of diluted DNA and PEI were mixed and incubated at room temperature for an additional 15 min. Subsequently, the DNA/PEI mixture (40 mL per liter cells) was added to a flask containing the diluted cells, which was then incubated at 37ºC with shaking for 5 days. Secreted protein was

harvested from HEK 293F cell supernatants by Protein G affinity chromatography, followed by size-exclusion chromatography on an ÄKTA™ fast protein liquid chromatography (FPLC) instrument using a Superdex 200 column (Cytiva). Analysis of the relative distribution of IC eluted in each peak was performed using UNICORN analysis software v7.1 (Cytiva). Statistical analysis was conducted using GraphPad Prism data analysis software v9.0.

Human IL-2 (amino acids 1–133), IL-2Rα ectodomain (amino acids 1–217), IL-2Rβ ectodomain (amino acids 1–214), 602 scFv (VH-$[G_4S]_3$-VL), and F10 scFv (VH-$[G_4S]_3$-VL) each contained a C-terminal hexahistidine tag, and were produced from HEK 293F cells, as described for the 602 antibody and ICs. Proteins were purified via Ni-NTA (Expedeon) affinity chromatography followed by size-exclusion chromatography on a Superdex 200 column (Cytiva) using an FPLC instrument. All proteins were stored in HEPES-buffered saline (HBS, 150 mM NaCl in 10 mM HEPES pH 7.3). Purity was verified by SDS-PAGE analysis.

For preparation of biotinylated IL-2, IL-2Rα, and IL-2Rβ, a C-terminal biotin acceptor peptide (BAP) GLNDIFEAQKIEWHE sequence was included. Following Ni-NTA affinity chromatography, the cytokine and receptors were biotinylated with the soluble BirA ligase enzyme in 0.5 mM Bicine pH 8.3, 100 mM ATP, 100 mM magnesium acetate, and 500 mM biotin (Sigma). After overnight incubation at 4ºC, excess biotin was removed by size-exclusion chromatography on an ÄKTA™ FPLC instrument using a Superdex 200 column (Cytiva).

Yeast surface binding studies

General yeast display methodologies were carried out with EBY-100 yeast cells, as described previously (6, 7). The V_H chain followed by the V_L chain of the 602 antibody, separated by a $(G_4S)_3$ linker (scFv format), were cloned into the yeast display vector pCT3CBN (containing the yeast agglutinin protein Aga2 N-terminal to the scFv and a C-terminal c-Myc detection tag). Yeast displaying scFvs were plated at 2×10^5 per well in a 96-well plate and incubated in PBE buffer (phosphate-buffered saline [PBS] pH 7.2 with 0.1% bovine serum albumin [BSA] and 1 mM ethylenediaminetetraacetic acid [EDTA]) containing biotinylated IL-2 in the presence or absence of IL-2Rα for 2 hr at room temperature. Cells were then washed and stained with a 1:200 dilution of Alexa Fluor 647-labeled streptavidin (Thermo Fisher Scientific) in PBSA for 15 minutes at 4ºC. After a final wash, cells were analyzed for target binding using a CytoFLEX flow cytometer (Beckman Coulter). Background-subtracted and normalized binding curves were fitted to a first-order binding model and equilibrium dissociation constant (K_D) values were determined using GraphPad Prism software v9.0. Experiments were conducted at least twice with consistent results.

Generation of a mutagenic yeast-displayed library of 602 scFv variants

A targeted error-prone library that mutagenized the CDR1 and CDR3 of both the heavy and light chain variable domains was generated to preserve existing IL-2 interactions while also allowing for potentially beneficial, conservative alterations in binding. The 4 targeted CDRs were amplified by error-prone PCR using Taq polymerase (New England Biolabs), 1× Taq buffer (New England Biolabs), 2 mM manganese(II) chloride, 7 mM magnesium chloride, 0.2 mM of dATP

and dGTP, 1mM dCTP and dTTP, 0.5 μM of each primer, and 0.2 ng/μL of the template. Following 5 amplification cycles, the PCR mix was transferred and diluted 1:5 into fresh mix lacking the template. The amplification, transfer, and dilution steps were repeated twice more, with the final transfer undergoing 20 total amplification cycles. The 5 framework sequences adjacent to the targeted CDRs were amplified using Phusion High-Fidelity DNA polymerase (Thermo Scientific). Framework fragments were assembled with the neighboring mutagenized CDR fragments by sequential, pairwise overlap extension PCR using Phusion polymerase. The final assembled fragments contained the full 602 scFv as well as homologous sequences (≥ 97nt) on both ends, which provided overlap with the cut yeast display vector, pCT3CBN.

EBY100 yeast were transformed with the cut pCT3CBN vector and mutagenized fragment (approximately 10 μg and 50 μg, respectively), which were assembled by homologous recombination, as previously described (6, 7). The library yielded 1.2×10^8 transformants, which were grown in SDCAA media (pH 4.5) for 48 hr prior to passaging, and were then induced 24 hr later in SGCAA media (pH 4.5) at an OD of 1. Individual yeast plasmids were extracted via miniprep (Zymo Research) to verify proper insertion of the fragment into the vector backbone. A productive error rate—i.e., DNA mutations that resulted in changes to the amino acid sequence—of approximately 6% was observed.

Mutagenic 602 scFv Library Selections

Yeast selection methodologies were modified from previously described protocols (6, 7). Each round of selection was performed with sufficient yeast to ensure 10-fold coverage of the library diversity. Yeast clones selected from each round were grown overnight at 30˚C in SDCAA media for 2 days, followed by induction in SGCAA media for 2 days at 20˚C.

In the first round of selection, the naïve mutagenic 602 scFv library was debulked via magnetic-activated cell sorting (MACS). A pre-clearing step was performed to eliminate variants that bound to Alexa Fluor 647-conjugated streptavidin (SA-AF647) (Thermo Fisher Scientific), and the library was then positively selected for variants that bound biotinylated IL-2 tetramerized with SA-AF647. All staining was performed in PBE solution. In the first MACS step, the yeast were incubated with 20 μg/mL SA-AF647 for 1 hr at 4°C, washed, and then incubated with 1:20 anti-Cy5/anti-Alexa Fluor 647 microbeads (Miltenyi Biotec) for 20 minutes at 4°C, washed, and then applied to an LS MACS separation column (Miltenyi Biotec), according to the manufacturer's protocol. Yeast that flowed through the column (i.e., did not bind to SA-AF647 alone) were collected and prepared for the second MACS step. Biotinylated IL-2 was mixed with SA-AF647 in a 4:1 molar ratio, diluted in PBE, and incubated for 15 minutes to form tetramers. Yeast were incubated with 2 nM IL-2 tetramers for 2 hr at 4˚C, then washed and incubated with anti-Cy5/anti-Alexa Fluor 647 microbeads as before. Yeast were applied to an LS MACS separation column, and yeast clones eluted from the column were collected, grown, and induced for the next round of selection.

A second round of selection isolated full-length 602 scFv variants (based on c-Myc tag expression) via MACS. Yeast were incubated with a 1:100 dilution of AF647-conjugated anti-c-

Myc antibody (clone 9B11, Cell Signaling Technologies) in PBE for 2 hr at 4˚C, incubated with anti-Cy5/anti-Alexa Fluor 647 microbeads, and applied to a LS MACS separation column. Eluted cells were collected, regrown, and induced.

The final three rounds of selection were performed by fluorescence-activated cell sorting (FACS) on a FACSymphony S6 cell sorter (Becton Dickinson), using decreasing amounts of biotinylated IL-2 in the presence of a large excess of nonbiotinylated IL-2Rα to select for the variants that competitively blocked IL-2 interaction with the IL-2Rα subunit. The first of these competitive FACS selections used 50 nM IL-2 with 1.5 μM IL-2Rα, and the second and third both used 30 nM IL-2 and 0.3 μM IL-2Rα. The third sort selected for variants with low off-rates by first incubating the library with IL-2, washing, and then incubating with IL-2Rα at room temperature for 2 hr to allow for IL-2 dissociation. In all FACS selections, variants in the top 5% with respect to IL-2 binding were collected.

Bio-layer interferometry binding studies

Biotinylated human IL-2, IL-2Rα and IL-2Rβ and biotinylated mouse IL-2Rα and IL-2Rβ were immobilized to streptavidin-coated tips for analysis on an Octet Red96 bio-layer interferometry (BLI) instrument (Sartorius). Less than 5 signal units (nm) of IL-2 or each receptor subunit was immobilized to minimize mass transfer effects. PBSA (PBS pH 7.2 containing 0.1% BSA) was used for all dilutions and as dissociation buffer. Tips were exposed to serial dilutions of IL-2, 602 antibody, IL-2/602 complexes (formed by incubating a 1:1 molar ratio of IL-2 and 602 antibody for 30 minutes at room temperature), various ICs, or an irrelevant protein (the human monoclonal antibody trastuzumab) in a 96-well plate for 300 s. Dissociation was then measured for at least 240 s. Surface regeneration for all interactions was conducted using 15 s exposure to 0.1 M glycine pH 3.0. Analysis and kinetic curve fitting (assuming a 1:1 binding model) were conducted using Octet Data Analysis HT software version 7.1 (Sartorius). Normalized equilibrium binding curves were obtained by plotting the response value at the end of the association phase for each sample dilution and normalizing to the maximum value. Equilibrium binding curves were fitted and K_D values determined using GraphPad Prism data analysis software v10.2 or earlier, assuming all binding interactions to be first order. Experiments were performed at least twice with similar results.

Analytical Ultracentrifugation

Sedimentation analysis was performed using a ProteomeLab XL-I analytical ultracentrifuge (Beckman Coulter) (8). Protein samples were diluted in a buffer containing 10 mM HEPES, 150 mM NaCl, pH 7.5 before the analysis; the same buffer was used as a reference. IL-2/602 complex was prepared in the same buffer by incubating IL-2 and 602 Ab at a 2:1 molar ratio for 15 min at room temperature. The sedimentation velocity experiment was conducted at 36,000-48,000 rpm and 20˚C using double sector cells and an An50-Ti rotor at 0.1–0.6 mg/ml protein loading concentration. In total, 100-300 absorbance scans were recorded at 230 or 280 nm at 2-6 min intervals. Buffer density, protein partial specific volumes, particle dimensions, and the values of

$s_{20,w}$ sedimentation coefficients corrected to standard conditions (corrected to 20˚C and the density of water) were estimated in Sednterp (9). Data were analyzed with Sedfit (10) using the c(s) continuous sedimentation coefficient distribution model, and the figures were prepared in GUSSI (11).

Crystallographic characterization of IL-2 in complex with 602 or F10 scFv

Complexation of IL-2 with the F10 or 602 scFv was carried out overnight in the presence of carboxypeptidases A and B at 4°C. The complex was re-purified using SEC and concentrated using Amicon 30 kDa MWCO products. Screening for crystallization of the F10 scFv/IL-2 complex was carried out using commercial sparse matrices. Narrowed screens for the F10 complexes were made based on Nextal PEGs Suite condition E3, and crystals were grown using the hanging drop method in a range of conditions containing between 15-30% PEG3350 w/v and between 100-350 mM ammonium fluoride. Crystallization of the 602 complexes was carried out in the same range of conditions. Crystals presented a variety of plate morphologies. For F10, data collection was carried out at Stanford Synchrotron Radiation Lightsource (SSRL) beamline BL12-1. For 602, collection was carried out at Brookhaven National Laboratory (BNL) beamline 17-ID-1. In both cases, initial indexing, scaling, and merging was carried out using autoproc. For both structures, model building was carried out in the PHENIX suite, using Phaser MR for molecular replacement with search models derived from PDB 2B5I and PDB 7RK2, both modified in Sculptor. Model building was carried out using AutoBuild and phenix.refine, with further manual refinement performed in WinCoot. Alignments and visual representations were carried out using PyMol. Interface characterizations were carried out using PDBePISA.

Thermostability comparisons by differential scanning fluorometry

Thermal unfolding of IL-2, 602 Ab, IL-2/602 complex (molar ratio of 2:1 of IL-2 to 602 antibody), 602 IC and F10 IC was measured as change in intrinsic tryptophan fluorescence as a function of temperature via nano differential scanning fluorometry (nanoDSF). The samples (10 µL at 0.2 mg/mL in HBS) were loaded into standard treated NT.115 capillaries (NanoTemper), and absorption at 330 and 350 nm was measured on a Prometheus NT.48 instrument (NanoTemper) in the temperature range 30-95°C with a temperature slope of 1.5°C/min. The melting curves show the ratio of the absorbances at 330 nm over 350 nm versus temperature, and the melting temperatures (T_m) were calculated in PR.ThermControl (ver. 2.1.1) software from the first derivative of the melting curves.

YT-1 cell STAT5 phosphorylation studies

Approximately 2×10^5 IL-2Rα^- (1) or IL-2Rα^+ (2) YT-1 human NK cells were plated in each well of a 96-well plate. Cells were washed and resuspended in RPMI complete medium containing serial dilutions of IL-2, IL-2/602 complexes (formed by pre-incubating a 1:1 molar ratio of IL-2 and 602 antibody for 30 minutes at room temperature), or various ICs. Cells were incubated with treatments for 20 min at 37ºC and immediately fixed by addition of paraformaldehyde (Electron Microscopy Sciences) to 3.7%, followed by 10 min incubation at room temperature. Cells were

permeabilized by resuspension in ice-cold 100% methanol and overnight incubation at -80°C. Fixed and permeabilized cells were washed twice with PBSA buffer and incubated for 2 hr at room temperature with Alexa Fluor 647-conjugated anti-STAT5 pY694 antibody (BD Biosciences, clone 47/Stat5) diluted 1:50 in PBSA buffer. Cells were then washed with PBSA buffer and analyzed on a CytoFLEX flow cytometer (Beckman Coulter). Normalized mean fluorescence intensity (MFI) was calculated by subtracting the MFI of unstimulated cells and normalizing to the maximum signal intensity. Dose-response curves were fitted to a logistic model and half-maximal effective concentrations (EC_{50}) were calculated using GraphPad Prism data analysis software v9.0. Experiments were conducted in triplicate and performed 3 times with similar results.

To compare the signaling for the selected 602 IC LN35 variants derived from library selections, mixed IL-2Rα^-/IL-2Rα^+ YT-1 cell assays were performed in which IL-2Rα^- YT-1 cells were lentivirally transduced such that 4% of cells were IL-2Rα^+. For lentiviral transduction, full-length human IL-2Rα was cloned into the pCDH-CMV-MCS-EF1-Puro expression vector, and the pPACKH1 HIV Lentivector Packaging Kit (System Biosciences) was used to produce lentivirus particles, per manufacturer instructions. Approximately 3.5×10^6 YT-1 cells were transduced by centrifugal inoculation (30 min at 800×g) in RPMI complete medium containing polybrene (8 μg/mL) and 15% harvested lentivirus-containing medium. For STAT5 phosphorylation studies, at least 420 IL-2Rα^+ events per sample were collected via flow cytometry. Experiments were conducted in triplicate and performed at least three times with similar results.

Human PBMC isolation and STAT5 phosphorylation studies

Leukopaks containing de-identified human whole blood were obtained from Anne Arundel Medical Blood Donor Center (Anne Arundel, Maryland, USA). Peripheral blood mononuclear cells (PBMCs) were isolated via Ficoll-Paque Plus (GE Healthcare) gradient, per manufacturer recommendations, followed by incubation with ACK lysis buffer (Gibco) for removal of red blood cells.

Approximately 2×10^6 PBMCs were plated in each well of a 96-well plate immediately following isolation. Greater than 99% viability was confirmed by staining with LIVE/DEAD Fixable Aqua dead cell stain (Invitrogen) diluted 1:1000 in PBS for 15 minutes at 4°C. Cells were resuspended in RPMI complete medium containing serial dilutions of IL-2, IL-2/602 complexes (formed by pre-incubating a 1:1 molar ratio of IL-2 and 602 antibody for 30 minutes at room temperature), or various ICs. Cells were incubated with treatments for 20 min at 37ºC, and immediately fixed and permeabilized with Transcription Factor Phospho Buffer kit (BD Pharmingen), per manufacturer recommendations. Cells were then stained for 2 hr at room temperature with the following antibodies at the manufacturer-recommended dilutions in 1× TFP Perm/Wash buffer: APC-eFluor780 anti-human CD3 (clone UCHT1, Invitrogen); PerCP-Cy5.5 anti-human CD4 (clone SK3, BD Pharmingen); BV605 anti-human CD8 (clone SK1, Biolegend); BV421 anti-human CD25 (clone M-A251, BD Horizon); Alexa Fluor 488 anti-human CD127 (clone eBioRDR5, Invitrogen); PE anti-human FoxP3 (clone 236A/E7, BD Pharmingen); and

Alexa Fluor 647 anti-STAT5 pY694 (clone 47/Stat5, BD Biosciences). Cells were washed, resuspended in PBSA buffer, and analyzed on a CytoFLEX flow cytometer (Beckman Coulter). At least 1500 T_{reg} cell events ($CD3^{+}CD4^{+}IL\text{-}2R\alpha^{+}Foxp3^{+}$) were collected in each sample.

Data were analyzed using FlowJo software v10.6.1 (Tree Star). Normalized pSTAT5 MFI for T_{regs} ($CD3^{+}CD4^{+}Foxp3^{+}$), $CD4^{+}$ T_{convs} ($CD3^{+}CD4^{+}Foxp3^{-}$), and $CD8^{+}$ T cells ($CD3^{+}CD8^{+}$) were calculated by subtracting the MFI of unstimulated cells and normalizing to the maximum signal intensity. An example of the gating strategy used for these experiments is depicted in Supplemental Figure 14. Dose-response curves were fitted to a logistic model, and half-maximal effective concentrations (EC_{50}) were calculated using GraphPad Prism data analysis software v9.0. Experiments were conducted in triplicate and performed at least twice using independent donors with similar results.

RNA sequencing analysis of IL-2 and IC stimulated primary human CD8⁺ T cells

Human $CD8^{+}$ T cells were purified from buffy coats from healthy donors at the NIH Blood Bank using EasySep™ Human $CD8^{+}$ T Cell Isolation Kit (StemCell Technologies). Approximately 2×10^{4} $CD8^{+}$ T cells were plated in each well of a 96-well plate immediately following isolation. Cells were incubated with treatments for 20 min at 37ºC, immediately fixed and stained for phospho-STAT5 as described for the YT-1 cells, and analyzed on a FACSCanto II flow cytometer (BD). Normalized mean fluorescence intensity (MFI) was calculated by subtracting the MFI of unstimulated cells and normalizing to the maximum signal intensity, and the mean average for the two donors was plotted. Dose-response curves were fitted to a logistic model, and half-maximal effective concentrations (EC_{50}) were calculated using GraphPad Prism data analysis software v9.0.

For RNA-Seq experiments, 5×10^{5} purified human $CD8^{+}$ T cells were cultured in complete RPMI 1640 medium and stimulated for 4 or 24 hr with 1 μM IL-2 or 0.5 μM (equivalent stoichiometric quantity) Control IC or F10 IC. Total RNA was isolated using Direct-zol RNA Microprep Kit (R2061, ZYMO Research), and RNA-Seq libraries were prepared using 120 ng of total RNA and Kapa mRNA HyperPrep Kit (Kapa Biosystems). Each library was indexed using NEXTflex DNA Barcodes-24 (BIOO Scientific) and amplified by PCR. Amplified libraries between 250-400 bp were recovered using 2% E-Gel pre-cast gels (ThermoFisher), purified with Zymoclean Gel DNA Recovery Kit (Zymo Research), and quantified by Qubit 3 Fluorometer (ThermoFisher). Equal amounts of the indexed libraries were then pooled and sequenced with an Illumina Novaseq platform at the NHLBI DNA Sequencing Core.

RNA sequencing data analysis

Sequenced reads (50 bp, single end) were obtained with the Illumina CASAVA pipeline and mapped to the human genome hg38 (GRCh38, Dec. 2013) using Bowtie2 and Tophat2. Only uniquely mapped reads were retained. Raw counts that fell on exons of each gene were calculated and normalized using RPKM (Reads Per Kilobase per Million mapped reads). R Bioconductor package "edgeR" was used to identify differentially expressed genes between cells treated with

IL-2 and the ICs at the 4 hr and 24 hr time points. Gene expression heat maps were generated with the R package "pheatmap". Gene Set Enrichment Analysis (GSEA) was used to determine statistically significant overlaps between given pre-ranked genes and Molecular Signature Database (MSigDB).

In vivo immune cell subset expansion

For relative effector ($CD4^+$ T_{conv}, $CD8^+$ T, NK, NKT, γδ T) cell:T_{reg} proliferation studies, C57BL/6 mice were injected intraperitoneally with the molar equivalent of 0.075 mg/kg IL-2/dose of IL-2/602 complex (prepared by incubating IL-2 and 602 Ab at a 2:1 cytokine:antibody molar ratio in PBS for 15 min at room temperature), or molar equivalent of 0.075 mg/kg IL-2 /dose of 602 IC, F10 IC or Control IC (approx. 4 μg IC) in 250 μL of PBS, on days 1, 2, 3, and 4. Mice were randomly distributed into experimental groups, with approximately equal average body weight across groups. Mice were sacrificed on day 5 (24 hr after the last injection) by cervical dislocation, and spleens were harvested. Single-cell suspensions were prepared by homogenization (GentleMACS Dissociator, Miltenyi Biotec) and subjected to ACK red blood cell lysis buffer (Gibco).

For relative effector ($CD4^+$ T_{conv}, $CD8^+$ T, NK, NKT, γδ T) cell:T_{reg} proliferation studies in tumor-bearing mice, C57BL/6 or BALB/c mice were inoculated subcutaneously with 1×10^5 B16F10 cells or 2×10^5 CT26 cells, respectively, in 100 μL of sterile PBS on the shaved right anterior flank. Tumor-bearing mice were randomly distributed into experimental groups, with approximately equal average body weight across groups. C57BL/6 (B16F10) or BALB/c (CT26) mice were injected intraperitoneally with either PBS or the molar equivalent of 0.125 mg/kg IL-2 /dose of 602 IC, F10 IC or Control IC (approx. 4 μg IC) in 200 μL of PBS, on days 2, 4, 6 and 10 (B16F10) or 4, 6, 8 and 12 (CT26) following inoculation of the tumor cells. Mice were sacrificed on day 21 (B16F10) or day 25 (CT26) by cervical dislocation, and spleens and tumors were harvested. For the short-term immune cell subset expansion study, C57BL/6 (B16F10) mice were treated intraperitoneally with the molar equivalent of 0.125 mg/kg IL-2/dose of IL-2/602 complex (prepared by incubating IL-2 and 602 Ab at a 2:1 cytokine:antibody molar ratio in PBS for 15 min at room temperature), or molar equivalent of 0.125 mg/kg IL-2 /dose of F10 IC or Control IC (approx. 4 μg IC) in 200 μL of PBS, on days 2, 4, 6 and 10. Mice were sacrificed on day 11 (24 hr after the last injection) by cervical dislocation, and spleens and tumors were harvested. In all cases, single-cell suspensions were prepared by spleen homogenization (GentleMACS Dissociator, Miltenyi Biotec) or tumor dissociation (Tumor Dissociation Kit, Mouse, Miltenyi Biotec) and subjected to ACK red blood cell lysis buffer (Gibco).

The isolated splenocytes and tumor infiltrating lymphocytes were resuspended in modified PBE buffer (PBS with 2.5% fetal calf serum [FCS], 2.5 mmol EDTA) and blocked with 10% C57BL/6 mouse serum for 30 min on ice. The cells from each spleen were then split into 3 groups, which were each stained in modified PBE buffer for 30 min on ice with each of the following panels: CD4 T cell/T_{reg} panel, which included eFluor 450 anti-mouse CD3 (clone 17A2, ThermoFisher Scientific, 1:40), PerCP anti-mouse CD4 (clone RM4-5, BD Biosciences, 1:100),

and APC anti-mouse CD25 (clone PC61.5, ThermoFisher Scientific, 1:300); CD8/CD4 T cell panel, which included PE-Cy7 anti-mouse CD3 (clone 145-2C11, ThermoFisher Scientific, 1:40); PerCP anti-mouse CD4 (clone RM4-5, BD Biosciences, 1:100), BD Horizon V500 anti-mouse CD8 (clone 53-7.62, BD Biosciences, 1:80), APC anti-mouse CD44 (clone IM7, ThermoFisher Scientific, 1:500), eFluor450 anti-mouse CD62L (clone MEL-14, ThermoFisher Scientific, 1:40), FITC anti-mouse CD279 (PD-1) (clone RMP1-30, ThermoFisher Scientific, 1:40); and NK/NKT/γδ T cell panel, which included BD Horizon V500 anti-mouse CD3 (clone 500A2, BD Biosciences, 1:30), PE anti-mouse CD49b (clone DX5, ThermoFisher Scientific, 1:50), APC anti-mouse NK1.1 (clone PK136, ThermoFisher Scientific, 1:125) and eFluor 450 anti-mouse γδTCR (clone eBioGL3, ThermoFisher Scientific, 1:40). Cells were then washed twice and fixed in Fixation/Permeabilization Buffer (BD Biosciences) for 1 hr on ice. The T_{reg} panel cells were stained with PE anti-mouse/rat Foxp3 (clone FJK-16s, ThermoFisher Scientific, 1:100) for 30 min on ice, followed by two final washes and resuspension in modified PBE buffer. Samples were analyzed on a 4-laser (407/488/561/633 nm) BD Fortessa flow cytometer (BD Biosciences, Figure 7 A-C, Supplementary Figure 9 A-D, Supplementary Figure 10 and 11) or 5-laser (355/407/488/561/633 nm) Cytek Aurora spectral flow cytometer (Cytek, Supplementary Figure 12). Unmixing was performed using SpectroFlo software (Cytek). The percentages of splenic lymphocytes that were conventional $CD4^+$ T cells ($CD4^+$ T_{convs}; $CD3^+CD4^+Foxp3^-$) and T_{regs} ($CD3^+CD4^+CD25^+Foxp3^+$) were determined from the CD4 T cell/T_{reg} panel; the percentages that were $CD8^+$ T cells ($CD3^+CD4^-CD8^+$), memory phenotype $CD8^+$ T cells ($CD8^+$ T_{MPs}; $CD3^+CD4^-CD8^+CD44^+CD122^+PD\text{-}1^+$) and effector memory $CD4^+$ T cells ($CD4^+$ T_{EMs}; $CD3^+CD4^+CD44^+CD62L^-$), were determined from the CD8 T cell panel; the percentages that were NK cells ($CD3^-CD49b^+NK1.1^+$), NKT cells ($CD3^+CD49b^+NK1.1^+$ $\gamma\delta TCR^-$) and γδ T cells ($CD3^+\gamma\delta TCR^+$) were determined from the NK/NKT/γδ T cell panel (see gating strategy in Supplemental Figure 15). Data were analyzed using FlowJo ver. 10.6.1 software (Tree Star). The total number of splenocytes was acquired by measuring each single cell suspension prepared from spleen on an automated cell counter (EVE, NanoEnTek, Korea). The experiment was performed twice with similar results. Average ratios of the relative expansion of the indicated subsets are plotted.

Tumor therapy studies in mice

C57BL/6 or BALB/c mice were s.c. inoculated with 1×10^5 B16F10 cells or 2×10^5 CT26 cells, respectively, in 100 μL of sterile PBS on the shaved right anterior flank. Tumor-bearing mice were randomly distributed into experimental groups, with approximately equal average body weight across groups. For Supplemental Figure 9E, treatments were administered on days 4, 8, and 11 after tumor inoculation. For Figure 7, D-G, treatments were administered on days 2, 4, 6, and 10 after tumor inoculation for B16F10 models and days 4, 6, 8, and 12 after tumor implantation for CT26 models. For Supplemental Figure 9E, mice were treated i.p. with the molar equivalent of 0.125 mg/kg IL-2/dose of IL-2/602 complex (prepared by incubating IL-2 and 602 Ab at a 2:1 molar ratio of cytokine:antibody in PBS for 15 min at room temperature), or PBS alone. For Figure

7, D-G, mice were treated i.p. with the molar equivalent of 0.125 mg/kg IL-2 /dose of F10 IC (approx. 13.3 μg of IC) in 200 μL of PBS 0.665 mg/kg /dose of F10 IC or Control IC (molar equivalent of 0.125 mg/kg IL-2, approx. 13.3 μg IC) in 200 μL PBS, or with 200 μL of PBS alone The treatment regimen was prophylactic, i.e., the first treatment was applied before the tumors were developed (diameter of approx. 2-3 mm). The tumors were measured by caliper, and size was calculated as (shortest diameter)^2×(longest diameter)×0.5. The mice were euthanized on day 21 (B16F10) or 25 (CT26). No mice were excluded from analyses except those with unexpected death from nontumor reasons.

For the late-stage combinational treatment tumor study, BALB/c mice were inoculated subcutaneously with 2×10^5 CT26 cells in 100 μL of RPMI 1640 media (ATCC modification, Gibco) on the shaved right anterior flank. Tumor-bearing mice were randomly distributed into experimental groups, with approximately equal average body weight across groups. Mice were treated intraperitoneally on days 7, 10, and 13 post inoculation of the tumor cells with either PBS or a combination of anti-PD-1 antibody (0.5 mg/kg) and anti-CTLA-4 antibody (0.5 mg/kg), followed by intraperitoneal injections on days 14, 15, and 16 with either PBS or the molar equivalent of 0.125 mg/kg IL-2 of Control IC or F10 IC. The tumors were measured by digital caliper, and size was calculated as (shortest diameter)^2×(longest diameter)×0.5. No mice were excluded from analyses except those with unexpected death from nontumor reasons. Mouse body weights were recorded from an OHAUS Scout SPX123 portable balance.

Toxicity studies in mice

To assess the degree to which any treatment caused toxicity in the form of vascular leak syndrome (VLS), non-tumor-bearing C57BL/6 or BALB/c mice were injected i.p. on days 1, 2, 3, and 4 with 1.5 μg/dose IL-2, the molar equivalent of 0.075 mg/kg IL-2/dose of IL-2/602 complex (prepared by incubating IL-2 and 602 Ab at a 2:1 cytokine:antibody molar ratio in PBS for 15 min at room temperature), the molar equivalent of 0.075 mg/kg IL-2/dose of 602 IC, F10 IC, or Control IC in 250 μL of PBS, or with 250 μL of PBS alone. Mice were randomly distributed into experimental groups, with approximately equal average body weight across groups. Mice were sacrificed on day 5 (24 hr after the last injection) by cervical dislocation, and lungs were harvested. The difference in the wet weight and dry weight of the lungs, before and after lyophilization (16-24 hr, Concentrator Plus, Eppendorf), respectively, was calculated. The water content of the lungs was measured as the dry weight of lungs subtracted from the wet weight of the lungs. For ALT and AST analysis, BALB/c mice were sacrificed on day 5 by carotid cut, and all blood was collected into heparin-treated blood-collection vials, serum isolated, and analyzed for AST (IFCC; Beckman Coulter, OSR6109) and ALT (IFCC; Beckman Coulter, OSR6107) using Beckman Coulter AU480 clinical chemistry analyzer (Beckman Coulter, Czech Republic).

For the acute toxicity study shown in Supplemental Figure 13, liver toxicity was analyzed with SimpleStep ELISA (Abcam; AST, ALT and Albumin) or Bilirubin Colorimetric Assay Kit (Abcam) according to the manufacturer´s protocol. from sera of C57BL/6 mice s.c. inoculated with 1×10^5 B16F10 cells in 100 μL of DMEM media (Gibco) on the shaved right anterior flank.

Mice were injected intraperitoneally (i.p.) on day 2, 4, 6 and 10 post inoculation of the tumor cells with the molar equivalent of 0.125 mg/kg IL-2/dose of IL-2/602 complex (prepared by incubating IL-2 and 602 Ab at a 2:1 cytokine:antibody molar ratio in PBS for 15 min at room temperature), or molar equivalent of 0.125 mg/kg IL-2 /dose of F10 IC or Control IC (approx. 4 μg IC) in 200 μL of PBS. Blood was collected from orbital sinus on day 11 (24 hr after the last injection). Blood samples were incubated at room temperature for 1 hour and centrifuged at 3000×g for 10 minutes to remove cells. All assays were measured on a BioTek Synergy Mx microplate reader.

Cytokine levels from sera, acquired as described above, were analyzed with LEGENDplex Mouse Inflammation Panel (13-plex; Biolegend). Samples were analyzed by 3-laser (405/488/633 nm) Beckman Coulter Cytoflex flow cytometer (Beckman Coulter) and evaluated by LEGENDplex™ data analysis software.

AUTHOR CONTRIBUTIONS

JT and JBS conceived the project. EKL, JT, JRG, OV, WJL, and JBS contributed to study design. EKL, JT, JRG, J-XL, PL, TH, MH, and OV performed experiments, collected and analyzed data, with assistance from MIL, MJP, ERJ, LT, SDC, SH, PRS, and CSF. MK determined and provided the MAB602 sequence. JT, OV, MK, WJL, BS, GR, and JBS acquired funding in support of this research. JBS supervised the project. EKL, JT, JRG, and JBS wrote the manuscript, with input from all co-authors. EKL and JT are listed as co-first authors; EKL is listed first because of her contributions to project design, experimental work, and manuscript preparation. All authors read and approved the final version of the manuscript.

ACKNOWLEDGEMENTS

This study was supported by the Department of Defense (W81XWH1810735 and W81XWH21P0031 to JBS), the National Institutes of Health (R21CA249381, R01EB029455, and R01EB029341 to JBS), Maryland Stem Cell Research Fund grant (2019-MSCRFD-5039 to JBS), a V Foundation Scholars award (V2018-005 to JBS), a Bladder Cancer Action Network Award (GC 10790-01 to JBS), an Emerson Collective Cancer Research Fund award to JBS, a Willowcroft Foundation grant to JBS, a Johns Hopkins University Catalyst award to JBS, the Czech Science Foundation (20-13029S to JT and OV), the Charles University (GAUK 285423 to OV), the Institute of Biotechnology of the Czech Academy of Sciences (RVO 86652036 to JT, TH, MH, and BS), the Institute of Microbiology of the Czech Academy of Sciences (RVO 61388971 to JT and MK), the project National Institute for Cancer Research (Programme EXCELES, ID LX22NPO5102 to JT and BS) - Funded by the European Union - Next Generation EU, and the Czech Centre for Phenogenomics at the Institute of Molecular Genetics (RVO 68378050 and MEYS CR LM2018126 to JT, TH, MH, and BS). The work was supported by the Division of Intramural Research, National Heart, Lung, and Blood Institute, and the Division of Intramural Research, National Cancer Institute. EKL was supported by the National Institutes of Health Training Grant K12 GM123914 01A1. JRG was supported by an American Cancer Society postdoctoral research fellowship. PRS was supported by a National Science Foundation Graduate Research Fellowship Program Award. CSF was supported by National Institutes of Health T32 GM135131.

REFERENCES

1. Yodoi J, et al. TCGF (IL 2)-receptor inducing factor(s). I. Regulation of IL 2 receptor on a natural killer-like cell line (YT cells). *J Immunol*. 1985;134(3):1623–1630.

2. Kuziel WA, et al. Unexpected effects of the IL-2 receptor alpha subunit on high affinity IL-2 receptor assembly and function detected with a mutant IL-2 analog. *J Immunol*. 1993;150(8):3357–3365.

3. SPANGLER J, et al. Methods and materials for targeted expansion of immune effector cells. 2020. https://patents.google.com/patent/WO2020264321A1/en?q=(Ludwig+leonard%2526inventor). Accessed June 26, 2023.

4. Whitlow M, et al. 1.85 Å structure of anti-fluorescein 4-4-20 Fab. *Protein Eng Des Sel*. 1995;8(8):749–761.

5. Lo M, et al. Effector-attenuating Substitutions That Maintain Antibody Stability and Reduce Toxicity in Mice *. *J Biol Chem*. 2017;292(9):3900–3908.

6. Boder ET, Wittrup KD. Yeast surface display for screening combinatorial polypeptide libraries. *Nat Biotechnol*. 1997;15(6):553–557.

7. Chao G, et al. Isolating and engineering human antibodies using yeast surface display. *Nat Protoc*. 2006;1(2):755–768.

8. Vaněk O, et al. Production of recombinant soluble dimeric C-type lectin-like receptors of rat natural killer cells. *Sci Rep*. 2019;9(1):17836.

9. Hayes D, Laue T, Philo J. Program Sednterp: sedimentation interpretation program. 1995.

10. Schuck P. Size-Distribution Analysis of Macromolecules by Sedimentation Velocity Ultracentrifugation and Lamm Equation Modeling. *Biophys J*. 2000;78(3):1606–1619.

11. Brautigam CA. Calculations and Publication-Quality Illustrations for Analytical Ultracentrifugation Data. *Methods in Enzymology*. Elsevier; 2015:109–133.
