# Supplementary figures and images for: Engineered cytokine/antibody fusion proteins improve IL-2 delivery to pro-inflammatory cells and promote antitumor activity
Source: JCI Insight. 2024 Sep 24;9(18):e173469. doi: 10.1172/jci.insight.173469 (PMC11457862; doi:10.1172/jci.insight.173469)

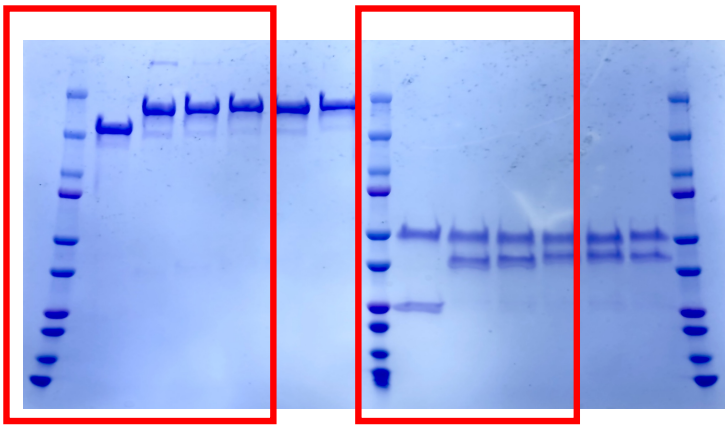

Figure 1B.

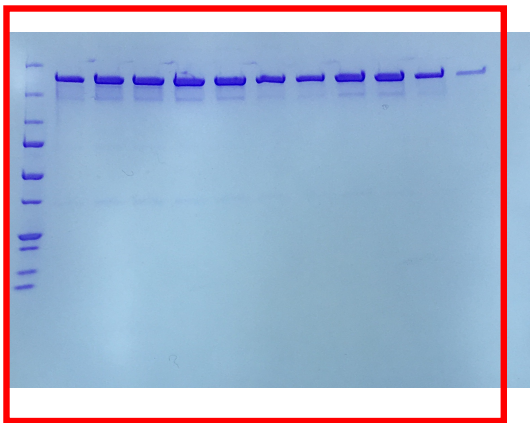

**Supplemental Figure 1D.**

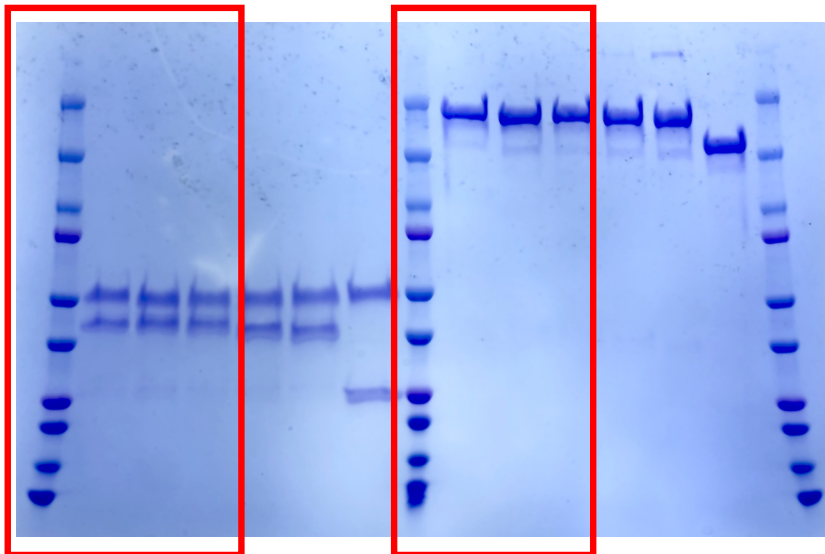

Supplemental Figure 3K.

Supplement: Unedited blot and gel images [file jciinsight-9-173469-s261.pdf]
